# Selection Signatures in Chinese Sorghum Reveals Its Unique Liquor-Making Properties

**DOI:** 10.3389/fpls.2022.923734

**Published:** 2022-06-09

**Authors:** Liyi Zhang, Yanqing Ding, Jianxia Xu, Xu Gao, Ning Cao, Kuiying Li, Zhou Feng, Bing Cheng, Lengbo Zhou, Mingjian Ren, Xiaochun Lu, Zhigui Bao, Yuezhi Tao, Zhanguo Xin, Guihua Zou

**Affiliations:** ^1^Guizhou Institute of Upland Crops, Guizhou Academy of Agricultural Sciences, Guiyang, China; ^2^College of Agriculture, Guizhou University, Guiyang, China; ^3^Institute of Sorghum Research, Liaoning Academy of Agricultural Sciences, Shenyang, China; ^4^Shanghai OE Biotech Co., Ltd., Shanghai, China; ^5^Institute of Crop and Nuclear Technology Utilization, Zhejiang Academy of Agricultural Sciences, Hangzhou, China; ^6^Plant Stress and Germplasm Development Unit, Cropping Systems Research Laboratory, USDA-ARS, Lubbock, TX, United States

**Keywords:** sorghum, selective sweep, population structure, genome-wide association study (GWAS), liquor-brewing related traits

## Abstract

Chinese sorghum (S. bicolor) has been a historically critical ingredient for brewing famous distilled liquors ever since Yuan Dynasty (749 ∼ 652 years BP). Incomplete understanding of the population genetics and domestication history limits its broad applications, especially that the lack of genetics knowledge underlying liquor-brewing properties makes it difficult to establish scientific standards for sorghum breeding. To unravel the domestic history of Chinese sorghum, we re-sequenced 244 Chinese sorghum lines selected from 16 provinces. We found that Chinese sorghums formed three distinct genetic sub-structures, referred as the Northern, the Southern, and the Chishui groups, following an obviously geographic pattern. These sorghum accessions were further characterized in liquor brewing traits and identified selection footprints associated with liquor brewing efficiency. An importantly selective sweep region identified includes several homologous genes involving in grain size, pericarp thickness, and architecture of inflorescence. Our result also demonstrated that pericarp strength rather than grain size determines the ability of the grains to resist repeated cooking during brewing process. New insight into the traits beneficial to the liquor-brewing process provides both a better understanding on Chinese sorghum domestication and a guidance on breeding sorghum as a multiple use crop in China.

## Introduction

Sorghum [S. *bicolor* (L.) Moench, *2n* = *2x* = *20*] is the fifth largest and wildly adaptable crop in the world. It is cultivated in more than 100 countries and regions around the world. It is mainly used for food, feed, fiber, brewing, and bioenergy. Modern sorghum, like other domesticated crops, has been improved as a consequence of long-term artificial selection (Mace et al., 2013; Smith et al., 2019). The diverse distribution and extensive morphological variation observed in multiple sorghum species imply a remarkable history of divergence and evolution. The northeast quadrant of Africa is believed to be the primary origin and diversity center of sorghum. Shortly after its early domestication around 6,000 years BP (Before present), sorghum spread to India (Doggett, 1988; Dahlberg and Wasylikowa, 1996; Winchell et al., 2017). The Indian subcontinent is considered as the secondary center of origin of sorghum, and the earliest domesticated sorghum appeared in the late Harappan, approximately 4,000–3,700 years BP (Fuller, 2003; Boivin and Fuller, 2009). Chinese sorghum, the Kaoliangs, has a cultivation history of ∼2,200 years with morphologies characteristic of bicolor type. It was inferred as the early bicolor variants introduced from northeast India (Mann et al., 1983; Doggett, 1988). It is widely believed that the domesticated sorghum was transmitted eastward from India to China either *via* land or sea routes (Fuller, 2003; Boivin and Fuller, 2009). Recently, molecular genetics studies suggested that Chinese sorghum may be of African origin (Li et al., 2010; Zhang et al., 2011). This question remains unsolved based on the current evidence from morphology, agronomy, and even genomic features.

In China, sorghum has been a key ingredient for brewing liquor for ∼750 years (Shinoda, 1958; Han, 2012). Ancient Chinese, as known in the world, is the first to master the knowledge of using sorghum to brew distilled liquor (Awika and Rooney, 2004; Zhao, 2019b). Currently, sorghum is widely cultivated across agroclimatic zones from the south to the north in China, and more than 80% of sorghum grain is used to brew liquor (Chen et al., 2019). Techniques to brew distilled liquor were developed in the Tang and Song Dynasties (1,402–749 years BP), and commercialized in the Yuan Dynasty (749–652 years BP) (Joseph, 2000). Almost all Chinese famous brands of distilled liquors, such as Moutaijiu and Langjiu (Moutai-aroma liquor), Luzhoulaojiao, Wuliangye (strong-aroma liquor), and Fenjiu (light-aroma liquor), etc., are brewed with sorghum grain as a key ingredient. The first two flavored liquors are mainly from southwest region, a principal area producing liquors since ancient times. The liquor-making processes of different flavor liquors have different requirements for the physical properties of sorghum grains. The Maotai-aroma liquor produced in the Chishui River Basin in the Southwest has a complex and special brewing process, which requires nine times of steaming and boiling, eight times of fermentation, and seven times of liquor extraction, hence, the sorghum grains must be able to resist to the repeatedly steaming and stirring. The brewing process of other flavored liquors only requires one or two times of cooking to extract the liquor, which desires sorghum grains that can be cooked and broken easily (Sun, 2019). This remarkably domesticated history by human-intervention raises the question to what extent the artificial and adaptive selections for liquor-brewing shaped the local Chinese sorghum population.

High-throughput resequencing technology, combined with comprehensive statistical analysis methods, provides a powerful tool to uncover the mysteries of plant domestication. Based on deep-coverage of whole-genome sequence of accessions from various regions, the origin and domestication history of several crops have recently been uncovered, including ancient sorghum (*S. bicolor ssp. bicolor* (L.) Moench.) (Smith et al., 2019), Tibetan barley (*Hordeum vulgare* L., qingke) (Zeng et al., 2018), castor (*Ricinus communis*) (Fan et al., 2019; Xu et al., 2021), pear (*Pyrus*) (Wu et al., 2018), chickpea (*Cicer arietinum L*.) (Varshney et al., 2019), etc. Therefore, we investigated the data from whole genome resequencing of 333 sorghum accessions (244 sequenced in this study plus 89 genomes available publicly) to further explore the domestication history and genome-wide selective footprints on Chinese sorghum population. In addition, we described a genome wide map of SNP variation and traced patterns of sorghum diffusion to diverse agroclimatic regions. Our study demonstrated that Chinese sorghum was first introduced to south China from Indian and gradually spread to the north China. Furthermore, we identified genes underlying natural variation in traits related to baijiu-making process.

## Materials and Methods

### Plant Material and Phenotyping

A worldwide sorghum collection of 333 accessions was used in this study, of which a panel of 244 was mainly composed of Chinese sorghum from the Center for Crop Germplasm Resources, Institute of Crop Sciences, Chinese Academy of Agricultural Sciences (Beijing, China), and Institute of Upland Crops, Guizhou Academy of Agricultural Science (Guiyang, China). Another panel of 89 accessions included 38 wild sorghum and 51 landraces/cultivars. Wild sorghums were mostly from Africa, while cultivated sorghums were from Africa and Asia, maintained in the Institute of Botany, Chinese Academy of Sciences (Beijing, China).

From 2018 to 2020, the panel of 244 accessions were grown in Guiyang, Guizhou Province; Lingshui and Ledong, Hainan Province; and Hangzhou, Zhejiang Province in China. Five traits related with brewing properties were thousand grain weight (TGW), grain length (GL), grain width (GW), pericarp thickness (PT) and testa thickness (TT). TGW, GL, and GW were measured by a Crop Grain Appearance Quality Scanning Machine (SC-E, Wanshen Technology Company, Hangzhou, China). PT and TT were measured by a Digital Microscope (VHX-7000, KEYENCE Technology Company, Shanghai, China).

### DNA Extraction and Sequencing

Genomic DNAs of 244 accessions were extracted following the cetyltrimethylammonium bromide (CTAB) method and quantified by Qubit^®^ 2.0 fluorescent meter (Invitrogen, Carlsbad, United States). The quality of the DNA was determined by electrophoresis in 0.8% agarose gel running at 100 V for 40 min. The DNA fragments around 350 bp were randomly generated by Covaris ultrasonic crushing apparatus. The constructed library was used for paired-end (PE150) sequencing on Illumina HiSeq 4000 sequencing platform by Beijing Novogene technology co., Ltd. (Beijing, China). The 89 public sequencing data for African and Asian sorghums were obtained from SorGSD (Zhang et al., 2018)^1^.

### Reads Mapping and Variants Calling

The raw paired-end reads were trimmed and filtered with a sliding window of size 4, with average Phred score scale of 20 within the window using fastp1 (version: 0.20.0) (Chen et al., 2018). The clean reads of 333 accessions were mapped to the *Sorghum bicolor* genome (Phytozome v12^2^) (Goodstein et al., 2012) using bwa-mem (version: 0.7-17) (Li and Durbin, 2009) with default parameters. After alignment, Picard tools (version: 2.18.17^3^) were used to remove PCR duplicates according to the mapping coordinates.

The variation detection followed the best practice workflow recommended by Genome Analysis Toolkit (GATK4. version 3.8.1) (McKenna et al., 2010). In brief, the variants were called for each accession by the GATK HaplotypeCaller. A joint genotyping step for comprehensive variations union was performed on the gVCF files. Two subsets of the sorghum SNPs were defined using the following filtering criteria: (1) the basic set of 3,298,433 SNPs were created with exclusion of non bi-allelic, > 20% missing calls and MAF < 5%; (2) a core SNP set of 526,946 SNPs derived from the basic SNP set using a two-step linkage disequilibrium pruning procedure with PLINK (version: 1.9) (Purcell et al., 2007) with parameters “–indep-pairwise 50 5 0.5.”

SNPs and Indels were annotated according to the BTx623 genome using the SNPeff6 (version: 4.3T) (Cingolani et al., 2012). SNP and InDel densities across each chromosome were counted with 500 kb sliding window using VCFtools (version: 0.1.17) software (Danecek et al., 2011).

### Phylogenetic and Population Analyses

A phylogenetic tree was constructed from the core SNP set by using the neighbor-joining method in the program PHYLIP (version: 3.697^4^), and IBS distance matrices were calculated using PLINK. The resulting phylogenetic tree was visualized using the online tool iTOL (version: 4.0) (Letunic and Bork, 2019).

Principal Components Analysis (PCA) was performed with the smartPCA program from EIGENSOFT package (version: 6.1.4) (Price et al., 2006) with the core SNP set, and the first three eigenvectors were plotted by scatterplot3d R package. Population structure was inferred using the fastSTRUCTURE (version: 1.0) program (Raj et al., 2014). To explore the convergence of individuals, we predefined the number of genetic clusters K from 2 to 12 and ran the cross-validation error (CV) procedure. fastSTRUCTURE was then run again on the whole core SNP set 10 times with varying random seeds; the Q-matrices were aligned using pong13 software and clustered based on similarity. Then, the matrices belonging to the largest cluster were averaged to produce the final matrix of admixture proportions.

Global and chromosomal *r*^2^ for each sorghum group were evaluated using PopLDdecay (version: v3.41^5^) (Zhang et al., 2019) with default setting.

### Genome Scanning for Selective Sweep Signals

We performed a genetic differentiation (*Fst*), nucleotide diversity (π) and Tajima’D based cross approach to investigate the selection signals across the whole genome. A 10 kb sliding window with 10 kb step approach was applied to quantify *Fst*, π and Tajima’D by using VCFtools software (Danecek et al., 2011). The candidates that meet both top 5% of the three tests were selected as selective signals.

### Genome-Wide Association Study

The association analysis was performed with the GLM and Blink statistical methods implemented in Genome Association and Prediction Integrated Tool package (GAPIT version: 3.0)^6^. The first three PCs derived from whole-genome SNPs were used as fixed effects in the mixed model to correct for stratification. The random effect was estimated from the groups clustered based on the kinship among all accessions. We defined the whole-genome significant cutoff with the adjusted Bonferroni test threshold, which was set as *P* = −log_10_(0.05/2,015,850) = 7.6. LD analysis and inference of the haplo-type blocks containing peak SNPs to document the potential candidate genes responsible for traits were performed in LDBlockShow (version: 1.39) (Dong et al., 2021).

### Sequence Analysis of the Candidate Gene

The candidate gene (Sobic.002G228600) from 20 accessions with extreme phenotypes were amplified and sequenced by Sanger sequencing technology in Platinum Biotechnology Co., Ltd. (Shanghai, China). The raw sequences were assembled using DNASTAR Lasergene (version: 7.10) and alignments for the sequence dataset were performed by MAFFT (version: 7.48) to identify the variations. The ExPASy translate tool^7^ was used to convert nucleotide sequence into amino acid sequence, the sequencing results are shown in Supplementary Figure 4. The primers1 information: 5′-TTGGACAGTGTTGGACTCTC-3′ and 5′-CAGGAAGTAGGGATGGATCA-3′, the primers2 information: 5′-TCGTCGTGTCGGTGGAATAC-3′ and 5′-TGTAACTCAGCACGCAACAA-3′.

### RNA-Seq and Data Analysis

Two sorghum cultivars, “654” and “LTR108” were chosen for RNA sequencing (RNA-seq). Young panicles samples were collected at the S1, S2, and S3 stages, which corresponded to less-than 5 cm in length, 10–15 cm in length, and 5 days after heading, respectively. Total RNA was isolated using a protocol reported previously (Zou et al., 2020). A total of 1 μg RNA was used for library construction with an NEBNext^®^UltraTM RNA Library Prep Kit for Illumina^®^ (NEB, United States) according to the manufacturer’s instructions. RNA sequencing was performed on NovaSeq 6000 platform. Clean reads were mapped to the sorghum reference genomes V3.1.1 on Phytozome database^8^ using Hisat2 (version: 2.2.1). The number of reads mapped to each gene was counted using HTSeq software (version: 0.9.1). Gene expression based on fragments per kilo-base of exon per million fragments mapped (FPKM) was next obtained through Cufflinks (version: 2.2.1). Each sample consisted of three biological replicates.

### Experiment of Resistance to Cooking

We performed the cooking experiment on 16 glutinous sorghums whose grain size and shape, thickness of pericarp and testa were distinctly dissimilar. A volume of the grains (V_0_) with weight of 10 g of sorghum grains were measured by the drainage method in a 150 mL beaker. Beakers of glutinous and non-glutinous sorghum varieties were filled with 85°C water on standing in a thermostat for 8 and 10 h, respectively. Subsequently, the grain volume (V_t_) was measured once again without the supernatant, and the expansion rate was thus obtained [Expansion rate = (V_t_ - V_0_)/V_0_ × 100%]. The crack rate was observed and counted from 100 random grains (Liu et al., 2012).

## Results

### Whole Genome Resequencing of Chinese Sorghum

**FIGURE 1:**
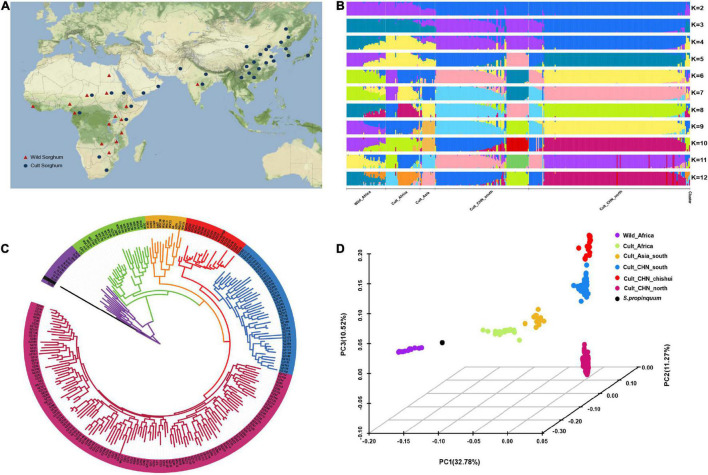
Germplasm origin and population genetic analysis. **(A)** Geographic distribution of cultivated and wild sorghum accessions represented by red triangle and blue circle, respectively. **(B)** Genetic structure of sorghum accessions as inferred with structure for K = 2 to 12. **(C)** Neighbor-joining tree for sorghum accessions. The six subpopulations defined based on structures analysis are indicated by different colors. Purple, green, orange, red, blue, and rose indicate wild sorghum, and cultivated accessions from African, South Asian, Chinese Chishui, South and North China, respectively. **(D)** Principal component analysis of sorghum accessions, showing the first three principal components. The dot colors correspond to the phylogenetic tree grouping.

To investigate the population structure of Chinese sorghum accessions, we sequenced 244 sorghum landraces/cultivars, which were collected extensively from 16 sorghum planting provinces across China (151 from North China, 91 from South China, and 2 from abroad). To better address the domestication history of sorghum in China and identify the different genomic regions under domestication, we also supplemented our sequencing data with 89 publicly available sorghum genome sequences, including one accession of *Sorghum propinquum*, a wild relative of sorghum (the same subgenera with *S. bicolor*), 37 wild sorghum mainly from Africa, and 51 cultivated sorghum from Africa and Asia (Zhang et al., 2018). In total, a collection of 333 sorghum accessions was analyzed in the present study (Figure 1A and Supplementary Table 1).

The whole-genome resequencing generated a total of ∼12.04 G paired-end reads 150 bp in length, with an average sequencing depth of ∼7.04 × and an average genome coverage of ∼86.8% (Supplementary Table 2). After mapping to the sorghum reference genome BTx623 (Paterson et al., 2009; McCormick et al., 2018) and single nucleotide polymorphism (SNP) calling, we obtained 3,298,433 high-quality SNPs and 429, 794 InDels (insertions-deletions) from the 333 sorghum accessions. Among these variations, 203,278 (4.2%) SNPs were in coding regions, with a non-synonymous-to-synonymous substitution ratio of 1 (86,150 and 87,333 for non-synonymous and synonymous, respectively).

### Genetic Structuration, Phylogenetic and Principal Component Analysis

The population structure of the whole data set (*N* = 333) was analyzed by *fast*STRUCTURE with K ranging from 2 to 12 (Figure 1B). The barplots were inspected visually to identify the well delimited clusters that could be biologically relevant. Although the optimal number of clusters is inferred by the K selector as 5, the finest biologically relevant population structure was identified at K = 10. The number of main clusters remained as six, including the wild relatives, cultivated sorghum in Africa, Southern Asia, Southern China, Northern China, and Chishui in Southwest China, which were apparently consistent with the evolutionary history of sorghum and corresponded to the species and/or geographical regions (Fuller, 2003; Boivin and Fuller, 2009). We therefore considered these six clusters as the most relevant genetic structure and estimated the sorghum individual membership at K = 10.

We considered hereafter a genotype to be unequivocally assigned to a population when its assignment probability was ≥ 95% to one of the six genetic clusters at K = 10, which was the case for 71.5% of individuals (*N* = 238). It is important to set assignment thresholds in order to distinguish recent hybridization (or recent gene flow) from ancient gene flow, and the threshold we set appeared to be optimal (Groppi et al., 2021). Our maximum likelihood based phylogenetic dendrogram clearly distinguished six clusters (Figure 1C). The wild sorghum (purple) and African cultivated lines (green) split into two clades. The latter includes two forms of sorghums in north and south Africa, which was closely followed by South Asian sorghum (orange) and Chinese sorghum. Among Chinese accessions, sorghum from the Chishui (red) had a closer relationship with South Asian sorghum, while Northern sorghum (rose) was clearly separated from South Asian sorghum.

The above classification based on *fast*STRUCTURE was also supported by principal component analysis. A total of 53.57% of the genetic variation was explained by the first principal component analysis (Figure 1D). The wild sorghum (purple), African cultivated sorghum (green), South Asian cultivated sorghum (orange) and Chinese accessions were well distinguished along the PC_1_ axis, while the sorghum of Northern China (rose), Southern (blue) and Chishui (red) were obviously separated by PC_3_ axis. South China sorghum were observed closer to South Asian sorghum than to the North China sorghum, which was supported by the phylogenetic relationship.

### Genetic Diversity, Linkage Disequilibrium and Selective Sweep on Sorghum Collections

**FIGURE 2:**
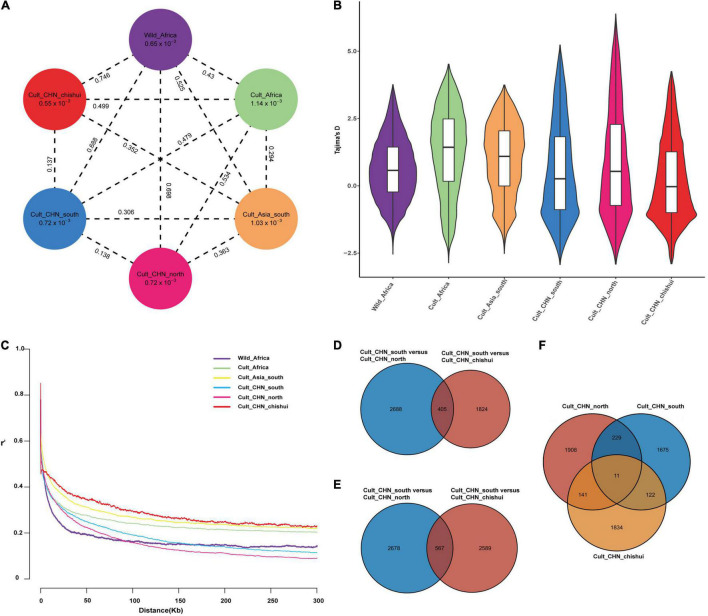
Evolution history of Chinese sorghum. **(A)** Nucleotide diversity (π) and population divergence (*Fst*) across the six populations. The values in the circle represent the mean value of π for each group; the values on each dotted line indicate pairwise *Fst* between populations. **(B)** Violin plots showing Tajima’s D estimation in six sorghum populations. **(C)** LD decay in different sorghum populations. **(D–F)** Venn diagrams representing the number of unique and shared domestication genes among Chinese sorghum populations, based on the selected thresholds top 5% of *Fst*, π ratio values, and Tajima’s D negative values respectively.

Genetic divergence among the inferred six genetic groups of sorghum was firstly estimated by the genetic distance (Fixation index values, *F*_*st*_) across the genome (Figure 2A). The wild African sorghum had a lowest *Fst* (0.43) compared with the cultivated African sorghum, but the highest *Fst* (0.75) against the Chinese Chishui sorghum. However, the cultivated African sorghum showed lower *Fst* (0.48) with the Chinese sorghum in the south than that (0.53) of the north. These results were consistent with the phylogenetic and structure analyses.

We further implemented the mean nucleotide diversity (π) and Tajima’s D to address the genetic diversity of the collections (Figures 2A,B). The π in African and Asian cultivated sorghum genomes were 1.14 × 10^–3^ and 1.03 × 10^–3^, respectively. Low levels of π diversity were found in Chinese sorghum from the Northern, the Southern, and the Chishui sub-clusters (7.21 × 10^–4^, 7.18 × 10^–4^, and 5.52 × 10^–4^, respectively). More than half of Tajima’s D values observed from Chishui sorghum genome were negative.

In our collections, the LD extended to background level at ∼50 kb in wild African sorghum, and at ∼100, ∼110, and ∼ 200 kb for African, South-Asian and Chinese cultivated sorghum, respectively (Figure 2C). The LD varied on different chromosomes and in different sorghum populations (Supplementary Figure 1). The wild African sorghum possessed rapid LD declines on both chromosome 6 and 10. The Southern sorghum appeared to have higher LD level than the Northern sorghum, and the Chishui sorghum showed the lowest LD decay on chromosome 5.

To identify the potential domestication signatures in Chinese sorghum, we summarized the selective sweeps within 10-kb genomic intervals and scored significant selection by top 5% outliers in *Fst*, π ratio and Tajima’s D tests. Pairwise *Fst* statistics, respectively, revealed 3,093 and 2,229 candidate genes from the sweeps between Southern and Northern sorghum, and between Southern and Chishui sorghum, of which 405 candidate genes were shared (Figure 2D and Supplementary Figure 2). Meanwhile, candidate genes behind the selection signatures were identified as 3,245 by π*_*Southern*_*/π*_*Northern*_* ratio and as 3,156 by π*_*Southern*_*/π*_*Chishui*_* ratio, of which 567 candidate genes overlapped in the genomic regions (Figure 2E and Supplementary Figure 2). Comparing the overlapping candidate genes between *Fst* and π ratio methods, 1,050 and 285 identical genes were found between Northern and Southern sorghums and Southern and Chishui sorghums, respectively. Additionally, the negative Tajima’s D indicator was used to investigate the signals of the positive selections. We identified 2,289, 2,036, and 2,108 candidate genes for Southern, Northern and Chishui sorghum, respectively. Sorghum accessions from the Southern China shared 229 selective candidate genes against those from the Northern China and shared 122 candidate genes with Chishui sorghum. Totally, eleven candidate genes behind positive selection were in common among the three Chinese sorghum groups (Figure 2F and Supplementary Figure 3).

To understand putative functions of the strong selective sweep signals among Chinese populations, we investigated the annotations for the top 1‰ of the domestication genes identified by three methods. Only about 20% of these domesticated genes were matched to putative functions due to the lack of annotation. Overall, around half of the important genes are related to biotic and abiotic stress resistance, such as salt tolerance, cadmium tolerance, disease resistance, etc., while approximately 30% orthologous genes are associated with yield, quality, and plant type. The functions of important domestication genes did not differ significantly between populations based on *Fst* and π ratio, whereas the functions of important domesticated genes were quite distinct in different groups based on Tajima’D metric. There were more key domestication genes related to panicle structure, tillering, height, and yield in southern and Chishui populations compared with those in northern population (Supplementary Table 3).

### Impact of Selection for Traits Associated With Liquor-Brewing on Sorghum Genome

Since more than 30 processes are required to brew Maotai-flavor liquor in the Chishui River Basin in Southwest China, the sorghum grains are required to have good cooking resistance. GWAS was thence performed to determine signals for the domestication traits-related brewing properties, based on surveying grain physical properties TGW, GL, GW, PT, and TT of the 244 Chinese landraces/cultivars in six environments from 2018 to 2020 (Supplementary Table 4). Totally, 66 significant SNP loci were identified on the 15 genome regions (Supplementary Table 5).

**FIGURE 3:**
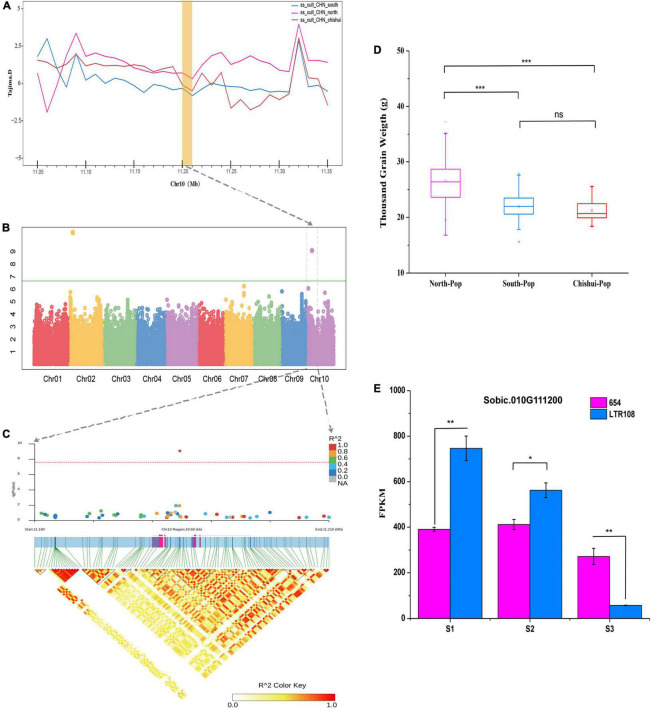
Candidate gene (Sobic.010G111200) for TGW based on GWAS analysis. **(A)** Selective sweeps region for TGW based on Tajima’s D values among the South population, North population and Chishui population. **(B)** Manhattan plots for TGW using phenotype data from the 244-accession panel in Guiyang in 2019. **(C)** Local Manhattan plot (upper) of TGW and linkage disequilibrium (LD) heat map (lower). **(D)** Comparisons of TGW among South, North, and Chishui populations (***P < 0.0001, **P < 0.01, *P < 0.05, ns indicates no significance, Student’s *T*-test). **(E)** Expression pattern of total Sobic.010G111200 in 654 (smaller grain) and LTR108 (bigger grain) in various stages using transcriptome sequencing: S1, young panicle < 5 cm in length; S2, young panicle 10–15 cm in length; S3, panicle 5 days after heading. FPKM: Fragments per kilobase of transcript per million mapped reads.

Two significant GWAS signals affecting both TGW and GW between Southern and Northern population during domestication were mapped in the selection sweeps associated with traits for liquor brewing were identified in this study (Supplementary Tables 3, 5). One important locus at the position 47,459,472 bp on chromosome 9 fell into a LD block including several genes, of which the gene Sobic.009G124200 (47,743,018 bp) is one of the strongest domestication genes based on *Fst* metric. This gene is an ortholog of the rice *SMALL ORGAN SIZE1* (*SMOS1*), which encodes an AP2-Type transcription factor acting as an auxin-dependent regulator for cell expansion during grain development (Aya et al., 2014). Another significant signal for TGW and GW was located on chromosome 10 at 11,199,795 bp position and underwent selection in both Southern and Chishui population during domestication based on Tajima’D metric (Figures 3A,B). One gene Sobic.010G111200 (11,197,868 bp) fell within LD block of significant loci (Figure 3C) and is orthologous to rice Os*GASR7*/*GW6* that encodes a GA-regulated GAST family protein and positively regulates grain width and weight (Shi et al., 2020). As expected, Chishui and Southern sorghums have smaller grains than Northern sorghum (Figure 3D). The mRNA expression of Sobic.010G111200 in developing panicles of less than 5-cm-long was significantly higher in cultivar LTR108 (larger grain) than in cultivar 654 (smaller grain), while mRNA levels in LTR108 dropped dramatically with panicle development and was even lower than the level of 654 after heading (Figure 3E).

**FIGURE 4:**
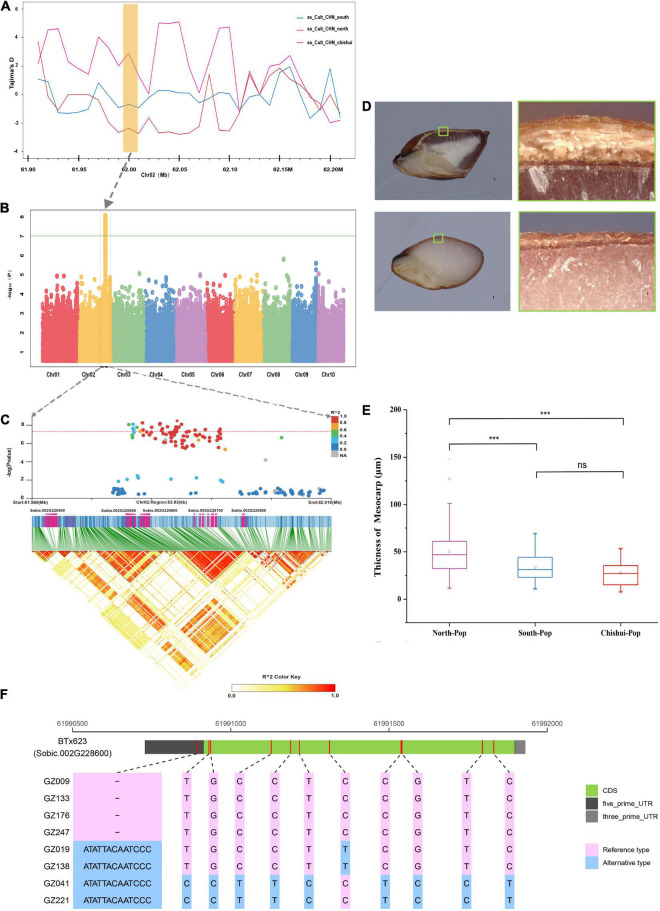
Candidate gene for pericarp thickness based on genome-wide association study analysis. **(A)** The selective sweep analysis of pericarp thickness genes based on Tajima’s D values among the South population, North population and Chishui population. **(B)** Manhattan plots for pericarp thickness using phenotype data from the 244-accession panel in Ledong in 2020. **(C)** Local Manhattan plot (upper) of pericarp thickness and linkage disequilibrium (LD) heat map (lower). **(D)** Longitudinal sections of intact seeds from thin pericarp accession in upper right image and thick pericarp sorghum accession in lower right image. The upper and lower images on the left present close-ups of the pericarp under the microscope, respectively corresponding to the small boxes in the right images. **(E)** Comparisons of pericarp thickness among South population, North population, and Chishui population (***P < 0.0001, ns indicates no significance, Student’s *T*-test). **(F)** The putative candidate gene model of pericarp thickness for eight extreme phenotypes. The red bar marks the mutant position between reference type and alternative type. INDEL in 5′-UTRs with pink indicates the reference genotype (thinner pericarp), while that with blue indicates the alternative genotype (thicker pericarp).

We also identified two GWAS signals for TT and PT (Supplementary Table 5). A GWAS signal associated with TT was detected on chromosome 2 at 8,300,524 bp position, which is clearly distinct from the previously reported *B2* locus (60.54–60.59 Mb) that control the presence of testa (Dufour et al., 1997; Rami et al., 1998; Mace et al., 2019). In addition, a significant signal was identified for pericarp thickness on chromosome 2 (61.99 Mb) that is close to the previously reported *Z* gene locus (57.03–59.10 Mb), which is related to the thickness of pericarp and pearly pericarp (Tao et al., 1998; Boivin et al., 1999; Mace et al., 2019). The signal was located in a large genomic region (140 kb, 61.96–62.10 Mb) of the strongly selective sweep during domestication in Chishui population based on Tajima’D metric (Figures 4A,B). The phenotypic distribution indicated that the pericarp of Chishui and southern accession was thinner than that of northern accession (Figures 4D,E). The significant SNP loci fell within a high LD block including two genes (Sobic.002G228600 and Sobic.002G228700) (Figure 4C). By comparing the sequence mutations of Sobic.002G228600 gene between extreme phenotypic varieties, we detected an insertion and deletion of “CATATTACAATCC” in the 5′-UTR in accessions with thin and thick pericarp, respectively (Figure 4F and Supplementary Figure 4).

### Relationship Between Baijiu-Brewing Process and the Physical Properties of Grains

Chishui sorghum has consistently thinner pericarp (exocarp and pericarp) than the other two Chinese populations (Figure 3E). Correlation analysis revealed that sorghum pericarp thickness showed significant positive association with TGW, GL and GW in six environments, with the average Pearson coefficient (*R* value) of 0.32, 0.24 and 0.26 (*P* < 0.01), respectively (Supplementary Table 6). The cooking experiment was performed to investigate the relationship between resistance to cooking with grain size and thickness of pericarp and testa. For glutinous sorghum, grain with thinner pericarp and testa generally had lower the rate of expansion and crack and presented better resistance to cooking (Table 1).

**TABLE 1:** Results of cooking experiment for glutinous sorghum.

Name	Pericarp thickness	Testa thickness	Total thickness	Thousand grain weight	Expansion rate ± SD	Crack rate ± SD
GZ243	27.82	13.03	40.85	16.57	115.18% ± 9.88%	83.00% ± 6.67%
GZ060	36.49	14.25	50.74	20.21	114.78% ± 4.94%	81.00% ± 3.33%
GZ241	45.89	12.64	58.52	22.90	111.43% ± 5.37%	90.00% ± 0%
GZ136	48.20	17.37	65.57	23.81	91.67% ± 10.02%	77.50% ± 2.33%
GZ162	50.05	18.87	68.91	25.77	138.89% ± 7.84%	96.50% ± 0%
GZ093	50.20	4.45	54.64	29.13	117.1% ± 7.26%	73.50% ± 4.43%
GZ150	56.84	29.28	86.12	30.62	92.11% ± 2.34%	9.00% ± 0.67%
GZ210	72.35	7.75	80.10	19.18	111.43% ± 2.61%	81.50% ± 1.67%
GZ234	76.05	16.47	92.52	21.86	102% ± 2.61%	89.50% ± 2.33%
GZ202	84.60	23.13	107.73	16.23	126.87% ± 10.75%	96.50% ± 2.33%
GZ187	88.81	17.53	106.34	22.29	169.44% ± 5.74%	99.00% ± 0.67%
GZ192	88.90	14.59	103.49	17.46	169.09% ± 9.30%	86.50% ± 4.33%
GZ104	97.01	16.44	113.45	28.63	146.02% ± 1.16%	93.00% ± 0%
GZ220	99.47	16.32	115.78	26.26	133.33% ± 0%	98.00% ± 3.33%
GZ061	135.85	34.45	170.30	21.81	190.71% ± 10.02%	100.00% ± 0%
GZ123	157.34	0.00	157.34	26.56	151.08% ± 5.16%	94.50% ± 1.00%

## Discussion

### The Origin of Chinese Sorghum

A comprehensive understanding of the origin and domestication history of crops will help us better manage and use it. In this study, we conducted substantial investigations on population genomics and evolutionary history of Chinese sorghum based on resequencing of 333 diverse accessions. Our population genetics analyses revealed that sorghum accessions from South China were more closely related to wild relatives and landraces/varieties from African and South Asian. Similar patterns were observed in Chinese castor originated in Africa (Fan et al., 2019) and Chinese cotton originated in India (Du et al., 2018), Southern varieties are more closely related to wild relatives than Northern varieties. As the local residents in the southwestern border of China have historically maintained close contact with the people of the Indochina Peninsula and the Indian subcontinent (You, 1989). This is also the origin of the ancient Chinese name of sorghum, “ShuShu,” which means millet-like crop grown in the Kingdom of Shu, located in the southwestern region of China (Zhao, 2019a). We hence believe that sorghum may have been introduced from India to the southwestern region of China and subsequently spread into middle and northern China, supporting the hypothesis that sorghum entered China through the Southwest Silk Road (Zhao et al., 2019). Due to the limited sampling size (38 African and 21 Asian cultivated accessions) used in this study, this conclusion is considered tentative and needs further validation with larger sample sizes.

### Linkage Disequilibrium and Selective Sweep on Sorghum Collections

Sorghum, as an often cross-pollinated crop, has a lower linkage disequilibrium (LD) decay rate than self-pollinated rice (120 kb and 160 kb for indica and japonica, respectively) (Xu et al., 2011), and much higher than open-pollinated maize (∼30 kb) (Hufford et al., 2012). The LD from wild sorghum to Chinese cultivated accession extended to background level from ∼50 to ∼ 200 kb in our collection. This is consistent with LD estimates in sorghum by using GBS and WGS techniques (Mace et al., 2013; Morris et al., 2013). In addition, chromosomes with the slowest decay rate were different in different Chinese sorghum populations, and similar phenomenon was also observed in bread wheat (Hao et al., 2020). The possible reasons are that local agroecological environments and human preference, or some genes for important agronomic traits clustering in the same segment, which causes each chromosome to experience different selection pressures during domestication in the different populations.

Our research also revealed that more genes have been subject to domestication between southern and northern populations than southern and Chishui populations, suggesting that the number of selective sweep signals varies between different populations. Likewise, around half of the important genes is related to biotic and abiotic stress resistance, while approximately 30% orthologous genes are associated with yield, quality, and plant height. This confirms that crop domestication is the result of a synergistic effect of natural and artificial selection (Smith et al., 2019).

### Domestication of Sorghum Grain Physical Properties Related to Liquor Brewing Process

The Southwest region in China, especially in the Chishui River Basin between Guizhou and Sichuan province, is the birthplace and main producing area of Maotai-aroma liquor. It is generally believed that the glutinous sorghum with features of small grain and thick hull can withstand 30 processes and several rounds of steaming and boiling in brewing Maoutai’s. Phenotype data showed Chishui and Southern sorghums have smaller grains than Northern sorghum, and the GWAS identified two important domestication genes Sobic.009G124200 and Sobic.010G111200 for TGW and GW. The two genes are an ortholog of the *SMALL ORGAN SIZE1* (*SMOS1*) and *OsGASR7/GW6* controlling grain size in rice (Aya et al., 2014; Shi et al., 2020). The varieties possessing longer and looser panicles with pendulous branches are more suitable for the hot and humid climate in southern China in summer due to their resistance to mold and insects at the stage of maturity, whereas sorghum panicle length is negatively correlated with grain size (Tao et al., 2021), indicating that natural selection for panicle length genes in southern and Chishui populations is likely to cause selective sweep of genes controlling grain size.

Grain pericarp thickness failed to catch research’s attention, probably because they have no obvious relationship with traits such as yield, quality, and resistance to biotic and abiotic stresses. The *Z* gene has not been extensively studied since it was firstly reported in 1930s (Ayyangar and Ayyar, 1938), which is often used as a morphological marker to construct genetic maps in sorghum (Tao et al., 1998; Boivin et al., 1999; Mace et al., 2019). Varieties with light-colored grains are easy to determine whether pericarp is pearly, but those with dark-colored grains are difficult to determine whether pericarp is pearly, which might lead to an inaccurate genetic mapping of *Z* gene on chromosome. Based on the observation under a digital microscope, our GWAS analysis identified an important loci of pericarp thickness that is ∼2 Mb away from the previously reported *Z* gene locus (Mace et al., 2019). The possible candidate gene Sobic.002G228600 is an ortholog of *GRMZM5G898880* involving in striga resistance in maize (Adewale et al., 2020), and a QTL for striga resistance was reported in nearby areas (60.58–60.80 Mb) in a previous sorghum study (Haussmann et al., 2004). Sobic.002G228600 gene showed an insertion and deletion of “CATATTACAATCC” in the 5′′-UTR in accessions with thin and thick pericarp. 5′-UTR contains key elements of translational regulation, such as structural motifs and upstream open reading frames (uORFs). By controlling the selection of translation initiation sites (TISs), many sequence elements in 5′-UTR contribute to mRNA translatability (Hinnebusch et al., 2016). For example, the significant InDels in the 5′-UTR alter the expression of *BRB* (Big Root Biomass) in sesame (Dossa et al., 2021), and an 8-bp InDel in the 5′-UTR of *SlbHLH59* regulates ascorbate biosynthesis in tomato (Ye et al., 2019).

We found that *Z* gene is located in a ∼140 kb (61.96–62.10 Mb) regions on chromosome 2 that underwent the strong artificial selection in Chishui population. In addition to grain pericarp thickness, this significant region is likely to be related to grain size, panicle shape, stem morphology and disease resistance. Several genes in the vicinity of *Z* gene locus, such as Sobic.002G228800, Sobic.002G228900, Sobic.002G229000, and Sobic.002G229100, are homologous to the genes controlling panicle architecture and seed size in Arabidopsis, rice, and maize. The gene Sobic.002G228700 encodes an ortholog of *PATATIN-RELATED PHOSPHOLIPASE A* (*DEP3*), which affects the architecture of inflorescence in rice (Qiao et al., 2011), as well as the cellulose content and cell elongation in Arabidopsis (Li M. et al., 2011). The gene Sobic.002G228800 encodes an ortholog of *C-TERMINALLY ENCODED PEPTIDE* (*OsCEP6.1*) in rice, which regulates the development of panicle type and grain size by changing cell size without altering cell number (Sui et al., 2016). Two genes (Sobic.002G229000 and Sobic.002G229100) encode not only an ortholog of a *PUTATIVE SERINE CARBOXYPEPTIDASE* (*GS5*) as a positive regulator of rice grain size (Li Y. et al., 2011), but also an ortholog of *SERINE CARBOXYPEPTIDASE-LIKE* 40 (GRMZM2G072240) involving in formation of the number of hull layers in maize (Cui et al., 2018). Several QTLs for panicle shape, thousand grain weight, and grain yield were consistently mapped in the same genomic regions as in the previous studies in sorghum (Mantilla Perez et al., 2014; Zhou et al., 2019). Moreover, many QTLs related to disease resistance and morphology were also reported in this genomic region, such as shoot fly resistance (Aruna et al., 2011), ergot resistance (Parh et al., 2008), downy mildew resistance (Ahn et al., 2019), rust resistance (Wang et al., 2014), plant height (Shiringani et al., 2010; Mantilla Perez et al., 2014), tillering number (Alam et al., 2014; Kong et al., 2015), polyphenol content (Rhodes et al., 2017). This is in line with the main traits of Chishui sorghum varieties, which possess smaller grain size, looser panicle, better disease resistance and higher plant height, to adapt to local agroecological environments, such as hot and humid summer, serious pests and diseases. Compared with the thickness of pericarp, it is easier for local farmers to select new varieties based on traits such as grain size, panicle shape, and disease resistance during the domestication and improvement of sorghum. As a result, the strong artificial selection of grain size, panicle type or resistance to biotic stress is most likely to cause the selective sweep, which led to a genetic hitchhiking of nearby *Z* gene conferring pericarp thickness and a large reduction of the genetic diversity in the 140 kb regions on chromosome 2.

### Relationship Between Baijiu-Brewing Process and the Physical Properties of Grains

Traditionally, Moutai-aroma liquor brewing has always preferred glutinous sorghum varieties with smaller size and thicker pericarp, which were thought to meet the process technology of repeated steaming (Chen and Ji, 2006). Contrary to the conventional belief, our results revealed that the increase in pericarp thickness results in larger grain size, and that pericarp thickness rather than grain size is associated with the rate of expansion and crack in waxy sorghum, meaning that smaller-size grains have thinner pericarp, while grain with thinner pericarp generally exhibit better resistance to cooking. The similar results were reported in the effect of grain physical properties on sorghum processing. A thick pericarp absorbs more water and is more easily detached during the dehulling than a thin pericarp (Scheuring et al., 1983). Our findings also suggest that selection for *Z* gene should be avoided in sorghum high-yield breeding program, because the high-yielding varieties caused by thick pericarp have lower rate of dehulled grains and flour yield during milling (Lu, 1999).

In summary, population structure analysis revealed that 333 sorghum accessions clustered into six groups of wild relatives, cultivated sorghum in Africa, Southern Asia, Southern China, Northern China, and Chishui in Southwest China, supporting the hypothesis that sorghum entered China through the Southwest Silk Road. We discovered a significant selective sweep that harbors several genes for pericarp thickness, grain size, panicle type or biotic stress tolerance, which resulted in a large reduction of the genetic diversity in the 140 kb regions on chromosome 2. Based on the cooking experiment, we put forward a more scientific standard for sorghum breeding that is suitable for liquor brewing technology in China. Our results provided useful information for the breeding and improvement not only for liquor-sorghum but also for other breeding programs in China.

## Data Availability Statement

The datasets presented in this study can be found in online repositories. The name of the repository and accession number can be found below: China National GeneBank (CNGB); CNP0002968.

## Author Contributions

LZ and GZ designed and managed the research and wrote the manuscript. YD, JX, XG, KL, ZF, NC, BC, LBZ, MR, and ZX collected the plant materials, investigated traits, and performed the experiments. YD, XL, and ZB analyzed whole-genome sequence data and RNA-seq data. ZX and YT revised the manuscript. All authors read and approved the final manuscript.

## Conflict of Interest

ZB was employed by Shanghai OE Biotech Co., Ltd., China. The remaining authors declare that the research was conducted in the absence of any commercial or financial relationships that could be construed as a potential conflict of interest.

## Publisher’s Note

All claims expressed in this article are solely those of the authors and do not necessarily represent those of their affiliated organizations, or those of the publisher, the editors and the reviewers. Any product that may be evaluated in this article, or claim that may be made by its manufacturer, is not guaranteed or endorsed by the publisher.

## Footnotes

1
http://bigd.big.ac.cn/sorgsd

2
https://genome.jgi.doe.gov/portal/pages/dynamicOrganismDownload.jsf?organism=Phytozome

3
http://broadinstitute.github.io/picard/

4
http://evolution.genetics.washington.edu/phylip.html

5
https://github.com/BGI-shenzhen/PopLDdecay

6
https://www.maizegenetics.net/gapit

7
http://web.expasy.org/translate/

8
https://phytozome.jgi.doe.gov
