# Supplementary material for: Selection Signatures in Chinese Sorghum Reveals Its Unique Liquor-Making Properties
Source: Front Plant Sci. 2022 Jun 9;13:923734. doi: 10.3389/fpls.2022.923734 (PMC9218943; doi:10.3389/fpls.2022.923734)
Supplement: Supplementary file 1 [file Data_Sheet_1.docx]

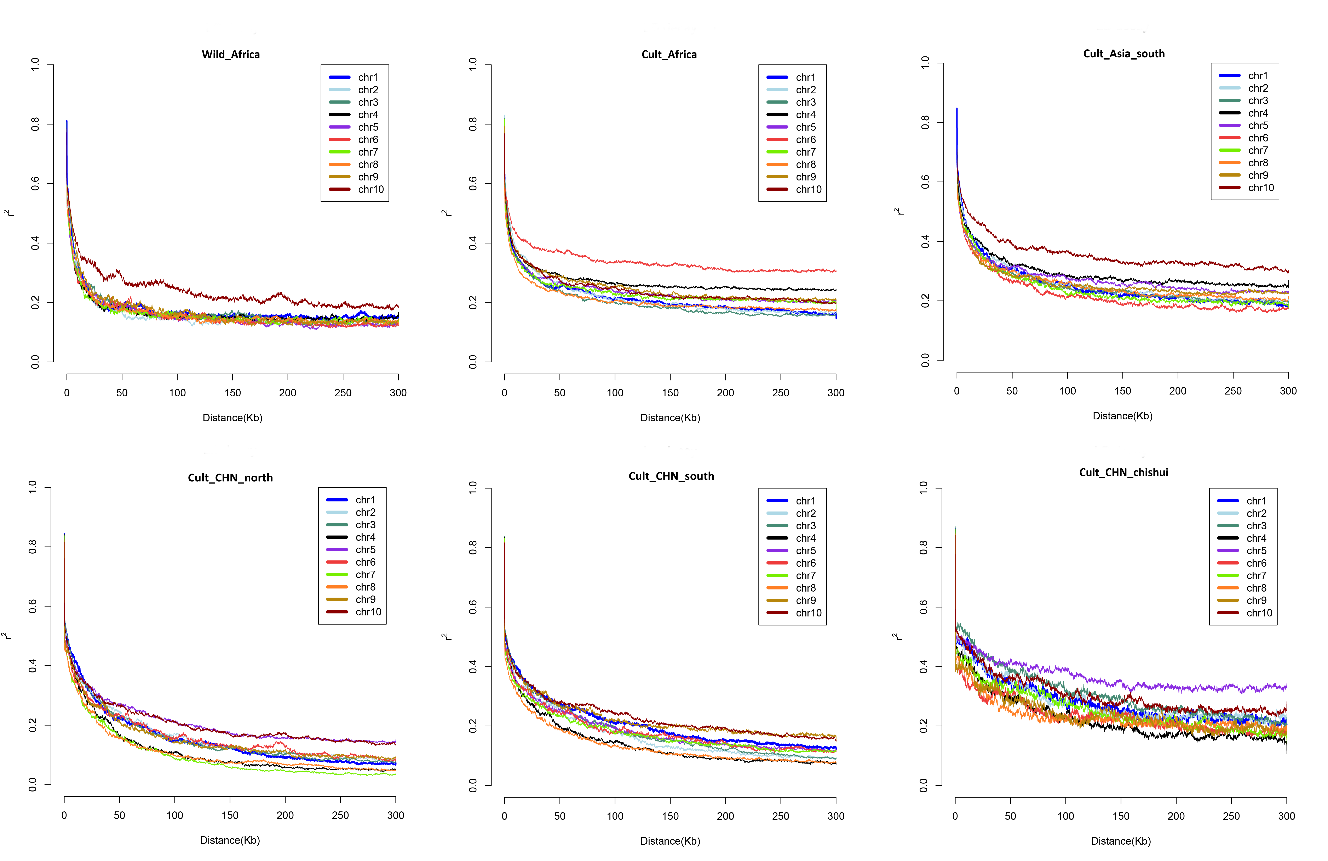


**Supplemental Figure 1** LD decay of ten chromosomes for the six populations.


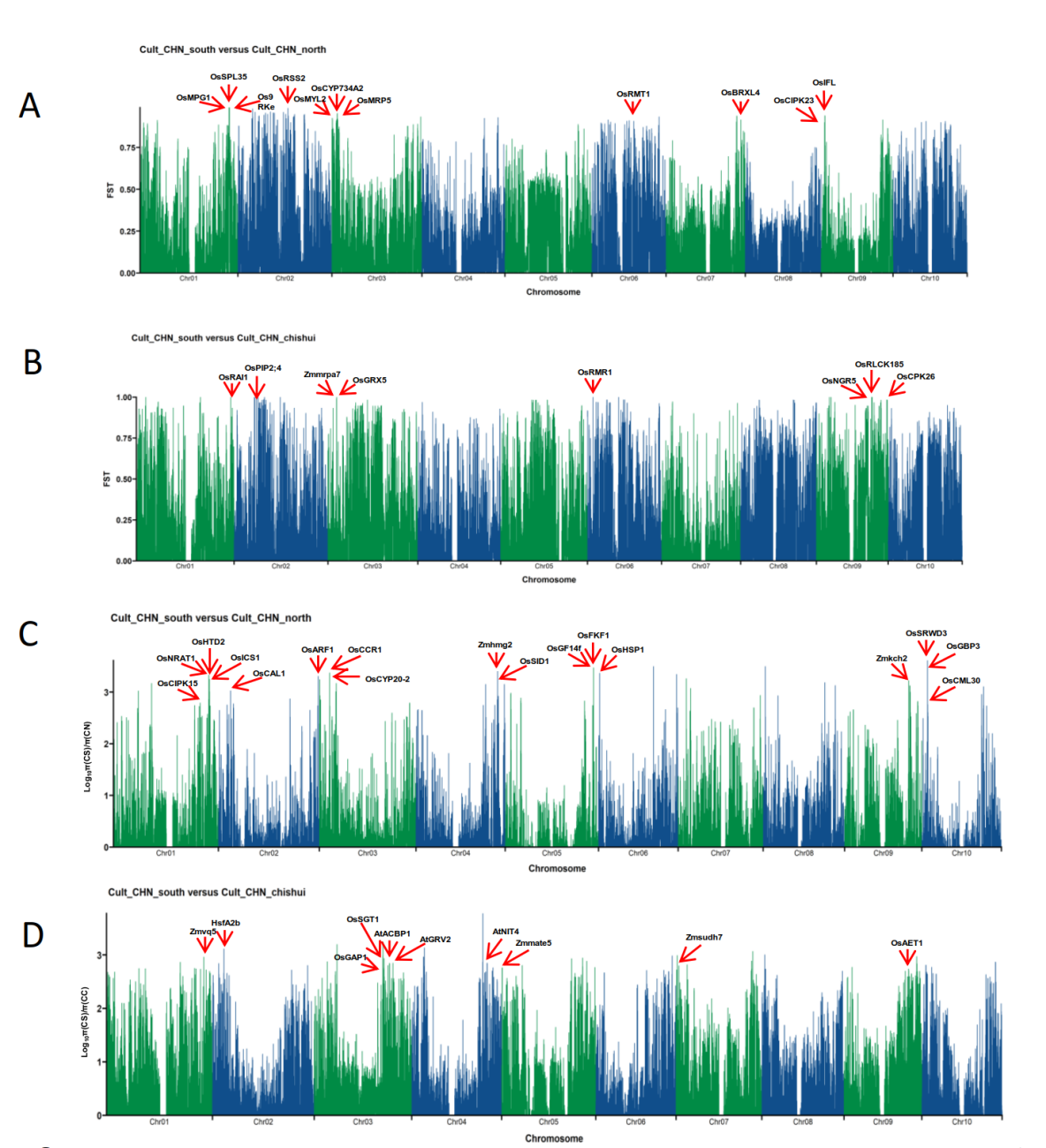


**Supplemental Figure 2** The Selective sweep regions identified by the greatest relative divergence (*Fst*) between South vs North population (**A**) and between South vs Chishui population (**B**), and by the nucleotide diversity (*π* ratio) between South vs North population (**C**) and between South vs Chishui population (**D**) in China. The red arrows point to the strongest selective sweep signals (the top 0.1% of the domestication genes) and the corresponding orthologue genes in rice, maize, or Arabidopsis.


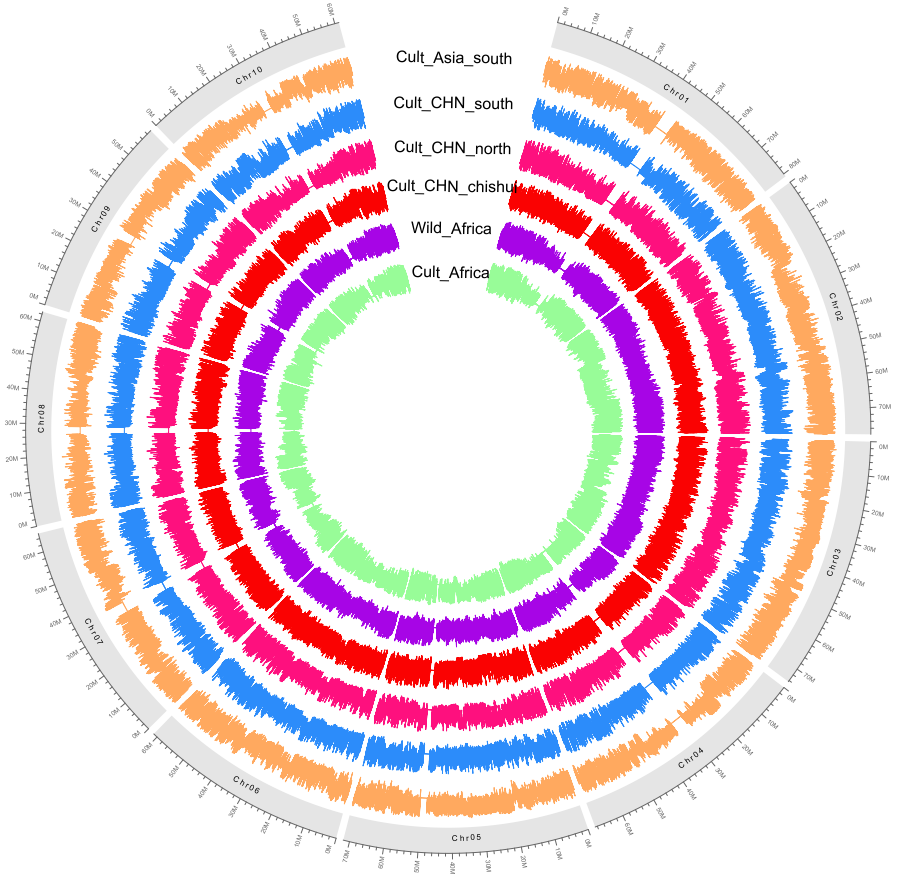


**Supplemental Figure 3** The selective sweeps identified by the Tajima’D for each chromosome among six populations.


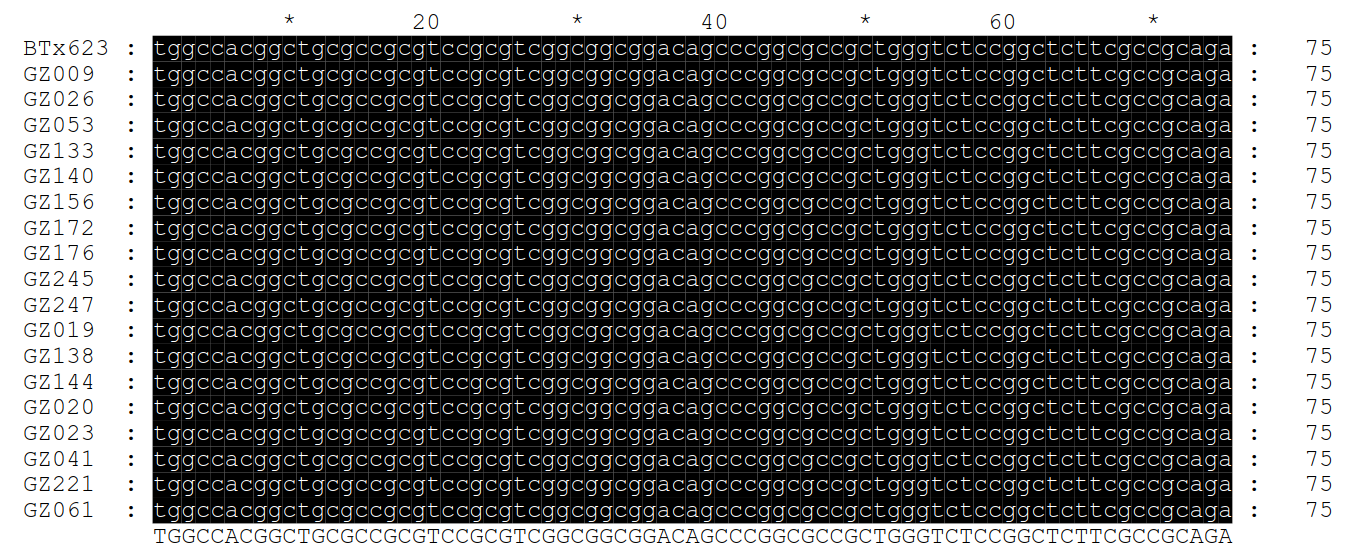

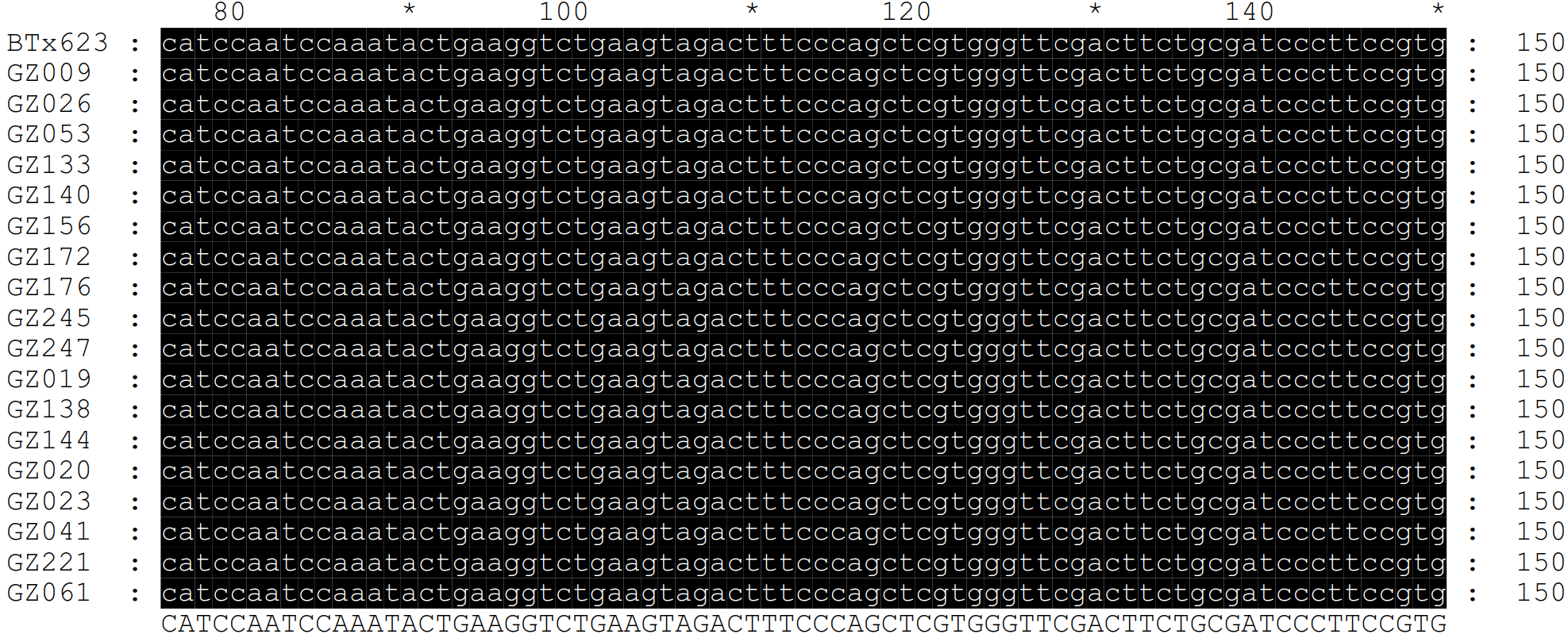

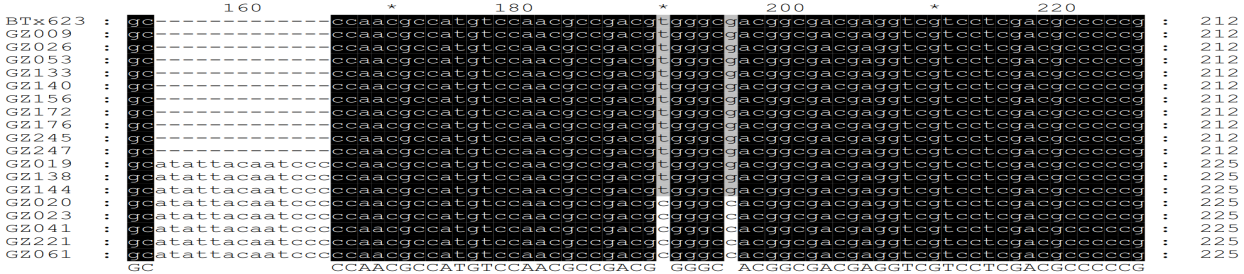

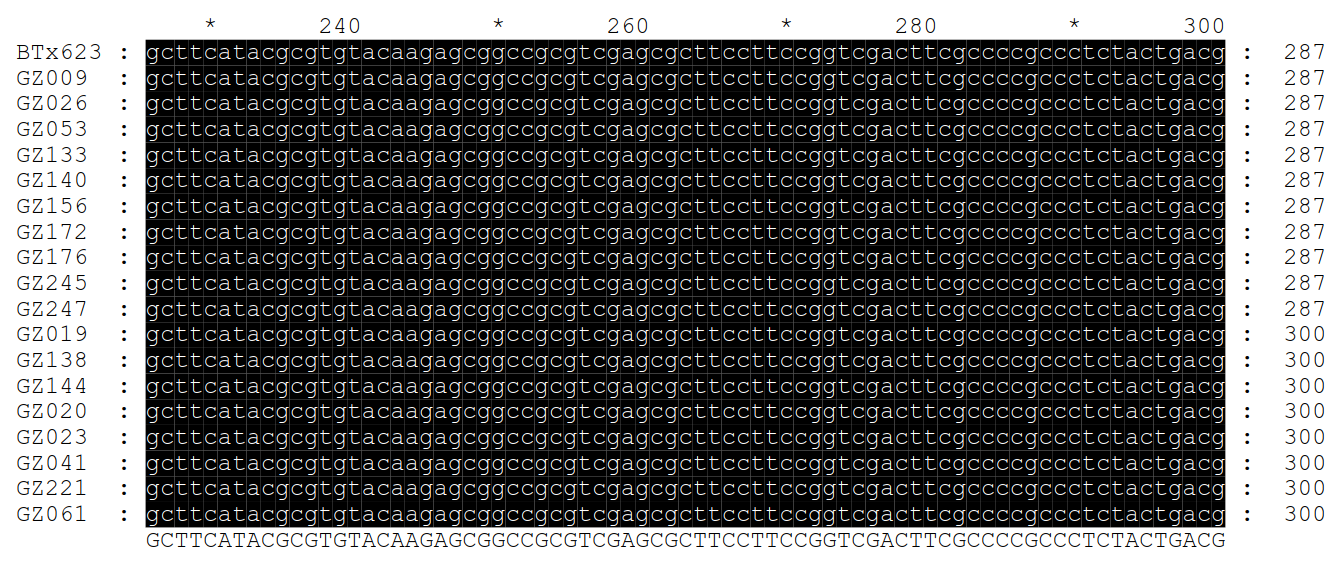

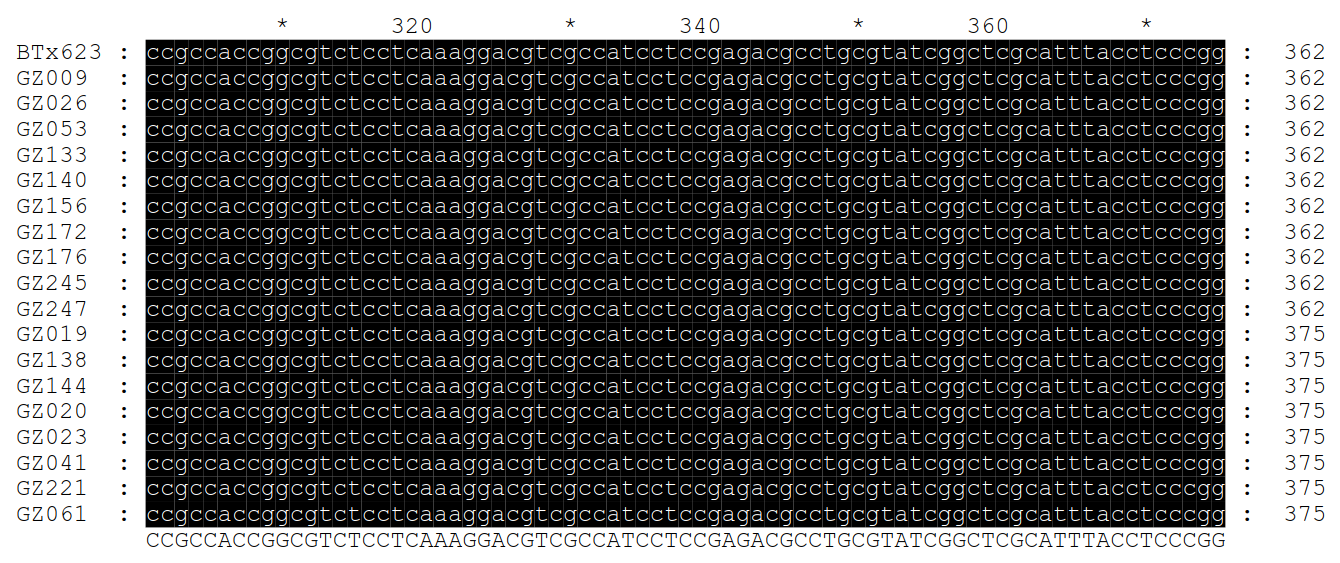

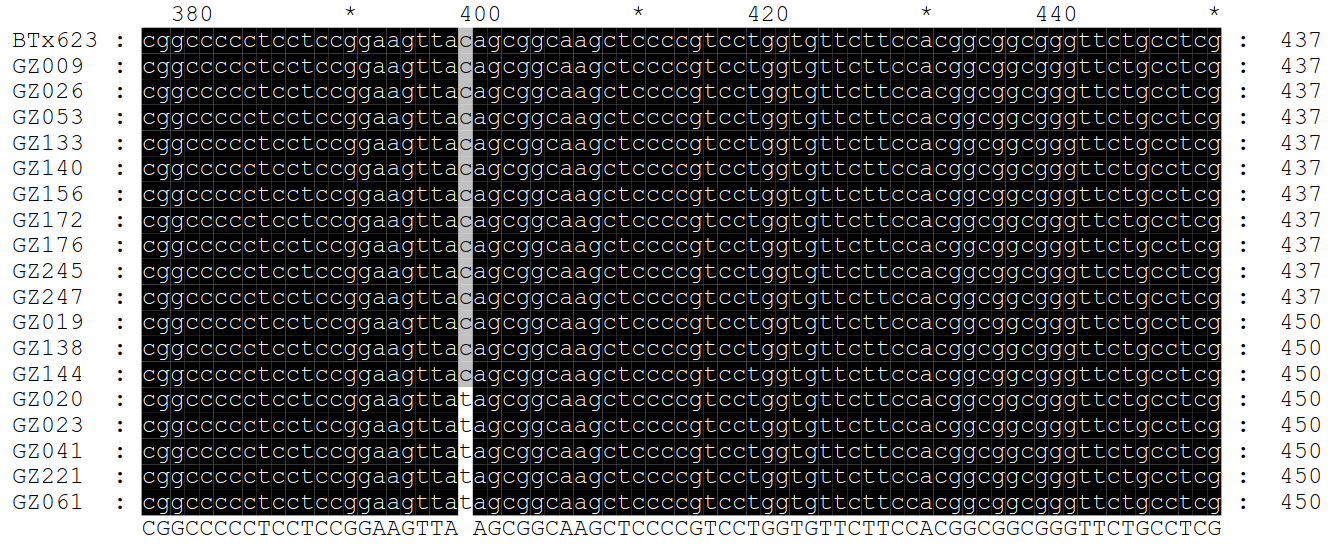

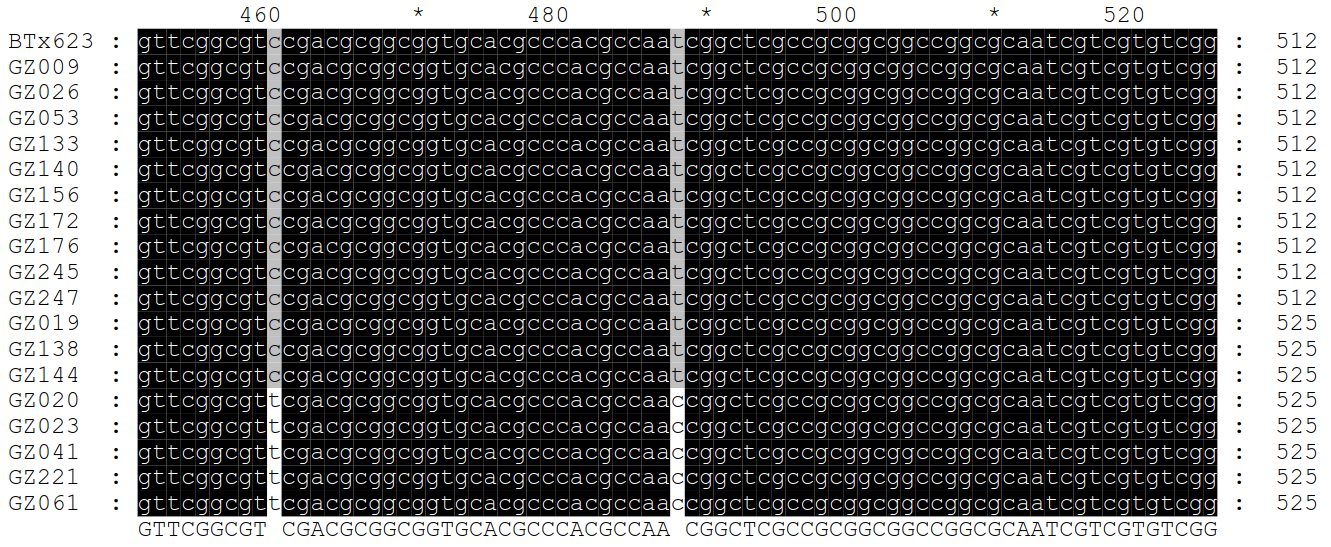

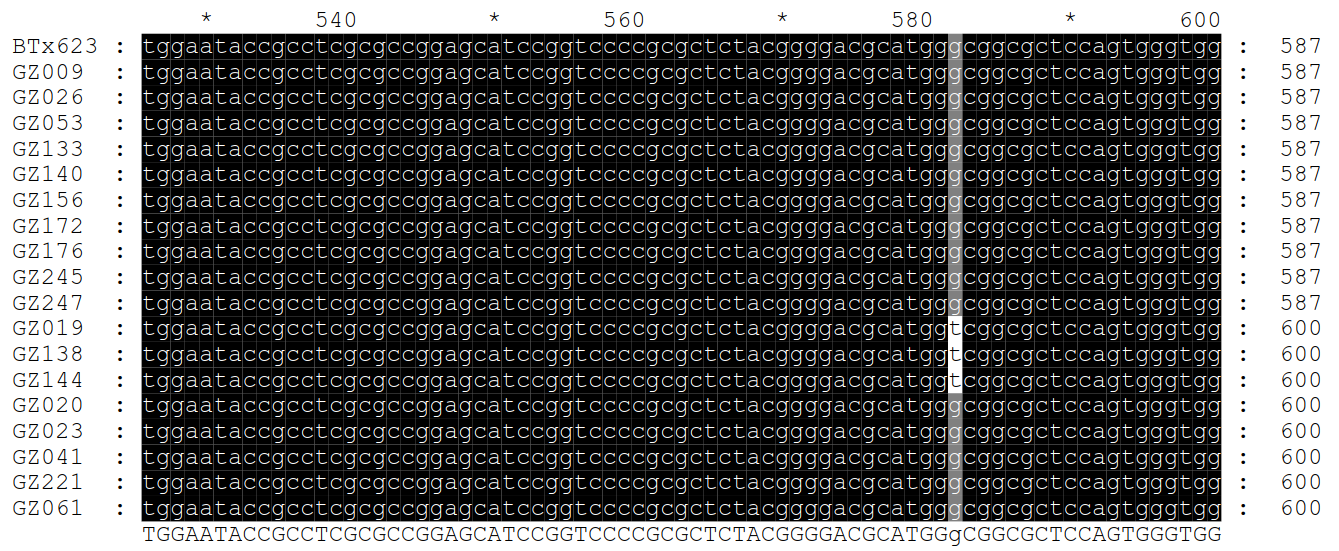

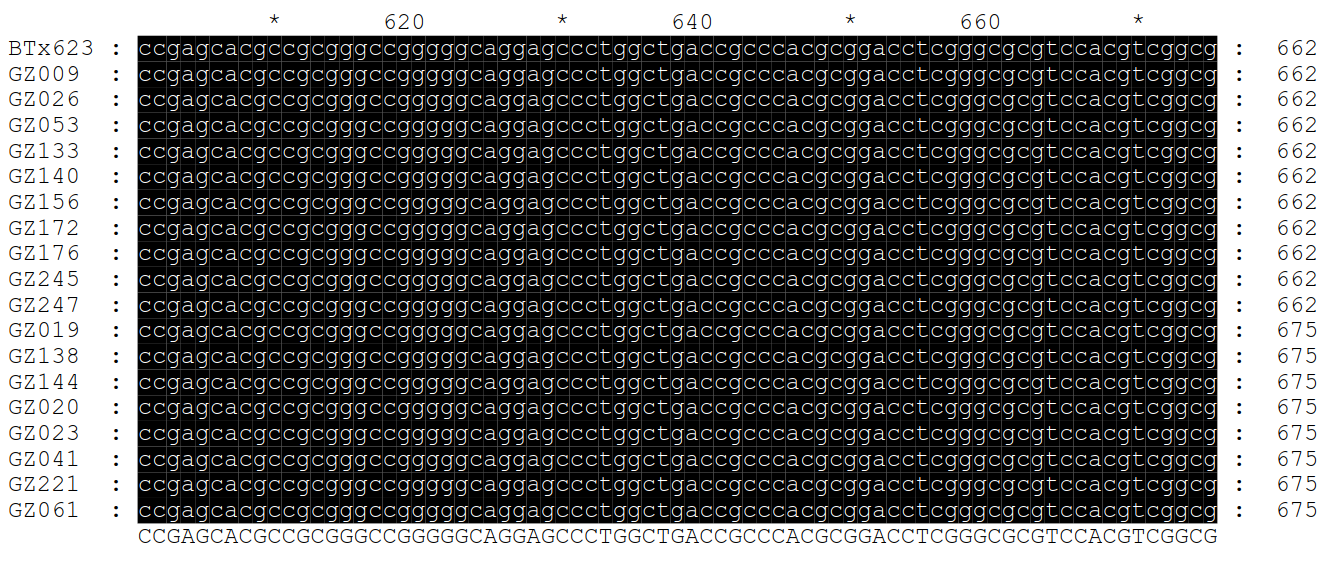

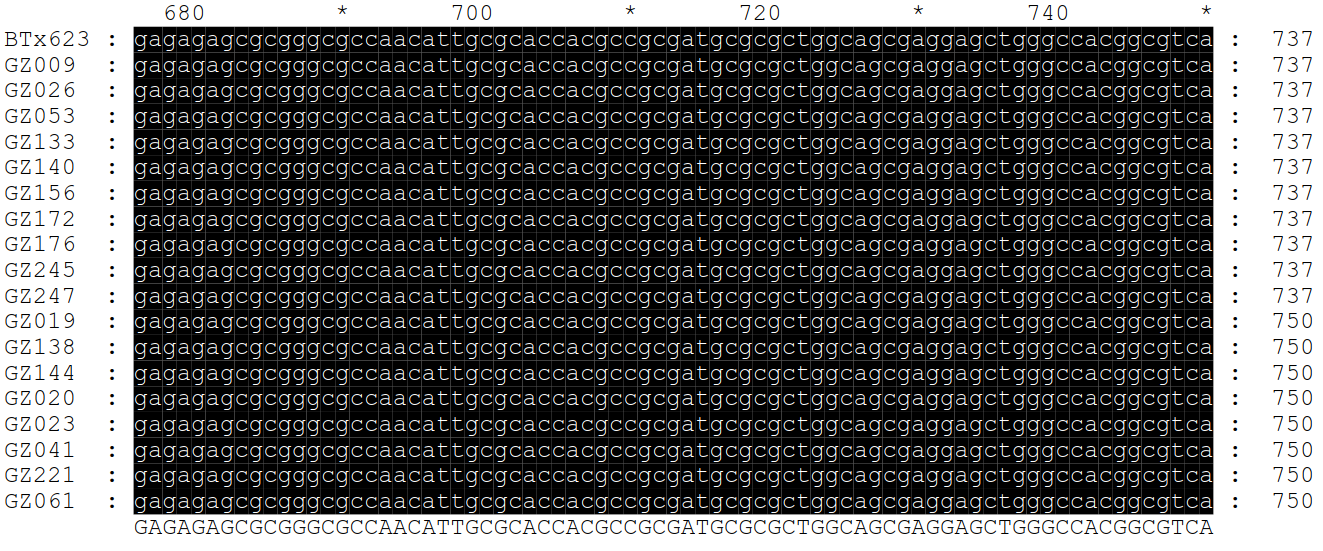

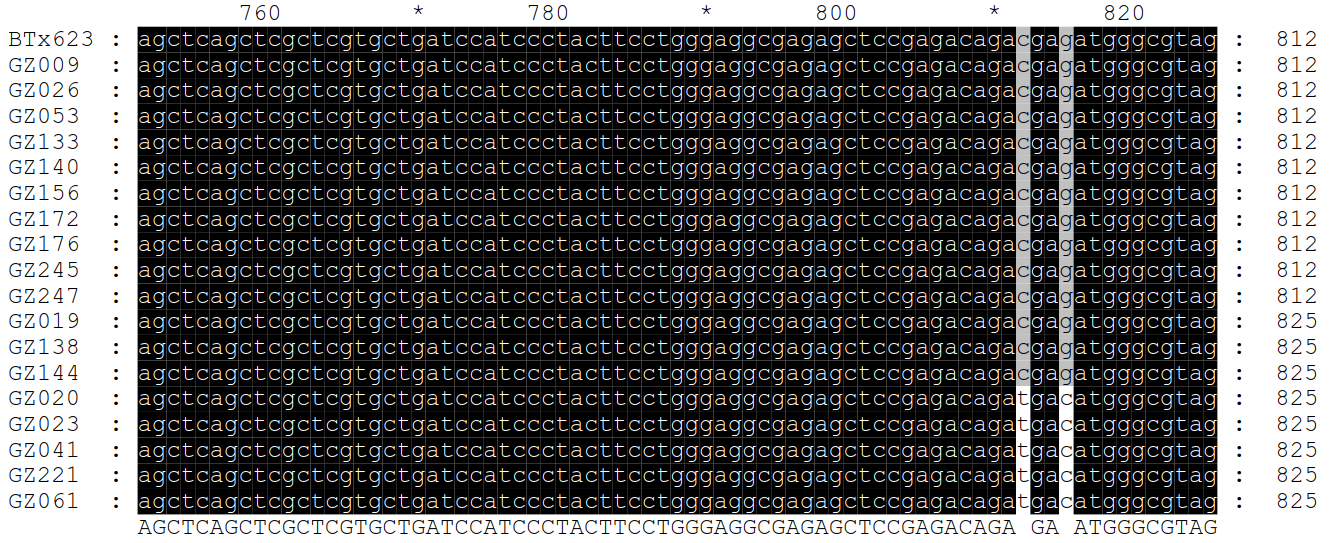

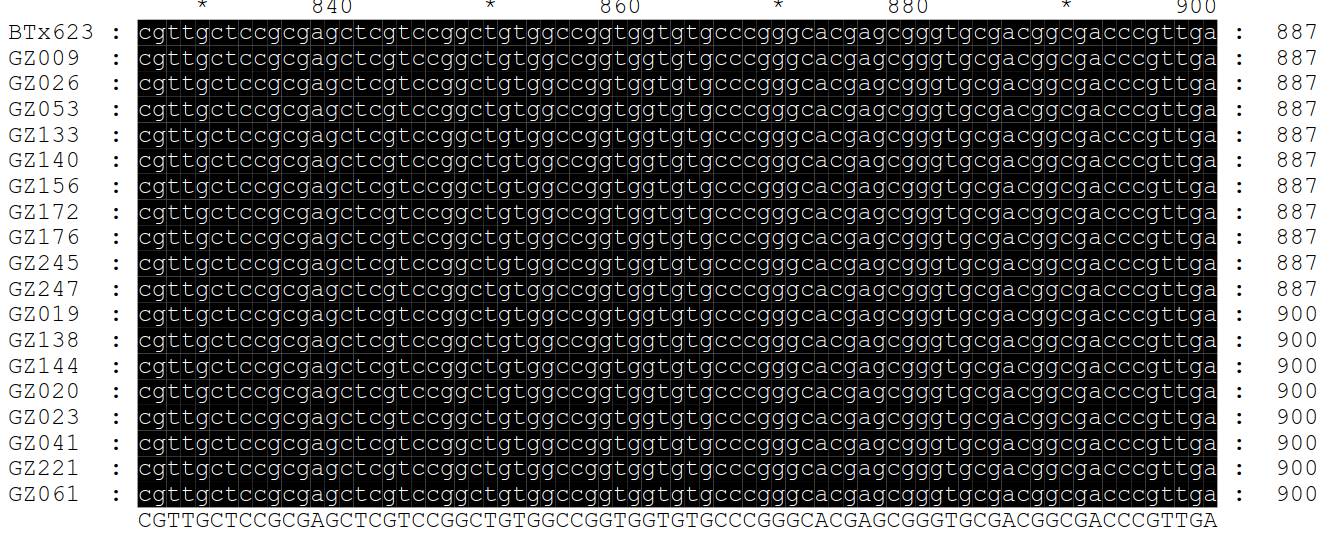

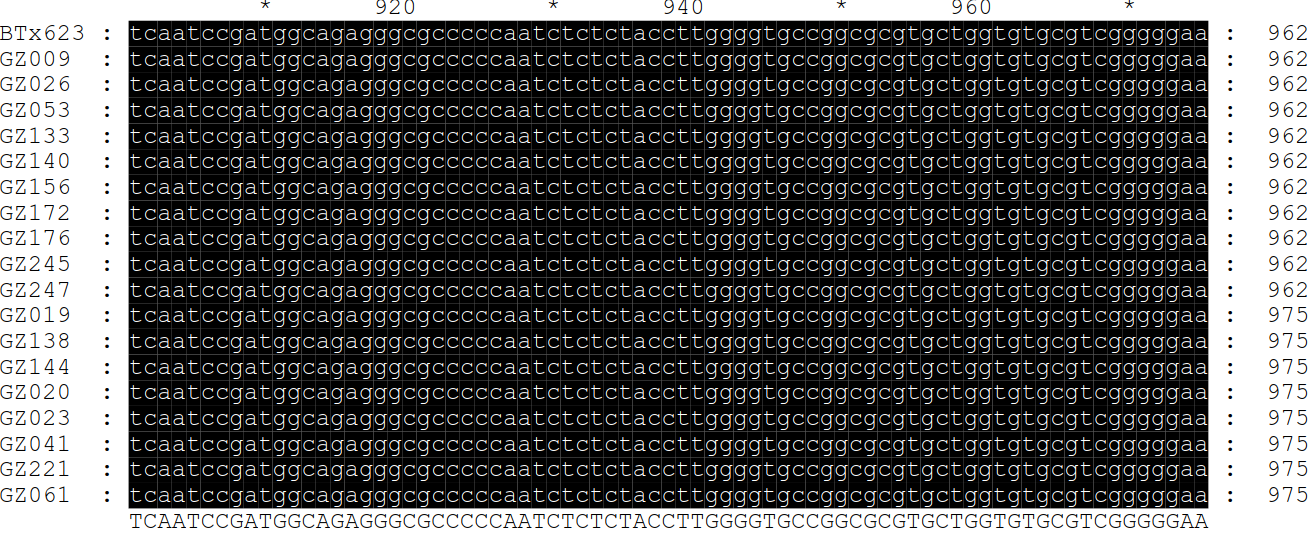

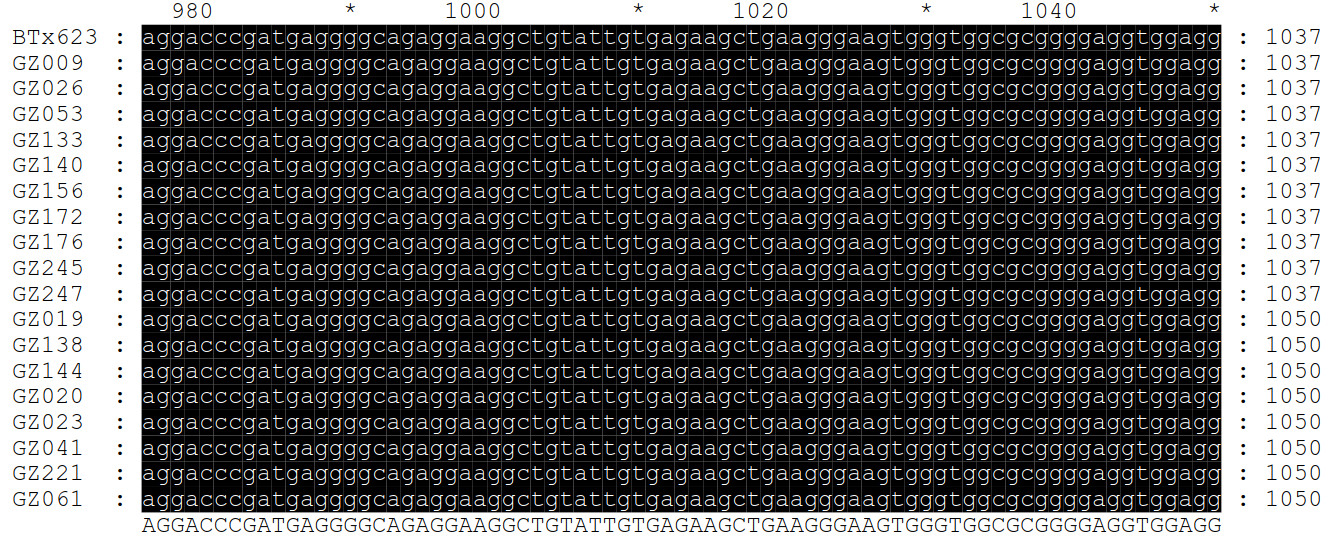

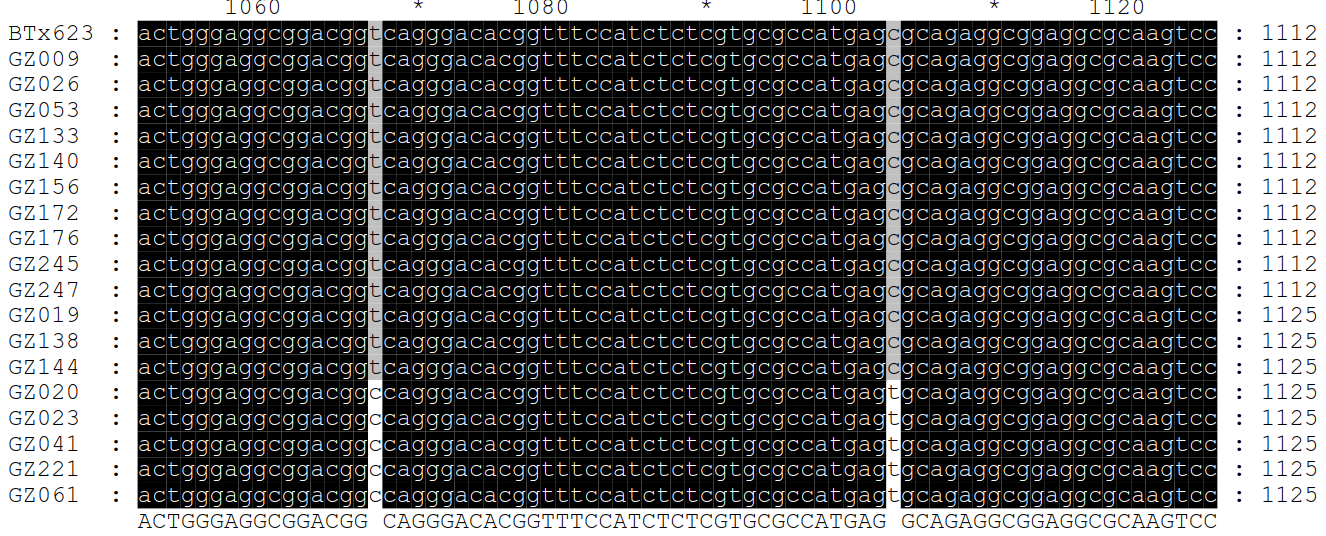

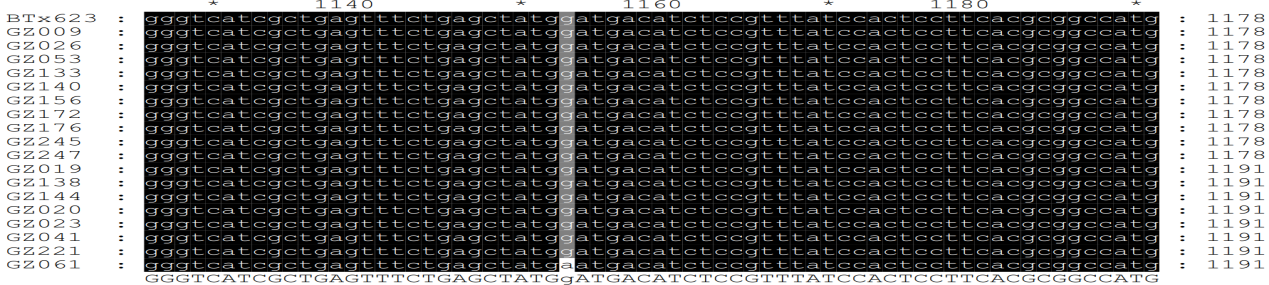


**Forward primer2**

**Forward primer1**

**Reverse primer1**

**Reverse primer2**

**Supplemental Figure 4** Sequence alignment of candidate gene (Sobic.002G228600) of extreme phenotypic varieties for the thickness of mesocarp. The full length of Sobic.002G228600 is 1202 bp, and the CDS length is 984bp. The positions of the start (red box) and stop (green box) codons are located at 163bp and 1146bp, respectively. The positions of two primers are marked. BTx623, GZ009, GZ133, GZ140, GZ156, GZ172, GZ176, GZ245, GZ247, and GZ138 are varieties with thinner pericarp, while GZ053, GZ026, GZ019, GZ144, GZ020, GZ023, GZ221, GZ041, and GZ061 are varieties with thicker pericarp.
