# Supplementary material for: Selection Signatures in Chinese Sorghum Reveals Its Unique Liquor-Making Properties
Source: Front Plant Sci. 2022 Jun 9;13:923734. doi: 10.3389/fpls.2022.923734 (PMC9218943; doi:10.3389/fpls.2022.923734)
Supplement: Supplementary file 2 [file Data_Sheet_2.docx]

**Supplemental Table 1** Information of 333 Sorghum accessions, and assigned groups at K=10 based on STRUCTURE analysis

| **Sample ID** | **Plant Name** | **Origin country** | **Origin region** | **Type** | **Source** | **Assigned Clusters** |
| --- | --- | --- | --- | --- | --- | --- |
| 66 | JUAR - India | India | India | Grain | Zhang LM_PC | Cult_South_Asia |
| 123 | ETS 3889 - Ethiopia | Ethiopia | Ethiopia | Grain | Zhang LM_PC | Cult_Africa |
| 260 | C048 | Cameroon | Cameroon | Wild | Zhang LM_PC |  |
| 348 | 0 | Egypt | Egypt | Wild | Zhang LM_PC |  |
| 353 | Diri | Sudan | Sudan | Wild | Zhang LM_PC |  |
| 355 | Juar | India | India | Wild | Zhang LM_PC |  |
| 357 | 1666 | South Africa | South Africa | Wild | Zhang LM_PC |  |
| 364 | O-1008 | Ethiopia | Ethiopia | Wild | Zhang LM_PC | Wild_Africa |
| 367 | Dhurra | Oman | Oman | Grain | Zhang LM_PC |  |
| 370 | Dhurra | Oman | Oman | Grain | Zhang LM_PC |  |
| 377 | SA 1527 | South Africa | South Africa | Wild | Zhang LM_PC |  |
| 392 | A-7171 | Former Soviet Union | Former Soviet Union | Wild | Zhang LM_PC |  |
| 2009 | FETERITA FAYOUMI-6 | Sudan | Sudan | Grain | Zhang LM_PC | Cult_Africa |
| 2010 | FETERITA NIDIANA | Sudan | Sudan | Grain | Zhang LM_PC |  |
| 2030 | MN-832 (Kododok) | Sudan | Sudan | Grain | Zhang LM_PC | Cult_Africa |
| 2031 | MN-1124 (Potch 8) | Sudan | Sudan | Grain | Zhang LM_PC | Cult_Africa |
| 302546 | *Sorghum propinquum* | NA | NA | *Sorghum propinquum* | Zhang LM_PC | Wild_Africa |
| GW102 | WHITE GOVINDGARH | Sudan | Sudan | Grain | Zhang LM_PC |  |
| GW104 | PURDUE NO 49072 | Cameroon | Cameroon | Grain | Zhang LM_PC |  |
| GW16 | 694 | Yemen | Yemen | Grain | Zhang LM_PC | Cult_South_Asia |
| GW2 | R2-38 | Eritrea | Eritrea | Grain | Zhang LM_PC |  |
| GW34 | 821 | Yemen | Yemen | Grain | Zhang LM_PC | Cult_South_Asia |
| GW58 | AP79-226 | Cameroon | Cameroon | Grain | Zhang LM_PC |  |
| GW59 | DhiraH "amra, Dhira Safra,  Dhirah Selmi" | Yemen | Yemen | Grain | Zhang LM_PC | Cult_South_Asia |
| GW9 | 548 | Yemen | Yemen | Grain | Zhang LM_PC | Cult_South_Asia |
| GZ1 | 253 | China | Liaoning | Grain | This study | Cult_CHN_North |
| GZ10 | Baiyunfan | China | Unknown | Grain | This study | Cult_CHN_North |
| GZ100 | 1131 | China | Hebei | Grain | This study | Cult_CHN_North |
| GZ101 | 417 | China | Hebei | Grain | This study | Cult_CHN_North |
| GZ102 | Zhong733 | China | Hebei | Grain | This study | Cult_CHN_North |
| GZ103 | 367 | China | Hebei | Grain | This study | Cult_CHN_North |
| GZ104 | 1135 | China | Hebei | Grain | This study | Cult_CHN_North |
| GZ105 | 1134 | China | Hebei | Grain | This study | Cult_CHN_North |
| GZ106 | 566 | China | Shanxi | Grain | This study | Cult_CHN_North |
| GZ107 | 328 | China | Shanxi | Grain | This study | Cult_CHN_North |
| GZ108 | 285 | China | Shanxi | Grain | This study | Cult_CHN_North |
| GZ109 | 702 | China | Shanxi | Grain | This study | Cult_CHN_North |
| GZ11 | Banjiang Sorghum-Gx | China | Guangxi | Grain | This study |  |
| GZ110 | 0390 | China | Inner Mongolia | Grain | This study | Cult_CHN_North |
| GZ111 | Yong 123 | China | Inner Mongolia | Grain | This study | Cult_CHN_North |
| GZ112 | 0242 | China | Inner Mongolia | Grain | This study | Cult_CHN_North |
| GZ113 | 0371 | China | Inner Mongolia | Grain | This study | Cult_CHN_North |
| GZ114 | 0524 | China | Inner Mongolia | Grain | This study | Cult_CHN_North |
| GZ115 | 0433 | China | Inner Mongolia | Grain | This study | Cult_CHN_North |
| GZ116 | 0686 | China | Inner Mongolia | Grain | This study | Cult_CHN_North |
| GZ117 | 0249 | China | Inner Mongolia | Grain | This study | Cult_CHN_North |
| GZ118 | 0254 | China | Inner Mongolia | Grain | This study | Cult_CHN_North |
| GZ119 | 1234 | China | Liaoning | Grain | This study | Cult_CHN_North |
| GZ12 | Beiping No. 5 | China | Beijing | Grain | This study | Cult_CHN_North |
| GZ120 | 241 | China | Liaoning | Grain | This study | Cult_CHN_North |
| GZ121 | 31 | China | Liaoning | Grain | This study | Cult_CHN_North |
| GZ122 | 1102 | China | Liaoning | Grain | This study | Cult_CHN_North |
| GZ123 | 240 | China | Liaoning | Grain | This study | Cult_CHN_North |
| GZ124 | 1044 | China | Liaoning | Grain | This study | Cult_CHN_North |
| GZ125 | 1030 | China | Liaoning | Grain | This study | Cult_CHN_North |
| GZ126 | 1029 | China | Liaoning | Grain | This study | Cult_CHN_North |
| GZ127 | 415 | China | Jiangsu | Grain | This study |  |
| GZ128 | Zhong211 | China | Anhui | Grain | This study |  |
| GZ129 | 221 | China | Shandong | Grain | This study | Cult_CHN_North |
| GZ13 | Local Soft Sorghum | China | Inner Mongolia | Grain | This study | Cult_CHN_North |
| GZ130 | 214 | China | Shandong | Grain | This study | Cult_CHN_North |
| GZ131 | 216 | China | Shandong | Grain | This study | Cult_CHN_North |
| GZ132 | 351 | China | Shandong | Grain | This study | Cult_CHN_North |
| GZ133 | 369 | China | Shandong | Grain | This study | Cult_CHN_North |
| GZ134 | 370 | China | Shandong | Grain | This study | Cult_CHN_North |
| GZ135 | Ⅱ657 | China | Shandong | Grain | This study | Cult_CHN_North |
| GZ136 | Ⅱ097 | China | Shandong | Grain | This study |  |
| GZ138 | 811333 | China | Hubei | Grain | This study |  |
| GZ139 | 811369 | China | Hubei | Grain | This study |  |
| GZ14 | Sticky Sorghum | China | Beijing | Grain | This study | Cult_CHN_North |
| GZ140 | 811424 | China | Hubei | Grain | This study |  |
| GZ141 | 811437 | China | Hubei | Grain | This study |  |
| GZ143 | 1081 | China | Shanxi | Grain | This study | Cult_CHN_North |
| GZ144 | 1181 | China | Shanxi | Grain | This study | Cult_CHN_North |
| GZ145 | 1155 | China | Shanxi | Grain | This study | Cult_CHN_North |
| GZ146 | 871255 | China | HeiLongjiang | Grain | This study | Cult_CHN_North |
| GZ147 | 871256 | China | HeiLongjiang | Grain | This study | Cult_CHN_North |
| GZ148 | 871258 | China | HeiLongjiang | Grain | This study | Cult_CHN_North |
| GZ149 | 871338 | China | HeiLongjiang | Grain | This study | Cult_CHN_North |
| GZ15 | Daluochui | China | Inner Mongolia | Grain | This study | Cult_CHN_North |
| GZ150 | Ⅲ072 | China | Shandong | Grain | This study | Cult_CHN_North |
| GZ151 | 82440 | China | Hubei | Grain | This study |  |
| GZ152 | 82256 | China | Hubei | Grain | This study | Cult_CHN_South |
| GZ153 | 82266 | China | Hubei | Grain | This study |  |
| GZ154 | 206 | China | Shaanxi | Grain | This study |  |
| GZ155 | 229 | China | Shaanxi | Grain | This study |  |
| GZ156 | 263 | China | Shaanxi | Grain | This study |  |
| GZ157 | 227 | China | Shaanxi | Grain | This study |  |
| GZ158 | 217 | China | Shaanxi | Grain | This study | Cult_CHN_South |
| GZ159 | 189 | China | Shaanxi | Grain | This study |  |
| GZ16 | Huangluoshan-Xs | China | Shanxi | Grain | This study | Cult_CHN_North |
| GZ160 | 222 | China | Shaanxi | Grain | This study | Cult_CHN_South |
| GZ161 | 235 | China | Shaanxi | Grain | This study |  |
| GZ162 | 257 | China | Shaanxi | Grain | This study | Cult_CHN_South |
| GZ163 | 249 | China | Shaanxi | Grain | This study | Cult_CHN_South |
| GZ164 | 279 | China | Shaanxi | Grain | This study |  |
| GZ165 | 291 | China | Shaanxi | Grain | This study | Cult_CHN_South |
| GZ166 | 273 | China | Shaanxi | Grain | This study |  |
| GZ167 | 84-300 | China | Shaanxi | Grain | This study | Cult_CHN_North |
| GZ168 | Gao01 | China | Yunnan | Grain | This study | Cult_CHN_South |
| GZ169 | R20 | China | Yunnan | Grain | This study | Cult_CHN_South |
| GZ17 | Daluochui | China | Unknown | Grain | This study | Cult_CHN_North |
| GZ170 | R62 | China | Yunnan | Grain | This study | Cult_CHN_South |
| GZ171 | R32 | China | Yunnan | Grain | This study | Cult_CHN_South |
| GZ172 | R23 | China | Yunnan | Grain | This study |  |
| GZ173 | R21 | China | Yunnan | Grain | This study | Cult_CHN_South |
| GZ174 | Gao14 | China | Yunnan | Grain | This study | Cult_CHN_South |
| GZ175 | R08 | China | Yunnan | Grain | This study | Cult_CHN_South |
| GZ176 | R09 | China | Yunnan | Grain | This study | Cult_CHN_South |
| GZ177 | Gao08 | China | Yunnan | Grain | This study | Cult_CHN_South |
| GZ178 | Gao10 | China | Yunnan | Grain | This study | Cult_CHN_South |
| GZ179 | Gao31 | China | Yunnan | Grain | This study | Cult_CHN_South |
| GZ18 | Dabaye | China | HeiLongjiang | Grain | This study | Cult_CHN_North |
| GZ180 | R01 | China | Yunnan | Grain | This study | Cult_CHN_South |
| GZ181 | R12 | China | Yunnan | Grain | This study | Cult_CHN_South |
| GZ182 | R18 | China | Yunnan | Grain | This study | Cult_CHN_South |
| GZ183 | R22 | China | Yunnan | Grain | This study | Cult_CHN_South |
| GZ184 | R34 | China | Yunnan | Grain | This study |  |
| GZ186 | Red Glutious-HB | China | Hubei | Grain | This study | Cult_CHN_South |
| GZ188 | Waxy Sorghum-HB | China | Hubei | Grain | This study | Cult_CHN_South |
| GZ189 | Shui512 | China | Sichuan | Grain | This study |  |
| GZ19 | Daguandong | China | Shanxi | Grain | This study | Cult_CHN_North |
| GZ190 | Shui421 | China | Sichuan | Grain | This study | Cult_CHN_South |
| GZ191 | Shui507 | China | Sichuan | Grain | This study |  |
| GZ192 | Yuanmou Red Yellow Waxy | China | Yunnan | Grain | This study |  |
| GZ193 | Lufeng Glutinous Sorghum | China | Yunnan | Grain | This study | Cult_CHN_South |
| GZ194 | Songming Glutinous Sorghum | China | Yunnan | Grain | This study |  |
| GZ195 | Shui723 | China | Sichuan | Grain | This study | Cult_CHN_South |
| GZ196 | Shui639 | China | Sichuan | Grain | This study |  |
| GZ197 | Shui712 | China | Sichuan | Grain | This study |  |
| GZ198 | Shui724 | China | Sichuan | Grain | This study | Cult_CHN_South |
| GZ199 | Q040 | China | Guizhou | Grain | This study | Cult_CHN_South |
| GZ2 | 427 | China | Liaoning | Grain | This study |  |
| GZ20 | Dahongke | China | HeiLongjiang | Grain | This study | Cult_CHN_North |
| GZ200 | Q047 | China | Guizhou | Grain | This study |  |
| GZ201 | 01511 | China | Guizhou | Grain | This study | Cult_CHN_South |
| GZ202 | 01522 | China | Guizhou | Grain | This study | Cult_CHN_South |
| GZ203 | 01529 | China | Guizhou | Grain | This study | Cult_CHN_South |
| GZ204 | 01535 | China | Guizhou | Grain | This study | Cult_CHN_South |
| GZ205 | 01537 | China | Guizhou | Grain | This study | Cult_CHN_South |
| GZ206 | 01538 | China | Guizhou | Grain | This study | Cult_CHN_South |
| GZ207 | 01540 | China | Guizhou | Grain | This study | Cult_CHN_South |
| GZ208 | 01545 | China | Guizhou | Grain | This study | Cult_CHN_South |
| GZ209 | 01547 | China | Guizhou | Grain | This study | Cult_CHN_South |
| GZ21 | Dahongpao | China | Shandong | Grain | This study | Cult_CHN_North |
| GZ210 | 01549 | China | Guizhou | Grain | This study | Cult_CHN_South |
| GZ211 | 01550 | China | Guizhou | Grain | This study |  |
| GZ212 | 01599 | China | Guizhou | Grain | This study | Cult_CHN_South |
| GZ213 | 01734 | China | Guizhou | Grain | This study | Cult_CHN_South |
| GZ214 | 09511 | China | Guizhou | Grain | This study | Cult_CHN_South |
| GZ215 | 09776 | China | Shaanxi | Grain | This study | Cult_CHN_South |
| GZ216 | 09794 | China | Shaanxi | Grain | This study |  |
| GZ217 | 11270 | China | Anhui | Grain | This study |  |
| GZ218 | 11273 | China | Anhui | Grain | This study |  |
| GZ219 | 7097 | China | Jilin | Grain | This study | Cult_CHN_North |
| GZ22 | Diding | China | Hebei | Grain | This study | Cult_CHN_North |
| GZ220 | 7103 | China | Jilin | Grain | This study | Cult_CHN_North |
| GZ221 | 7184 | China | Jilin | Grain | This study | Cult_CHN_North |
| GZ222 | Chishui Hongliang 1 | China | Guizhou | Grain | This study | Cult_CHN_Chishui |
| GZ223 | Chishui Hongliang 2 | China | Guizhou | Grain | This study | Cult_CHN_Chishui |
| GZ224 | Hongtailiang | China | Guizhou | Grain | This study | Cult_CHN_Chishui |
| GZ225 | Maohongnuo 2 | China | Guizhou | Grain | This study | Cult_CHN_Chishui |
| GZ226 | Zunhong1301 | China | Guizhou | Grain | This study |  |
| GZ227 | Hongmaobuo 2 | China | Guizhou | Grain | This study | Cult_CHN_Chishui |
| GZ228 | T38 | China | Guizhou | Grain | This study | Cult_CHN_Chishui |
| GZ229 | Maohongnuo1 | China | Guizhou | Grain | This study | Cult_CHN_Chishui |
| GZ23 | Erhu Sorghum | China | Liaoning | Grain | This study | Cult_CHN_North |
| GZ230 | T4 | China | Sichuan | Grain | This study | Cult_CHN_Chishui |
| GZ232 | Xiaoyingzi | China | Guizhou | Grain | This study |  |
| GZ233 | T22 | China | Sichuan | Grain | This study | Cult_CHN_Chishui |
| GZ235 | Henong 16 | China | Hebei | Grain | This study | Cult_Africa |
| GZ238 | Zigong Sorghum | China | Sichuan | Grain | This study | Cult_CHN_Chishui |
| GZ239 | Hongzhenzhu | China | Sichuan | Grain | This study | Cult_CHN_Chishui |
| GZ24 | Erluhuangke | China | Liaoning | Grain | This study | Cult_CHN_North |
| GZ240 | Langnuo 1 | China | Sichuan | Grain | This study |  |
| GZ241 | Luzhouhong 2 | China | Sichuan | Grain | This study | Cult_CHN_Chishui |
| GZ242 | Qingxuan 2 | China | Sichuan | Grain | This study | Cult_CHN_Chishui |
| GZ243 | Hongkenuo | China | Sichuan | Grain | This study | Cult_CHN_South |
| GZ244 | 125 | China | Guizhou | Grain | This study | Cult_CHN_Chishui |
| GZ245 | Q27 | China | Guizhou | Grain | This study | Cult_CHN_Chishui |
| GZ247 | 16 | China | Guizhou | Grain | This study |  |
| GZ248 | 180 | China | Guizhou | Grain | This study | Cult_CHN_Chishui |
| GZ249 | Qiangao_1 | China | Guizhou | Grain | This study |  |
| GZ25 | Guaibosan | China | Shanxi | Grain | This study | Cult_CHN_North |
| GZ250 | Qiangao_2 | China | Guizhou | Grain | This study |  |
| GZ251 | Qiangao_3 | China | Guizhou | Grain | This study |  |
| GZ252 | Qiangao_4 | China | Guizhou | Grain | This study |  |
| GZ254 | Qiangao_7 | China | Guizhou | Grain | This study | Cult_CHN_Chishui |
| GZ255 | Qiangao_.8 | China | Guizhou | Grain | This study | Cult_CHN_South |
| GZ256 | Xiangnuo_2 | China | Sichuan | Grain | This study |  |
| GZ257 | Datang Sorghum | China | Guangxi | Grain | This study |  |
| GZ26 | Huangluoshan-Jx | China | Liaoning | Grain | This study | Cult_CHN_North |
| GZ27 | Guandongqing | China | Liaoning | Grain | This study | Cult_CHN_North |
| GZ28 | Heike | China | Liaoning | Grain | This study | Cult_CHN_North |
| GZ29 | Hekebai | China | Beijing | Grain | This study | Cult_CHN_North |
| GZ3 | 119 Sorghum | China | Liaoning | Grain | This study | Cult_CHN_North |
| GZ30 | Heikebangzi | China | Liaoning | Grain | This study | Cult_CHN_North |
| GZ31 | Heikeerwei | China | Shanxi | Grain | This study | Cult_CHN_North |
| GZ32 | Heikesheyan | China | Liaoning | Grain | This study | Cult_CHN_North |
| GZ33 | Heisezailaizhong | Japan | Japan | Grain | This study |  |
| GZ34 | Red Stalks Sorghum | China | Shanxi | Grain | This study | Cult_CHN_North |
| GZ35 | Red Stalk Sticky Sorghum | China | Shanxi | Grain | This study | Cult_CHN_North |
| GZ36 | Red Sorghum | China | Inner Mongolia | Grain | This study | Cult_CHN_North |
| GZ37 | Hongke | China | Liaoning | Grain | This study | Cult_CHN_North |
| GZ38 | Hongke Sorghum | China | Shanxi | Grain | This study | Cult_CHN_North |
| GZ39 | Huboxiang | China | Inner Mongolia | Grain | This study | Cult_CHN_North |
| GZ40 | Baidazimao | China | Shanxi | Grain | This study | Cult_CHN_North |
| GZ41 | Ji7384 | China | Jilin | Grain | This study | Cult_CHN_North |
| GZ42 | Jinguang | China | Shanxi | Grain | This study | Cult_CHN_North |
| GZ43 | Tight Ear Sorghum | China | Shanxi | Grain | This study | Cult_CHN_North |
| GZ44 | Jiutouweng | China | Liaoning | Grain | This study |  |
| GZ45 | Jiuyehong | China | Shanxi | Grain | This study | Cult_CHN_North |
| GZ46 | Old Sorghum | China | Inner Mongolia | Grain | This study |  |
| GZ47 | Langweiba-SX | China | Shanxi | Grain | This study | Cult_CHN_North |
| GZ48 | Laomuzhuqiaojiao-NMG | China | Inner Mongolia | Grain | This study | Cult_CHN_North |
| GZ49 | Laomuzhuqiaojiao-HLJ | China | HeiLongjiang | Grain | This study | Cult_CHN_North |
| GZ5 | Banjiang Sorghum-SC | China | Sichuan | Grain | This study |  |
| GZ50 | Liuzi Sorghum | China | Shandong | Grain | This study | Cult_CHN_North |
| GZ51 | Luochuisui | China | Shanxi | Grain | This study | Cult_CHN_North |
| GZ53 | Nanyuanhong 2 | China | Beijing | Grain | This study | Cult_CHN_North |
| GZ54 | Niutui Sorghum | China | Beijing | Grain | This study | Cult_CHN_North |
| GZ55 | Niuweiba Sorghum | China | Shanxi | Grain | This study | Cult_CHN_North |
| GZ56 | Niuxinhuang | China | Liaoning | Grain | This study | Cult_CHN_North |
| GZ57 | Waxy Sorghum-SC | China | Sichuan | Grain | This study | Cult_CHN_South |
| GZ58 | Pingdingxiang-HLJ | China | HeiLongjiang | Grain | This study | Cult_CHN_North |
| GZ59 | Pingdingxiang-JX | China | Liaoning | Grain | This study | Cult_CHN_North |
| GZ6 | Badaye | China | Liaoning | Grain | This study | Cult_CHN_North |
| GZ60 | Qignkeyang | China | Sichuan | Grain | This study | Cult_CHN_Chishui |
| GZ61 | Soft Sorghum | China | Shanxi | Grain | This study | Cult_CHN_North |
| GZ62 | Sansuidamiliang | China | Liaoning | Grain | This study | Cult_CHN_North |
| GZ63 | Sansuihongke | China | Liaoning | Grain | This study | Cult_CHN_North |
| GZ65 | Shangtingsui | China | Shanxi | Grain | This study | Cult_Africa |
| GZ67 | Sorghum Sudanense | Sudan | Sudan | Grain | This study |  |
| GZ68 | Tieshalian | China | Shanxi | Grain | This study | Cult_CHN_North |
| GZ69 | Waibozhang | China | Liaoning | Grain | This study | Cult_CHN_North |
| GZ7 | Bamiancheng | China | Jilin | Grain | This study |  |
| GZ70 | Xiquebai | China | Shanxi | Grain | This study | Cult_CHN_North |
| GZ71 | Xiaobailiang | China | Hebei | Grain | This study |  |
| GZ72 | Xiaobangbanghuang | China | Liaoning | Grain | This study | Cult_CHN_North |
| GZ73 | Little Red Sorghum | China | Inner Mongolia | Grain | This study |  |
| GZ74 | Xiaozhanbang | China | Liaoning | Grain | This study |  |
| GZ75 | Youxinhu | China | Hebei | Grain | This study | Cult_CHN_North |
| GZ76 | Yuejin 4 | China | Liaoning | Grain | This study | Cult_CHN_North |
| GZ77 | Sticky Sorghum-HB | China | Hebei | Grain | This study | Cult_CHN_North |
| GZ78 | Sticky Sorghum-SX | China | Liaoning | Grain | This study | Cult_CHN_North |
| GZ79 | Zhengyansan | China | Shanxi | Grain | This study | Cult_CHN_North |
| GZ8 | Bayeqi | China | Shanxi | Grain | This study | Cult_CHN_North |
| GZ80 | Broom Sorghum | China | Unknown | Grain | This study |  |
| GZ81 | Yong 108 | China | Inner Mongolia | Grain | This study | Cult_CHN_North |
| GZ82 | 32 | China | Liaoning | Grain | This study | Cult_CHN_North |
| GZ83 | 36 | China | Liaoning | Grain | This study | Cult_CHN_North |
| GZ84 | 239 | China | Liaoning | Grain | This study | Cult_CHN_North |
| GZ85 | 43 | China | Liaoning | Grain | This study | Cult_CHN_North |
| GZ86 | 225 | China | Liaoning | Grain | This study | Cult_CHN_North |
| GZ87 | 3008 | China | Liaoning | Grain | This study | Cult_CHN_North |
| GZ88 | 23 | China | Liaoning | Grain | This study | Cult_CHN_North |
| GZ89 | 6 | China | Liaoning | Grain | This study | Cult_CHN_North |
| GZ9 | White Glutinous Sorghum | China | Shaanxi | Grain | This study | Cult_CHN_North |
| GZ90 | 059 | China | Shandong | Grain | This study |  |
| GZ91 | Hongnuo-Ln | China | Liaoning | Grain | This study | Cult_South_Asia |
| GZ92 | 017 | China | Guizhou | Grain | This study |  |
| GZ93 | 565 | China | Beijing | Grain | This study | Cult_CHN_North |
| GZ94 | 459 | China | Beijing | Grain | This study | Cult_CHN_North |
| GZ95 | 485 | China | Beijing | Grain | This study | Cult_CHN_North |
| GZ96 | 426 | China | Beijing | Grain | This study | Cult_CHN_North |
| GZ97 | 658 | China | Beijing | Grain | This study | Cult_CHN_North |
| GZ98 | 212 | China | Beijing | Grain | This study | Cult_CHN_North |
| GZ99 | 2 | China | Hebei | Grain | This study | Cult_CHN_North |
| HYZ | Hongyingzi | China | Guizhou | Grain | This study | Cult_CHN_Chishui |
| J101 | Segaolame | Botswana | Botswana | Grain | Zhang LM_PC | Cult_Africa |
| MHMD | 0 | Kenya | Kenya | Grain | Zhang LM_PC |  |
| SL105 | IS 31366 | Uganda | Uganda | Wild | Zhang LM_PC |  |
| SL108 | IS 31447 | Uganda | Uganda | Wild | Zhang LM_PC |  |
| SL109 | IS 31448 | Uganda | Uganda | Wild | Zhang LM_PC |  |
| SL12 | MN 2963 | Nigeria | Nigeria | Wild | Zhang LM_PC |  |
| SL129 | Oboro | Kenya | Kenya | Wild | Zhang LM_PC |  |
| SL130 | TCD 077 | Chad | Chad | Wild | Zhang LM_PC |  |
| SL136 | 15-B | Ethiopia | Ethiopia | Wild | Zhang LM_PC | Wild_Africa |
| SL137 | 16-B | Ethiopia | Ethiopia | Wild | Zhang LM_PC | Wild_Africa |
| SL138 | Adar | Ethiopia | Ethiopia | Wild | Zhang LM_PC |  |
| SL140 | PB-13 | Burundi | Burundi | Wild | Zhang LM_PC |  |
| SL141 | PK-108 | Burundi | Burundi | Wild | Zhang LM_PC |  |
| SL19 | IS 3106 | Kenya | Kenya | Wild | Zhang LM_PC |  |
| SL25 | IS 2755 | India | India | Wild | Zhang LM_PC | Wild_Africa |
| SL32 | IS 12701 | Tanzania | Tanzania | Wild | Zhang LM_PC |  |
| SL35 | 103-B | Ethiopia | Ethiopia | Wild | Zhang LM_PC | Wild_Africa |
| SL36 | 149-B | Ethiopia | Ethiopia | Wild | Zhang LM_PC | Wild_Africa |
| SL37 | Mashila | Ethiopia | Ethiopia | Wild | Zhang LM_PC | Wild_Africa |
| SL39 | Adar | Eritrea | Eritrea | Grain | Zhang LM_PC |  |
| SL41 | IS 12216 | Uganda | Uganda | Wild | Zhang LM_PC |  |
| SL43 | 148-B | Ethiopia | Ethiopia | Wild | Zhang LM_PC | Wild_Africa |
| SL47 | ZM-1290 | Zambia | Zambia | Wild | Zhang LM_PC |  |
| SL49 | Munyangi | Kenya | Kenya | Wild | Zhang LM_PC | Wild_Africa |
| SL51 | SAD 0036 | Malawi | Malawi | Wild | Zhang LM_PC | Wild_Africa |
| SL53 | MW 019 | Malawi | Malawi | Wild | Zhang LM_PC |  |
| SL62 | 67 | Zimbabwe | Zimbabwe | Wild | Zhang LM_PC | Wild_Africa |
| SL83 | FAO 54912 | Sudan | Sudan | Wild | Zhang LM_PC |  |
| SL97 | SEREKETCH | Sierra Leone | Sierra Leone | Wild | Zhang LM_PC |  |
| Tu14 | 329 | South Africa | South Africa | Wild | Zhang LM_PC | Wild_Africa |
| Tu30 | 1398 | South Africa | South Africa | Wild | Zhang LM_PC | Wild_Africa |
| Tu5 | NA | Zimbabwe | Zimbabwe | Wild | Zhang LM_PC |  |
| WSC100 | AKLMOI WHITE | Sudan | Sudan | Grain | Zhang LM_PC | Cult_Africa |
| WSC104 | CAPE COLO 28/53 | India | India | Grain | Zhang LM_PC | Cult_Africa |
| WSC105 | TSETA (LOCAL NATURE TYPE) 27/51 | India | India | Grain | Zhang LM_PC | Cult_Africa |
| WSC12 | 0 | North Korea | North Korea | Grain | Zhang LM_PC | Cult_CHN_North |
| WSC13 | 0 | North Korea | North Korea | Grain | Zhang LM_PC |  |
| WSC15 | 0 | North Korea | North Korea | Grain | Zhang LM_PC | Cult_CHN_North |
| WSC16 | MOCTAC LOCAL | South Korea | South Korea | Grain | Zhang LM_PC |  |
| WSC17 | 0 | North Korea | North Korea | Grain | Zhang LM_PC | Cult_CHN_North |
| WSC18 | 0 | South Korea | South Korea | Grain | Zhang LM_PC |  |
| WSC29 | KALJANPUR | India | India | Grain | Zhang LM_PC |  |
| WSC30 | SC NO.0217 | India | India | Grain | Zhang LM_PC | Cult_South_Asia |
| WSC31 | GOOSENECK | India | India | Grain | Zhang LM_PC | Cult_South_Asia |
| WSC32 | MARIANGARI JORA MUDDAHIHAL | India | India | Grain | Zhang LM_PC |  |
| WSC34 | RABI YANGAR JORA MITHUGADUR | India | India | Grain | Zhang LM_PC | Cult_South_Asia |
| WSC38 | COL/PAK/1989/IBPGR/2420(1) | Pakistan | Pakistan | Grain | Zhang LM_PC | Cult_South_Asia |
| WSC40 | COL/PAK/1989/IBPGR/2439(1) | Pakistan | Pakistan | Grain | Zhang LM_PC | Cult_South_Asia |
| WSC52 | EC 18868 | Nepal | Nepal | Grain | Zhang LM_PC | Cult_South_Asia |
| WSC54 | PI 229846 VULGARE | South Africa | South Africa | Grain | Zhang LM_PC | Cult_Africa |
| WSC56 | E 9 | Chad | Chad | Grain | Zhang LM_PC |  |
| WSC59 | MAKHOTLONG I | Lesotho | Lesotho | Grain | Zhang LM_PC | Cult_Africa |
| WSC61 | NYAKASOBA BEST | Lesotho | Lesotho | Grain | Zhang LM_PC | Cult_Africa |
| WSC68 | INGWARUMA PEARLY | South Africa | South Africa | Grain | Zhang LM_PC | Cult_Africa |
| WSC70 | BARNARD RED | South Africa | South Africa | Grain | Zhang LM_PC | Cult_Africa |
| WSC72 | S.BASUTORUM DL/60/97 | South Africa | South Africa | Grain | Zhang LM_PC | Cult_Africa |
| WSC73 | EAR FROM PIETERSBURG DL/60/107 | South Africa | South Africa | Grain | Zhang LM_PC | Cult_Africa |
| WSC79 | LAMBAS | Sudan | Sudan | Grain | Zhang LM_PC | Cult_Africa |
| WSC81 | WAD UMM BENEIN | Sudan | Sudan | Grain | Zhang LM_PC | Cult_Africa |
| WSC85 | 109 TONJI | Sudan | Sudan | Grain | Zhang LM_PC | Cult_Africa |
| WSC93 | E 276 FRAMIDA | Kenya | Kenya | Grain | Zhang LM_PC | Cult_Africa |
| WSC94 | UGANDA L 1 | Sudan | Sudan | Grain | Zhang LM_PC | Cult_Africa |
| WSC98 | GIZA 3/59 | India | India | Grain | Zhang LM_PC |  |
| WSC99 | Durra | Ethiopia | Ethiopia | Grain | Zhang LM_PC | Cult_Africa |

**Supplemental Table 2** Analysis results of re-sequencing data of 333 Sorghum accessions

| **Sample** | **Total reads** | **Dup reads** | **Dup percent (%)** | **RmDup reads** | **Mapped reads** | **Mapped Rate (%)** | **Mean coverge (X)** | **Mean mapping quality** | **1X coverage (%)** | **5X coverage (%)** |
| --- | --- | --- | --- | --- | --- | --- | --- | --- | --- | --- |
| 123 | 24454426 | 1134693 | 0.0464 | 23319733 | 20673353 | 0.8865 | 4.5306 | 28.0005 | 0.8724 | 0.3967 |
| 2009 | 40564336 | 19062286 | 0.4699 | 21502050 | 15743144 | 0.7322 | 7.2499 | 26.5724 | 0.8034 | 0.4935 |
| 2010 | 36152402 | 2394779 | 0.0662 | 33757623 | 30002954 | 0.8888 | 6.7679 | 26.9462 | 0.8844 | 0.6466 |
| 2030 | 31236488 | 11641273 | 0.3727 | 19595215 | 17252932 | 0.8805 | 5.9969 | 27.3226 | 0.8275 | 0.4712 |
| 2031 | 35830312 | 13152632 | 0.3671 | 22677680 | 20219765 | 0.8916 | 6.9574 | 27.9562 | 0.8596 | 0.5653 |
| 260 | 23066542 | 1627254 | 0.0705 | 21439288 | 20611647 | 0.9614 | 4.6423 | 24.9306 | 0.8108 | 0.3933 |
| 302546 | 29409124 | 1885242 | 0.0641 | 27523882 | 26496055 | 0.9627 | 5.763 | 15.1515 | 0.6532 | 0.4105 |
| 348 | 36667818 | 3373803 | 0.092 | 33294015 | 31488288 | 0.9458 | 7.253 | 24.7312 | 0.8526 | 0.6226 |
| 353 | 28296714 | 2125288 | 0.0751 | 26171426 | 25108845 | 0.9594 | 5.6845 | 24.8522 | 0.8546 | 0.5254 |
| 355 | 29306972 | 2303438 | 0.0786 | 27003534 | 26370055 | 0.9765 | 5.975 | 25.8145 | 0.8692 | 0.5589 |
| 357 | 32276200 | 2741285 | 0.0849 | 29534915 | 28093450 | 0.9512 | 6.4247 | 24.5773 | 0.8473 | 0.5625 |
| 364 | 24017402 | 1943606 | 0.0809 | 22073796 | 20975227 | 0.9502 | 4.7381 | 23.8384 | 0.7945 | 0.3805 |
| 367 | 25988438 | 2103167 | 0.0809 | 23885271 | 22368793 | 0.9365 | 5.0965 | 26.5515 | 0.8517 | 0.4528 |
| 370 | 24126756 | 1563819 | 0.0648 | 22562937 | 21605281 | 0.9576 | 4.827 | 26.0857 | 0.8418 | 0.4248 |
| 377 | 25493108 | 1431198 | 0.0561 | 24061910 | 23027726 | 0.957 | 5.0858 | 24.757 | 0.8393 | 0.4489 |
| 392 | 31683274 | 2564748 | 0.0809 | 29118526 | 28121410 | 0.9658 | 6.3689 | 24.7065 | 0.8464 | 0.5418 |
| 66 | 27542014 | 9827120 | 0.3568 | 17714894 | 15847052 | 0.8946 | 5.3124 | 26.3864 | 0.8177 | 0.4292 |
| GW102 | 29926356 | 2082948 | 0.0696 | 27843408 | 25325140 | 0.9096 | 5.7193 | 27.5064 | 0.8793 | 0.5537 |
| GW104 | 26664748 | 1777400 | 0.0667 | 24887348 | 22068697 | 0.8867 | 4.9839 | 27.2165 | 0.8721 | 0.4726 |
| GW16 | 31807714 | 2052644 | 0.0645 | 29755070 | 23718038 | 0.7971 | 5.3761 | 26.7077 | 0.8665 | 0.5092 |
| GW2 | 32484928 | 3009306 | 0.0926 | 29475622 | 26732227 | 0.9069 | 6.2218 | 26.7119 | 0.8733 | 0.5898 |
| GW34 | 32679316 | 2175246 | 0.0666 | 30504070 | 25766124 | 0.8447 | 5.8168 | 26.2732 | 0.8651 | 0.534 |
| GW58 | 28503866 | 2005069 | 0.0703 | 26498797 | 25433768 | 0.9598 | 5.7227 | 26.8857 | 0.8759 | 0.547 |
| GW59 | 28273378 | 1808662 | 0.064 | 26464716 | 21767472 | 0.8225 | 4.9214 | 26.4687 | 0.8596 | 0.4551 |
| GW9 | 31051632 | 2322810 | 0.0748 | 28728822 | 27615947 | 0.9613 | 6.2433 | 26.4735 | 0.8705 | 0.5897 |
| GZ1 | 38757922 | 4716903 | 0.1217 | 34041019 | 33187051 | 0.9749 | 7.8294 | 26.7717 | 0.8853 | 0.6622 |
| GZ10 | 42090114 | 6422706 | 0.1526 | 35667408 | 34792313 | 0.9755 | 8.4997 | 26.7436 | 0.8819 | 0.6825 |
| GZ100 | 42455800 | 6067152 | 0.1429 | 36388648 | 35540216 | 0.9767 | 8.6097 | 26.8174 | 0.8848 | 0.7175 |
| GZ101 | 51149426 | 6676943 | 0.1305 | 44472483 | 43581233 | 0.98 | 10.4095 | 26.7733 | 0.8888 | 0.7847 |
| GZ102 | 38992672 | 4260885 | 0.1093 | 34731787 | 34072542 | 0.981 | 7.9526 | 26.6759 | 0.881 | 0.6702 |
| GZ103 | 30693654 | 2926642 | 0.0954 | 27767012 | 27216410 | 0.9802 | 6.2565 | 26.7722 | 0.8756 | 0.5643 |
| GZ104 | 30024478 | 2719165 | 0.0906 | 27305313 | 26695559 | 0.9777 | 6.0897 | 26.7199 | 0.8719 | 0.5397 |
| GZ105 | 28035196 | 2593578 | 0.0925 | 25441618 | 24913809 | 0.9793 | 5.6966 | 26.8083 | 0.868 | 0.4962 |
| GZ106 | 28030104 | 2608902 | 0.0931 | 25421202 | 24870916 | 0.9784 | 5.6757 | 26.6607 | 0.8629 | 0.4819 |
| GZ107 | 27166604 | 2424573 | 0.0892 | 24742031 | 24260108 | 0.9805 | 5.5276 | 26.7157 | 0.865 | 0.4843 |
| GZ108 | 32101330 | 3745370 | 0.1167 | 28355960 | 27758170 | 0.9789 | 6.5166 | 26.5578 | 0.8731 | 0.5705 |
| GZ109 | 25882874 | 2279439 | 0.0881 | 23603435 | 23104357 | 0.9789 | 5.2527 | 26.7267 | 0.8611 | 0.4538 |
| GZ11 | 35822834 | 3712946 | 0.1036 | 32109888 | 31391465 | 0.9776 | 7.2571 | 26.8526 | 0.8781 | 0.6273 |
| GZ110 | 26158412 | 2300086 | 0.0879 | 23858326 | 23308883 | 0.977 | 5.288 | 26.6461 | 0.8619 | 0.4421 |
| GZ111 | 26713276 | 2422058 | 0.0907 | 24291218 | 23730642 | 0.9769 | 5.3929 | 26.7133 | 0.8596 | 0.4504 |
| GZ112 | 43532730 | 5343098 | 0.1227 | 38189632 | 37408067 | 0.9795 | 8.855 | 26.7561 | 0.8842 | 0.7172 |
| GZ113 | 27945800 | 2926532 | 0.1047 | 25019268 | 24324357 | 0.9722 | 5.6228 | 26.6522 | 0.8655 | 0.4823 |
| GZ114 | 30308658 | 3973432 | 0.1311 | 26335226 | 25788307 | 0.9792 | 6.1687 | 26.7468 | 0.8707 | 0.544 |
| GZ115 | 47015338 | 5498941 | 0.117 | 41516397 | 40713146 | 0.9807 | 9.5698 | 26.6085 | 0.8856 | 0.7425 |
| GZ116 | 33422726 | 4629128 | 0.1385 | 28793598 | 28033542 | 0.9736 | 6.7498 | 26.6992 | 0.8728 | 0.5753 |
| GZ117 | 37711752 | 4452223 | 0.1181 | 33259529 | 32536662 | 0.9783 | 7.6316 | 26.6645 | 0.879 | 0.6511 |
| GZ118 | 32947610 | 3928185 | 0.1192 | 29019425 | 28347896 | 0.9769 | 6.6576 | 26.5939 | 0.8716 | 0.5654 |
| GZ119 | 30004638 | 2795871 | 0.0932 | 27208767 | 26695923 | 0.9812 | 6.1161 | 26.7701 | 0.8718 | 0.5447 |
| GZ12 | 32519104 | 3361142 | 0.1034 | 29157962 | 28448482 | 0.9757 | 6.5573 | 26.7314 | 0.8762 | 0.5577 |
| GZ120 | 38239896 | 3918623 | 0.1025 | 34321273 | 33550424 | 0.9775 | 7.7322 | 26.7403 | 0.8795 | 0.649 |
| GZ121 | 32027706 | 4509916 | 0.1408 | 27517790 | 26868973 | 0.9764 | 6.4735 | 26.6833 | 0.8654 | 0.5224 |
| GZ122 | 41126114 | 5309582 | 0.1291 | 35816532 | 35006537 | 0.9774 | 8.3195 | 26.8725 | 0.8861 | 0.6865 |
| GZ123 | 28126170 | 3521399 | 0.1252 | 24604771 | 24014493 | 0.976 | 5.6964 | 26.786 | 0.8639 | 0.4826 |
| GZ124 | 34602576 | 3836739 | 0.1109 | 30765837 | 30110708 | 0.9787 | 7.0202 | 26.6335 | 0.8773 | 0.6057 |
| GZ125 | 31267614 | 4442394 | 0.1421 | 26825220 | 26198873 | 0.9767 | 6.3282 | 26.6356 | 0.8681 | 0.545 |
| GZ126 | 31252324 | 3225418 | 0.1032 | 28026906 | 27408720 | 0.9779 | 6.3333 | 26.6835 | 0.8711 | 0.557 |
| GZ127 | 35184902 | 4070087 | 0.1157 | 31114815 | 30437304 | 0.9782 | 7.1358 | 26.7006 | 0.8787 | 0.6228 |
| GZ128 | 28181926 | 2819291 | 0.1 | 25362635 | 24721822 | 0.9747 | 5.6733 | 26.7161 | 0.8622 | 0.4775 |
| GZ129 | 28346158 | 3761834 | 0.1327 | 24584324 | 24046376 | 0.9781 | 5.7432 | 26.8636 | 0.8663 | 0.4872 |
| GZ13 | 41811198 | 6222469 | 0.1488 | 35588729 | 34796367 | 0.9777 | 8.4881 | 26.7405 | 0.8858 | 0.6949 |
| GZ130 | 31565482 | 3028179 | 0.0959 | 28537303 | 28010628 | 0.9815 | 6.4405 | 26.6151 | 0.8657 | 0.5646 |
| GZ131 | 36611138 | 4134789 | 0.1129 | 32476349 | 31839730 | 0.9804 | 7.4498 | 26.7272 | 0.8776 | 0.6387 |
| GZ132 | 39371416 | 4610465 | 0.1171 | 34760951 | 34046077 | 0.9794 | 8.0121 | 26.6379 | 0.8824 | 0.6929 |
| GZ133 | 41184582 | 4913498 | 0.1193 | 36271084 | 35468244 | 0.9779 | 8.3436 | 26.7058 | 0.883 | 0.6831 |
| GZ134 | 35700508 | 4436768 | 0.1243 | 31263740 | 30582487 | 0.9782 | 7.2427 | 26.776 | 0.8785 | 0.6263 |
| GZ135 | 34116220 | 3773315 | 0.1106 | 30342905 | 29592449 | 0.9753 | 6.8807 | 26.8753 | 0.8786 | 0.5894 |
| GZ136 | 33958208 | 4665276 | 0.1374 | 29292932 | 28639151 | 0.9777 | 6.8859 | 26.7691 | 0.879 | 0.5944 |
| GZ138 | 32723360 | 3619147 | 0.1106 | 29104213 | 28545927 | 0.9808 | 6.6644 | 26.6885 | 0.8742 | 0.5908 |
| GZ139 | 28044230 | 3586906 | 0.1279 | 24457324 | 24028544 | 0.9825 | 5.7322 | 26.8945 | 0.8695 | 0.5041 |
| GZ14 | 46685626 | 6740687 | 0.1444 | 39944939 | 38308476 | 0.959 | 9.305 | 26.6887 | 0.8854 | 0.7338 |
| GZ140 | 26387294 | 2619672 | 0.0993 | 23767622 | 23268467 | 0.979 | 5.3543 | 26.8834 | 0.8667 | 0.4644 |
| GZ141 | 38058082 | 4226787 | 0.1111 | 33831295 | 33302548 | 0.9844 | 7.8023 | 26.7693 | 0.8819 | 0.6628 |
| GZ143 | 30143276 | 3101255 | 0.1029 | 27042021 | 26485587 | 0.9794 | 6.1379 | 26.8234 | 0.8714 | 0.5476 |
| GZ144 | 43104772 | 5809226 | 0.1348 | 37295546 | 36511487 | 0.979 | 8.7694 | 26.8342 | 0.8855 | 0.72 |
| GZ145 | 33423578 | 4663503 | 0.1395 | 28760075 | 28209328 | 0.9809 | 6.8143 | 26.474 | 0.8743 | 0.5926 |
| GZ146 | 28412466 | 2469133 | 0.0869 | 25943333 | 25388267 | 0.9786 | 5.7718 | 26.7579 | 0.8707 | 0.5108 |
| GZ147 | 33452192 | 3910327 | 0.1169 | 29541865 | 28932058 | 0.9794 | 6.8008 | 26.6687 | 0.8757 | 0.5954 |
| GZ148 | 34078046 | 2973283 | 0.0872 | 31104763 | 30571424 | 0.9829 | 6.9639 | 26.7491 | 0.8783 | 0.6249 |
| GZ149 | 29902232 | 2664831 | 0.0891 | 27237401 | 26693300 | 0.98 | 6.0816 | 26.66 | 0.872 | 0.5441 |
| GZ15 | 31093590 | 2686559 | 0.0864 | 28407031 | 27841972 | 0.9801 | 6.3428 | 26.8011 | 0.8744 | 0.5753 |
| GZ150 | 40300328 | 4536586 | 0.1126 | 35763742 | 35123449 | 0.9821 | 8.2322 | 26.8317 | 0.8837 | 0.7013 |
| GZ151 | 35593560 | 3849481 | 0.1082 | 31744079 | 31093857 | 0.9795 | 7.2484 | 26.9674 | 0.8845 | 0.6421 |
| GZ152 | 33862686 | 3867947 | 0.1142 | 29994739 | 29284167 | 0.9763 | 6.8837 | 26.5388 | 0.877 | 0.6117 |
| GZ153 | 31596130 | 4445424 | 0.1407 | 27150706 | 26618755 | 0.9804 | 6.4319 | 26.7251 | 0.8699 | 0.5603 |
| GZ154 | 25129546 | 2175807 | 0.0866 | 22953739 | 22526712 | 0.9814 | 5.1202 | 26.7026 | 0.8593 | 0.4476 |
| GZ155 | 33136730 | 3731678 | 0.1126 | 29405052 | 28863754 | 0.9816 | 6.7669 | 26.7814 | 0.88 | 0.6066 |
| GZ156 | 36768048 | 3978625 | 0.1082 | 32789423 | 32212317 | 0.9824 | 7.5193 | 26.813 | 0.886 | 0.6674 |
| GZ157 | 48111088 | 5892564 | 0.1225 | 42218524 | 41279120 | 0.9777 | 9.7681 | 26.8155 | 0.8875 | 0.7655 |
| GZ158 | 30606388 | 4065774 | 0.1328 | 26540614 | 26037436 | 0.981 | 6.2414 | 26.7783 | 0.8745 | 0.5534 |
| GZ159 | 27910998 | 2428836 | 0.087 | 25482162 | 24997966 | 0.981 | 5.6778 | 26.7018 | 0.866 | 0.5 |
| GZ16 | 30194796 | 3450816 | 0.1143 | 26743980 | 25977822 | 0.9714 | 6.0636 | 26.7993 | 0.8674 | 0.5086 |
| GZ160 | 28517294 | 3835409 | 0.1345 | 24681885 | 24035117 | 0.9738 | 5.7447 | 26.689 | 0.8605 | 0.4731 |
| GZ161 | 31449830 | 2707669 | 0.0861 | 28742161 | 28178370 | 0.9804 | 6.3994 | 26.8805 | 0.8741 | 0.5773 |
| GZ162 | 30293334 | 3960428 | 0.1307 | 26332906 | 25810811 | 0.9802 | 6.1685 | 26.8139 | 0.8685 | 0.538 |
| GZ163 | 31329902 | 3313847 | 0.1058 | 28016055 | 27463552 | 0.9803 | 6.3771 | 26.6679 | 0.8712 | 0.566 |
| GZ164 | 31533116 | 3305786 | 0.1048 | 28227330 | 27778348 | 0.9841 | 6.4612 | 26.7309 | 0.8754 | 0.5894 |
| GZ165 | 28150376 | 2655376 | 0.0943 | 25495000 | 24643089 | 0.9666 | 5.6707 | 26.9028 | 0.8718 | 0.5151 |
| GZ166 | 45509952 | 5730123 | 0.1259 | 39779829 | 38882333 | 0.9774 | 9.2391 | 26.9157 | 0.8857 | 0.7338 |
| GZ167 | 29700426 | 2698489 | 0.0909 | 27001937 | 26549551 | 0.9832 | 6.0732 | 26.7209 | 0.8711 | 0.5463 |
| GZ168 | 42014608 | 4453384 | 0.106 | 37561224 | 36719832 | 0.9776 | 8.5399 | 26.8327 | 0.8887 | 0.7213 |
| GZ169 | 30370554 | 2577342 | 0.0849 | 27793212 | 27171533 | 0.9776 | 6.1754 | 26.9255 | 0.877 | 0.5639 |
| GZ17 | 28955416 | 2368848 | 0.0818 | 26586568 | 25993334 | 0.9777 | 5.8683 | 26.8545 | 0.8739 | 0.5084 |
| GZ170 | 36861412 | 3708929 | 0.1006 | 33152483 | 32523244 | 0.981 | 7.5221 | 26.7397 | 0.8798 | 0.6548 |
| GZ171 | 40288492 | 4310684 | 0.107 | 35977808 | 35345020 | 0.9824 | 8.2298 | 26.8577 | 0.8837 | 0.7105 |
| GZ172 | 32872158 | 3468534 | 0.1055 | 29403624 | 28731850 | 0.9772 | 6.6609 | 26.8961 | 0.8767 | 0.5825 |
| GZ173 | 47692220 | 5790424 | 0.1214 | 41901796 | 41123428 | 0.9814 | 9.7459 | 26.4717 | 0.8863 | 0.7617 |
| GZ174 | 40149818 | 4585926 | 0.1142 | 35563892 | 34779940 | 0.978 | 8.1398 | 26.7013 | 0.8877 | 0.6812 |
| GZ175 | 31921220 | 3271590 | 0.1025 | 28649630 | 28133679 | 0.982 | 6.5161 | 26.744 | 0.8738 | 0.5889 |
| GZ176 | 40502514 | 4829194 | 0.1192 | 35673320 | 34894409 | 0.9782 | 8.2415 | 26.9476 | 0.8851 | 0.6947 |
| GZ177 | 30737964 | 2871735 | 0.0934 | 27866229 | 27218948 | 0.9768 | 6.2379 | 26.7422 | 0.8736 | 0.5399 |
| GZ178 | 43048730 | 5007235 | 0.1163 | 38041495 | 37150235 | 0.9766 | 8.7647 | 26.963 | 0.8914 | 0.7384 |
| GZ179 | 29683032 | 3112443 | 0.1049 | 26570589 | 26039873 | 0.98 | 6.0496 | 26.8796 | 0.8745 | 0.5523 |
| GZ18 | 28046202 | 2314803 | 0.0825 | 25731399 | 25162436 | 0.9779 | 5.7055 | 26.8289 | 0.8696 | 0.5168 |
| GZ180 | 27507152 | 2482742 | 0.0903 | 25024410 | 24533211 | 0.9804 | 5.594 | 26.6245 | 0.8616 | 0.4821 |
| GZ181 | 30188024 | 2961543 | 0.0981 | 27226481 | 26575507 | 0.9761 | 6.0988 | 26.7956 | 0.8696 | 0.5271 |
| GZ182 | 28416026 | 2230604 | 0.0785 | 26185422 | 25579959 | 0.9769 | 5.7575 | 26.8656 | 0.8683 | 0.5114 |
| GZ183 | 28508842 | 2647242 | 0.0929 | 25861600 | 24959474 | 0.9651 | 5.7295 | 26.9089 | 0.8713 | 0.5124 |
| GZ184 | 24979368 | 1871895 | 0.0749 | 23107473 | 22685886 | 0.9818 | 5.09 | 26.7391 | 0.8648 | 0.4389 |
| GZ186 | 32997270 | 3014712 | 0.0914 | 29982558 | 29372385 | 0.9796 | 6.7095 | 26.6535 | 0.8755 | 0.5943 |
| GZ188 | 43976096 | 5364622 | 0.122 | 38611474 | 37817353 | 0.9794 | 8.9468 | 26.8211 | 0.887 | 0.7206 |
| GZ189 | 24004084 | 5446294 | 0.2269 | 18557790 | 18112207 | 0.976 | 4.8704 | 26.7422 | 0.8325 | 0.3925 |
| GZ19 | 47178412 | 8314028 | 0.1762 | 38864384 | 37842318 | 0.9737 | 9.5115 | 26.8075 | 0.8856 | 0.7259 |
| GZ190 | 37369262 | 4064205 | 0.1088 | 33305057 | 32351271 | 0.9714 | 7.5402 | 26.5953 | 0.8773 | 0.6363 |
| GZ191 | 24967180 | 6145466 | 0.2461 | 18821714 | 18364352 | 0.9757 | 5.0786 | 26.6935 | 0.825 | 0.4023 |
| GZ192 | 36428104 | 4388348 | 0.1205 | 32039756 | 31304471 | 0.9771 | 7.3737 | 26.8887 | 0.8739 | 0.6018 |
| GZ193 | 25446002 | 2042517 | 0.0803 | 23403485 | 22141817 | 0.9461 | 5.0161 | 26.3516 | 0.8515 | 0.4351 |
| GZ194 | 34554556 | 4122412 | 0.1193 | 30432144 | 29774565 | 0.9784 | 7.012 | 26.6952 | 0.8763 | 0.6165 |
| GZ195 | 26793256 | 2281589 | 0.0852 | 24511667 | 24014304 | 0.9797 | 5.445 | 26.8337 | 0.8639 | 0.4761 |
| GZ196 | 41403650 | 5647935 | 0.1364 | 35755715 | 34972496 | 0.9781 | 8.4166 | 26.9083 | 0.8862 | 0.7002 |
| GZ197 | 24861488 | 6011129 | 0.2418 | 18850359 | 18418540 | 0.9771 | 5.0732 | 26.706 | 0.84 | 0.4312 |
| GZ198 | 27897984 | 2460534 | 0.0882 | 25437450 | 24838505 | 0.9765 | 5.643 | 26.5235 | 0.8635 | 0.4906 |
| GZ199 | 28877198 | 2921932 | 0.1012 | 25955266 | 25479434 | 0.9817 | 5.9009 | 26.5853 | 0.8694 | 0.5387 |
| GZ2 | 38635960 | 4609043 | 0.1193 | 34026917 | 33161077 | 0.9746 | 7.7901 | 26.5001 | 0.8843 | 0.6438 |
| GZ20 | 47140438 | 6632624 | 0.1407 | 40507814 | 39610938 | 0.9779 | 9.563 | 26.6269 | 0.8859 | 0.735 |
| GZ200 | 43000022 | 4724094 | 0.1099 | 38275928 | 37640868 | 0.9834 | 8.7969 | 26.9244 | 0.886 | 0.7332 |
| GZ201 | 24112126 | 1999220 | 0.0829 | 22112906 | 21623946 | 0.9779 | 4.8836 | 26.7541 | 0.8527 | 0.4061 |
| GZ202 | 30491394 | 2601061 | 0.0853 | 27890333 | 27256657 | 0.9773 | 6.1853 | 26.8252 | 0.8745 | 0.5603 |
| GZ203 | 34825322 | 3707280 | 0.1065 | 31118042 | 30397309 | 0.9768 | 7.0738 | 26.7989 | 0.8798 | 0.6367 |
| GZ204 | 30503668 | 3045992 | 0.0999 | 27457676 | 26820282 | 0.9768 | 6.1623 | 26.8 | 0.8694 | 0.5279 |
| GZ205 | 27957746 | 2465407 | 0.0882 | 25492339 | 24890763 | 0.9764 | 5.6465 | 26.7482 | 0.8653 | 0.4992 |
| GZ206 | 32868572 | 3179100 | 0.0967 | 29689472 | 28290388 | 0.9529 | 6.4931 | 26.8661 | 0.8723 | 0.5646 |
| GZ207 | 42107854 | 4821519 | 0.1145 | 37286335 | 36481924 | 0.9784 | 8.5648 | 26.594 | 0.8803 | 0.7143 |
| GZ208 | 31945144 | 3575624 | 0.1119 | 28369520 | 27776548 | 0.9791 | 6.5027 | 26.7085 | 0.8748 | 0.5907 |
| GZ209 | 31276198 | 3278262 | 0.1048 | 27997936 | 27446154 | 0.9803 | 6.3738 | 26.6834 | 0.8719 | 0.5712 |
| GZ21 | 29634384 | 2539337 | 0.0857 | 27095047 | 26493336 | 0.9778 | 6.0114 | 26.7382 | 0.8702 | 0.5358 |
| GZ210 | 33275192 | 4023140 | 0.1209 | 29252052 | 28474156 | 0.9734 | 6.6244 | 26.6148 | 0.8705 | 0.5663 |
| GZ211 | 30304032 | 3423551 | 0.113 | 26880481 | 26189058 | 0.9743 | 6.109 | 26.7313 | 0.8667 | 0.5185 |
| GZ212 | 32213378 | 3536049 | 0.1098 | 28677329 | 27919213 | 0.9736 | 6.4311 | 26.7444 | 0.8731 | 0.573 |
| GZ213 | 35738028 | 4055454 | 0.1135 | 31682574 | 30891097 | 0.975 | 7.2191 | 26.8508 | 0.8795 | 0.6155 |
| GZ214 | 42095626 | 5284268 | 0.1255 | 36811358 | 35747489 | 0.9711 | 8.4342 | 26.7338 | 0.8797 | 0.6823 |
| GZ215 | 31573342 | 8229994 | 0.2607 | 23343348 | 22705327 | 0.9727 | 6.4029 | 26.6667 | 0.8562 | 0.5239 |
| GZ216 | 27366956 | 6266909 | 0.229 | 21100047 | 20623556 | 0.9774 | 5.5687 | 26.5464 | 0.8506 | 0.4601 |
| GZ217 | 33653306 | 4047620 | 0.1203 | 29605686 | 28862043 | 0.9749 | 6.7899 | 26.7403 | 0.8767 | 0.5724 |
| GZ218 | 40780904 | 4671132 | 0.1145 | 36109772 | 35238482 | 0.9759 | 8.2336 | 26.6174 | 0.8814 | 0.676 |
| GZ219 | 32248628 | 3441653 | 0.1067 | 28806975 | 27980336 | 0.9713 | 6.4144 | 26.5676 | 0.8722 | 0.5633 |
| GZ22 | 32264846 | 2541003 | 0.0788 | 29723843 | 29089217 | 0.9786 | 6.5569 | 26.8043 | 0.8808 | 0.5905 |
| GZ220 | 28084240 | 6764073 | 0.2408 | 21320167 | 20779306 | 0.9746 | 5.6952 | 26.7915 | 0.8516 | 0.4708 |
| GZ221 | 30844718 | 4184818 | 0.1357 | 26659900 | 26011123 | 0.9757 | 6.2329 | 26.69 | 0.8683 | 0.5237 |
| GZ222 | 41509760 | 4561882 | 0.1099 | 36947878 | 36169851 | 0.9789 | 8.4379 | 26.8721 | 0.8849 | 0.7056 |
| GZ223 | 28922178 | 2812889 | 0.0973 | 26109289 | 25530197 | 0.9778 | 5.8661 | 26.8904 | 0.8675 | 0.5113 |
| GZ224 | 34294644 | 3754988 | 0.1095 | 30539656 | 29711970 | 0.9729 | 6.9373 | 26.8339 | 0.8776 | 0.6189 |
| GZ225 | 35305914 | 3646107 | 0.1033 | 31659807 | 30939670 | 0.9773 | 7.1441 | 26.8002 | 0.876 | 0.6103 |
| GZ226 | 46157850 | 5586056 | 0.121 | 40571794 | 39783814 | 0.9806 | 9.4106 | 27.4782 | 0.8948 | 0.7612 |
| GZ227 | 42882960 | 6129408 | 0.1429 | 36753552 | 35903833 | 0.9769 | 8.7079 | 26.8629 | 0.8827 | 0.7066 |
| GZ228 | 29306394 | 7366533 | 0.2514 | 21939861 | 21373238 | 0.9742 | 5.9536 | 26.8521 | 0.8557 | 0.5 |
| GZ229 | 34937982 | 3768155 | 0.1079 | 31169827 | 30472646 | 0.9776 | 7.0038 | 26.8259 | 0.8776 | 0.6182 |
| GZ23 | 32226772 | 4255477 | 0.132 | 27971295 | 26971130 | 0.9642 | 6.4597 | 26.7653 | 0.8709 | 0.5608 |
| GZ230 | 23954840 | 5726566 | 0.2391 | 18228274 | 17806202 | 0.9768 | 4.8781 | 26.7716 | 0.8349 | 0.4032 |
| GZ232 | 28189842 | 6806624 | 0.2415 | 21383218 | 20781990 | 0.9719 | 5.6964 | 26.8547 | 0.8473 | 0.4576 |
| GZ233 | 38362444 | 4588454 | 0.1196 | 33773990 | 32881719 | 0.9736 | 7.7395 | 26.792 | 0.8791 | 0.6527 |
| GZ235 | 248751070 | 37977064 | 0.1527 | 210774006 | 205573986 | 0.9753 | 50.4227 | 28.2777 | 0.9252 | 0.9115 |
| GZ238 | 25037726 | 6396014 | 0.2555 | 18641712 | 17994251 | 0.9653 | 5.0107 | 26.7048 | 0.8267 | 0.3769 |
| GZ239 | 37183576 | 4561104 | 0.1227 | 32622472 | 31739155 | 0.9729 | 7.3874 | 26.7173 | 0.8777 | 0.6252 |
| GZ24 | 37494060 | 5098974 | 0.136 | 32395086 | 31679269 | 0.9779 | 7.6312 | 26.8751 | 0.8808 | 0.661 |
| GZ240 | 33517478 | 4239714 | 0.1265 | 29277764 | 28474127 | 0.9726 | 6.666 | 26.5851 | 0.8719 | 0.5712 |
| GZ241 | 30690854 | 3478589 | 0.1133 | 27212265 | 26425946 | 0.9711 | 6.162 | 26.8084 | 0.8683 | 0.5179 |
| GZ242 | 29814320 | 7180537 | 0.2408 | 22633783 | 21651972 | 0.9566 | 5.9454 | 26.8636 | 0.8503 | 0.4779 |
| GZ243 | 39151498 | 4810695 | 0.1229 | 34340803 | 33354047 | 0.9713 | 7.8589 | 26.7921 | 0.8791 | 0.6509 |
| GZ244 | 26226730 | 6701834 | 0.2555 | 19524896 | 18947735 | 0.9704 | 5.2901 | 26.8472 | 0.8342 | 0.4164 |
| GZ245 | 36357158 | 4175488 | 0.1148 | 32181670 | 31324089 | 0.9734 | 7.341 | 26.8736 | 0.8796 | 0.6286 |
| GZ247 | 35007456 | 4084822 | 0.1167 | 30922634 | 30136582 | 0.9746 | 7.0568 | 26.7553 | 0.8795 | 0.5896 |
| GZ248 | 35894630 | 4059178 | 0.1131 | 31835452 | 31070404 | 0.976 | 7.2587 | 26.8475 | 0.8771 | 0.6184 |
| GZ249 | 36347908 | 4262048 | 0.1173 | 32085860 | 31301814 | 0.9756 | 7.3804 | 27.7599 | 0.9148 | 0.6639 |
| GZ25 | 33220446 | 4440610 | 0.1337 | 28779836 | 27775176 | 0.9651 | 6.6523 | 26.7045 | 0.8709 | 0.559 |
| GZ250 | 33330348 | 3479256 | 0.1044 | 29851092 | 28737378 | 0.9627 | 6.643 | 27.3453 | 0.884 | 0.5773 |
| GZ251 | 27699284 | 6692514 | 0.2416 | 21006770 | 20395890 | 0.9709 | 5.583 | 27.8737 | 0.8645 | 0.4566 |
| GZ252 | 33175414 | 3445581 | 0.1039 | 29729833 | 29172389 | 0.9812 | 6.7675 | 28.11 | 0.8955 | 0.6257 |
| GZ254 | 46838544 | 5169363 | 0.1104 | 41669181 | 40272121 | 0.9665 | 9.4288 | 26.8298 | 0.8868 | 0.7568 |
| GZ255 | 33288848 | 3760313 | 0.113 | 29528535 | 28875442 | 0.9779 | 6.6777 | 26.7066 | 0.8751 | 0.5898 |
| GZ256 | 29735612 | 2894590 | 0.0973 | 26841022 | 26263444 | 0.9785 | 6.0722 | 28.4501 | 0.912 | 0.5665 |
| GZ257 | 27856556 | 6907148 | 0.248 | 20949408 | 20289403 | 0.9685 | 5.6118 | 26.8211 | 0.8479 | 0.4518 |
| GZ26 | 35256872 | 3767360 | 0.1069 | 31489512 | 30732922 | 0.976 | 7.1184 | 26.7786 | 0.8776 | 0.6077 |
| GZ27 | 35394900 | 4152011 | 0.1173 | 31242889 | 30583035 | 0.9789 | 7.1777 | 26.8052 | 0.8783 | 0.6296 |
| GZ28 | 31360150 | 2667568 | 0.0851 | 28692582 | 28126511 | 0.9803 | 6.3522 | 26.4977 | 0.8731 | 0.5681 |
| GZ29 | 31423064 | 2525178 | 0.0804 | 28897886 | 28350411 | 0.9811 | 6.403 | 26.8802 | 0.8759 | 0.5774 |
| GZ3 | 41650858 | 6228755 | 0.1495 | 35422103 | 34454527 | 0.9727 | 8.3704 | 26.5329 | 0.8814 | 0.6749 |
| GZ30 | 44216336 | 6013460 | 0.136 | 38202876 | 37403915 | 0.9791 | 9.0313 | 26.7949 | 0.8867 | 0.7432 |
| GZ31 | 29572326 | 3004159 | 0.1016 | 26568167 | 25935075 | 0.9762 | 5.9712 | 26.8434 | 0.872 | 0.5126 |
| GZ32 | 33928748 | 4805676 | 0.1416 | 29123072 | 28539215 | 0.98 | 6.9145 | 26.619 | 0.8736 | 0.6038 |
| GZ33 | 42851710 | 5461063 | 0.1274 | 37390647 | 36647884 | 0.9801 | 8.7357 | 26.9836 | 0.9159 | 0.7282 |
| GZ34 | 44243020 | 6700364 | 0.1514 | 37542656 | 36707411 | 0.9778 | 8.9902 | 26.6546 | 0.8846 | 0.7299 |
| GZ35 | 33223318 | 3318658 | 0.0999 | 29904660 | 29318785 | 0.9804 | 6.7581 | 26.6953 | 0.8761 | 0.5917 |
| GZ36 | 43834918 | 5951917 | 0.1358 | 37883001 | 36860760 | 0.973 | 8.8303 | 26.7837 | 0.8899 | 0.7219 |
| GZ37 | 35179840 | 3459266 | 0.0983 | 31720574 | 31073995 | 0.9796 | 7.1384 | 26.7774 | 0.8768 | 0.6217 |
| GZ38 | 43181904 | 6215370 | 0.1439 | 36966534 | 36094922 | 0.9764 | 8.759 | 26.643 | 0.8861 | 0.7077 |
| GZ39 | 27834040 | 2590383 | 0.0931 | 25243657 | 24661600 | 0.9769 | 5.6372 | 26.8445 | 0.8665 | 0.4967 |
| GZ40 | 34861434 | 3341067 | 0.0958 | 31520367 | 30640478 | 0.9721 | 7.0279 | 26.8196 | 0.8796 | 0.621 |
| GZ41 | 48480568 | 7223317 | 0.149 | 41257251 | 40257411 | 0.9758 | 9.822 | 26.6189 | 0.8864 | 0.7536 |
| GZ42 | 31851538 | 2732575 | 0.0858 | 29118963 | 28616186 | 0.9827 | 6.5055 | 26.8359 | 0.8759 | 0.586 |
| GZ43 | 27525426 | 2418090 | 0.0878 | 25107336 | 24325627 | 0.9689 | 5.5576 | 26.5277 | 0.8629 | 0.4937 |
| GZ44 | 29453006 | 3728600 | 0.1266 | 25724406 | 25064555 | 0.9743 | 5.9816 | 26.8179 | 0.8717 | 0.538 |
| GZ45 | 41885210 | 6018304 | 0.1437 | 35866906 | 35094163 | 0.9785 | 8.515 | 26.7582 | 0.8915 | 0.7114 |
| GZ46 | 28907200 | 3710383 | 0.1284 | 25196817 | 24565013 | 0.9749 | 5.8613 | 26.7091 | 0.8659 | 0.5151 |
| GZ47 | 41459250 | 5807451 | 0.1401 | 35651799 | 34937261 | 0.98 | 8.4615 | 26.6129 | 0.8825 | 0.7024 |
| GZ48 | 31038330 | 2782122 | 0.0896 | 28256208 | 27710710 | 0.9807 | 6.3209 | 26.7384 | 0.8726 | 0.5616 |
| GZ49 | 34398500 | 3496616 | 0.1017 | 30901884 | 30221055 | 0.978 | 6.9597 | 26.5175 | 0.8757 | 0.6065 |
| GZ5 | 49606454 | 7879155 | 0.1588 | 41727299 | 40493053 | 0.9704 | 10.0019 | 26.7881 | 0.8877 | 0.755 |
| GZ50 | 34984160 | 3730444 | 0.1066 | 31253716 | 30632791 | 0.9801 | 7.1245 | 26.7613 | 0.8792 | 0.6311 |
| GZ51 | 28503764 | 2382592 | 0.0836 | 26121172 | 25644164 | 0.9817 | 5.8123 | 26.5793 | 0.8686 | 0.5262 |
| GZ53 | 30424206 | 5172431 | 0.17 | 25251775 | 24532672 | 0.9715 | 6.1397 | 26.6377 | 0.8648 | 0.5209 |
| GZ54 | 28991236 | 2497666 | 0.0862 | 26493570 | 25987996 | 0.9809 | 5.9015 | 26.6325 | 0.8694 | 0.5224 |
| GZ55 | 27434542 | 2345523 | 0.0855 | 25089019 | 24555057 | 0.9787 | 5.5698 | 26.6932 | 0.8656 | 0.4907 |
| GZ56 | 33056348 | 3449442 | 0.1044 | 29606906 | 28977743 | 0.9787 | 6.714 | 26.5149 | 0.8748 | 0.5915 |
| GZ57 | 40825106 | 4712862 | 0.1154 | 36112244 | 35467152 | 0.9821 | 8.3408 | 26.7692 | 0.8813 | 0.7087 |
| GZ58 | 36588594 | 5068595 | 0.1385 | 31519999 | 30873962 | 0.9795 | 7.4395 | 26.7415 | 0.8808 | 0.6418 |
| GZ59 | 41207194 | 4548127 | 0.1104 | 36659067 | 35937139 | 0.9803 | 8.3765 | 26.732 | 0.8886 | 0.7003 |
| GZ6 | 44539068 | 6918235 | 0.1553 | 37620833 | 36746815 | 0.9768 | 9.0094 | 26.7959 | 0.8843 | 0.7123 |
| GZ60 | 38570700 | 4058753 | 0.1052 | 34511947 | 33939956 | 0.9834 | 7.8979 | 26.8338 | 0.8825 | 0.6973 |
| GZ61 | 28916824 | 3733502 | 0.1291 | 25183322 | 24730418 | 0.982 | 5.9014 | 26.6635 | 0.8667 | 0.5188 |
| GZ62 | 39943986 | 5709842 | 0.1429 | 34234144 | 33562773 | 0.9804 | 8.1436 | 26.6156 | 0.8822 | 0.6843 |
| GZ63 | 36269556 | 3850327 | 0.1062 | 32419229 | 31802013 | 0.981 | 7.3896 | 26.6669 | 0.8824 | 0.6447 |
| GZ65 | 32756330 | 4731645 | 0.1444 | 28024685 | 27507708 | 0.9816 | 6.6904 | 28.3538 | 0.9043 | 0.6116 |
| GZ67 | 30074496 | 3811393 | 0.1267 | 26263103 | 25657639 | 0.9769 | 6.0603 | 27.0662 | 0.9035 | 0.5193 |
| GZ68 | 41763944 | 5196192 | 0.1244 | 36567752 | 35833602 | 0.9799 | 8.4923 | 26.6098 | 0.8829 | 0.7001 |
| GZ69 | 46486030 | 6888144 | 0.1482 | 39597886 | 38727034 | 0.978 | 9.4296 | 26.6971 | 0.886 | 0.7387 |
| GZ7 | 44970730 | 7543930 | 0.1678 | 37426800 | 36337637 | 0.9709 | 9.0462 | 27.6129 | 0.9066 | 0.7204 |
| GZ70 | 34330614 | 3542513 | 0.1032 | 30788101 | 30198356 | 0.9808 | 6.9913 | 26.6118 | 0.8742 | 0.5956 |
| GZ71 | 28320810 | 3937226 | 0.139 | 24383584 | 23849083 | 0.9781 | 5.7479 | 27.7477 | 0.8997 | 0.4971 |
| GZ72 | 38670708 | 4310938 | 0.1115 | 34359770 | 33618524 | 0.9784 | 7.8488 | 26.6908 | 0.882 | 0.6675 |
| GZ73 | 34918946 | 3672216 | 0.1052 | 31246730 | 30687143 | 0.9821 | 7.1286 | 27.3981 | 0.8968 | 0.6308 |
| GZ74 | 33790800 | 4621814 | 0.1368 | 29168986 | 28654280 | 0.9824 | 6.8983 | 27.1591 | 0.9034 | 0.5984 |
| GZ75 | 39506102 | 5470280 | 0.1385 | 34035822 | 33410150 | 0.9816 | 8.0677 | 26.7411 | 0.8819 | 0.6776 |
| GZ76 | 29341996 | 3831686 | 0.1306 | 25510310 | 24993845 | 0.9798 | 5.9744 | 26.7809 | 0.8707 | 0.5322 |
| GZ77 | 46439716 | 6599167 | 0.1421 | 39840549 | 39005464 | 0.979 | 9.4545 | 26.8047 | 0.888 | 0.7499 |
| GZ78 | 42827620 | 5945680 | 0.1388 | 36881940 | 36111899 | 0.9791 | 8.6917 | 26.6045 | 0.8823 | 0.7071 |
| GZ79 | 41579418 | 3942978 | 0.0948 | 37636440 | 36879664 | 0.9799 | 8.4774 | 26.6948 | 0.8854 | 0.7197 |
| GZ8 | 30826690 | 3266121 | 0.106 | 27560569 | 26873950 | 0.9751 | 6.2121 | 26.8398 | 0.8741 | 0.5258 |
| GZ80 | 46380860 | 6808572 | 0.1468 | 39572288 | 38812609 | 0.9808 | 9.4514 | 27.1047 | 0.9002 | 0.7536 |
| GZ81 | 34508646 | 4116386 | 0.1193 | 30392260 | 29740417 | 0.9786 | 7.0074 | 26.818 | 0.8758 | 0.6092 |
| GZ82 | 29670670 | 2814365 | 0.0949 | 26856305 | 26256215 | 0.9777 | 6.0103 | 26.714 | 0.87 | 0.5331 |
| GZ83 | 47621802 | 5544058 | 0.1164 | 42077744 | 41292847 | 0.9813 | 9.704 | 26.8046 | 0.8874 | 0.7615 |
| GZ84 | 34247218 | 3632903 | 0.1061 | 30614315 | 29974380 | 0.9791 | 6.9227 | 26.6727 | 0.8765 | 0.5965 |
| GZ85 | 28947622 | 2428954 | 0.0839 | 26518668 | 25899968 | 0.9767 | 5.8622 | 26.7034 | 0.869 | 0.5157 |
| GZ86 | 29521516 | 3884339 | 0.1316 | 25637177 | 25091112 | 0.9787 | 6.0037 | 26.6719 | 0.8679 | 0.5241 |
| GZ87 | 28820704 | 3106883 | 0.1078 | 25713821 | 25235074 | 0.9814 | 5.8764 | 26.8668 | 0.872 | 0.5205 |
| GZ88 | 42578124 | 5231141 | 0.1229 | 37346983 | 36484140 | 0.9769 | 8.6641 | 26.6536 | 0.8841 | 0.73 |
| GZ89 | 39187134 | 4492982 | 0.1147 | 34694152 | 33847840 | 0.9756 | 7.9288 | 26.8828 | 0.882 | 0.6744 |
| GZ9 | 33481724 | 2800629 | 0.0836 | 30681095 | 30149065 | 0.9827 | 6.8429 | 26.7294 | 0.8786 | 0.6192 |
| GZ90 | 30639724 | 2841737 | 0.0927 | 27797987 | 27204142 | 0.9786 | 6.2191 | 26.7609 | 0.8719 | 0.5548 |
| GZ91 | 54287882 | 7051135 | 0.1299 | 47236747 | 46220362 | 0.9785 | 11.0351 | 26.4425 | 0.8848 | 0.7724 |
| GZ92 | 31980572 | 3517150 | 0.11 | 28463422 | 27870426 | 0.9792 | 6.4945 | 26.7124 | 0.8737 | 0.5712 |
| GZ93 | 46792616 | 5695907 | 0.1217 | 41096709 | 40209238 | 0.9784 | 9.4985 | 26.7059 | 0.886 | 0.7415 |
| GZ94 | 46077988 | 6052047 | 0.1313 | 40025941 | 39191061 | 0.9791 | 9.3786 | 26.6774 | 0.8861 | 0.7463 |
| GZ95 | 32373840 | 3109987 | 0.0961 | 29263853 | 28629596 | 0.9783 | 6.5693 | 26.5638 | 0.8731 | 0.5816 |
| GZ96 | 37098786 | 4107163 | 0.1107 | 32991623 | 31952975 | 0.9685 | 7.4555 | 26.6397 | 0.8782 | 0.644 |
| GZ97 | 28319652 | 2596608 | 0.0917 | 25723044 | 25145386 | 0.9775 | 5.7331 | 26.832 | 0.8681 | 0.4963 |
| GZ98 | 34869402 | 3872916 | 0.1111 | 30996486 | 30234391 | 0.9754 | 7.0637 | 26.7657 | 0.878 | 0.6265 |
| GZ99 | 47905134 | 5795390 | 0.121 | 42109744 | 41292349 | 0.9806 | 9.7826 | 26.8381 | 0.8949 | 0.7747 |
| HYZ | 303684444 | 45039052 | 0.1483 | 258645392 | 254564831 | 0.9842 | 62.3786 | 26.96 | 0.915 | 0.8959 |
| J101 | 33087154 | 3901675 | 0.1179 | 29185479 | 27479061 | 0.9415 | 6.514 | 28.3355 | 0.8873 | 0.5711 |
| MHMD | 27117974 | 2030971 | 0.0749 | 25087003 | 24662900 | 0.9831 | 5.5843 | 27.1184 | 0.8758 | 0.5369 |
| SL105 | 34330736 | 7683689 | 0.2238 | 26647047 | 25561062 | 0.9592 | 6.8805 | 25.3547 | 0.8427 | 0.5533 |
| SL108 | 30249748 | 3845439 | 0.1271 | 26404309 | 24021119 | 0.9097 | 5.7936 | 26.2419 | 0.8547 | 0.5115 |
| SL109 | 29896494 | 6565454 | 0.2196 | 23331040 | 22445899 | 0.9621 | 5.9653 | 25.9698 | 0.8276 | 0.4863 |
| SL12 | 32661690 | 7310380 | 0.2238 | 25351310 | 24346053 | 0.9603 | 6.3928 | 24.0057 | 0.7871 | 0.4862 |
| SL129 | 32378208 | 4149655 | 0.1282 | 28228553 | 25771394 | 0.913 | 6.2173 | 25.8304 | 0.8581 | 0.5432 |
| SL130 | 29150356 | 3543793 | 0.1216 | 25606563 | 23693081 | 0.9253 | 5.6659 | 25.5281 | 0.8547 | 0.4939 |
| SL136 | 32431410 | 6884838 | 0.2123 | 25546572 | 24096145 | 0.9432 | 6.3022 | 22.5739 | 0.7759 | 0.4715 |
| SL137 | 32660054 | 7249567 | 0.222 | 25410487 | 24406933 | 0.9605 | 6.4365 | 22.3982 | 0.7736 | 0.4781 |
| SL138 | 31078744 | 3954524 | 0.1272 | 27124220 | 25390474 | 0.9361 | 6.0904 | 25.9877 | 0.8558 | 0.5231 |
| SL140 | 29254024 | 3803837 | 0.13 | 25450187 | 23639224 | 0.9288 | 5.7095 | 25.4985 | 0.845 | 0.5019 |
| SL141 | 22600874 | 2420456 | 0.1071 | 20180418 | 17803854 | 0.8822 | 4.2257 | 25.327 | 0.82 | 0.3712 |
| SL19 | 26639332 | 5853541 | 0.2197 | 20785791 | 19462645 | 0.9363 | 5.1631 | 25.2305 | 0.7914 | 0.413 |
| SL25 | 36405356 | 8142712 | 0.2237 | 28262644 | 26555636 | 0.9396 | 7.1194 | 22.7551 | 0.7893 | 0.5355 |
| SL32 | 29767806 | 6152456 | 0.2067 | 23615350 | 22025756 | 0.9327 | 5.698 | 19.8055 | 0.7374 | 0.4092 |
| SL35 | 29810078 | 6850500 | 0.2298 | 22959578 | 21274187 | 0.9266 | 5.8017 | 22.5932 | 0.7607 | 0.4409 |
| SL36 | 29463634 | 6636481 | 0.2252 | 22827153 | 21999263 | 0.9637 | 5.7873 | 23.1457 | 0.757 | 0.4304 |
| SL37 | 32026616 | 7094285 | 0.2215 | 24932331 | 23649520 | 0.9485 | 6.2236 | 22.5885 | 0.768 | 0.4646 |
| SL39 | 32759328 | 7471633 | 0.2281 | 25287695 | 24092167 | 0.9527 | 6.5584 | 25.9493 | 0.8454 | 0.5457 |
| SL41 | 32598884 | 7194570 | 0.2207 | 25404314 | 24077151 | 0.9478 | 6.479 | 24.5819 | 0.8282 | 0.5273 |
| SL43 | 28966022 | 6524975 | 0.2253 | 22441047 | 20900577 | 0.9314 | 5.5991 | 22.7133 | 0.7647 | 0.4275 |
| SL47 | 34810438 | 7662526 | 0.2201 | 27147912 | 26105916 | 0.9616 | 6.9205 | 24.7291 | 0.8379 | 0.5496 |
| SL49 | 28752730 | 3607056 | 0.1255 | 25145674 | 23273026 | 0.9255 | 5.553 | 23.0778 | 0.7878 | 0.455 |
| SL51 | 29566470 | 6471726 | 0.2189 | 23094744 | 22008019 | 0.9529 | 5.8369 | 23.8228 | 0.7877 | 0.4558 |
| SL53 | 32998688 | 7292944 | 0.221 | 25705744 | 24225892 | 0.9424 | 6.5259 | 25.1758 | 0.8395 | 0.5313 |
| SL62 | 32736226 | 7207737 | 0.2202 | 25528489 | 24556438 | 0.9619 | 6.5082 | 23.5026 | 0.793 | 0.4947 |
| SL83 | 31528138 | 6638913 | 0.2106 | 24889225 | 24045398 | 0.9661 | 6.3005 | 25.1811 | 0.8292 | 0.5132 |
| SL97 | 31995438 | 6895197 | 0.2155 | 25100241 | 23856927 | 0.9505 | 6.2957 | 24.1113 | 0.8 | 0.4899 |
| Tu14 | 35999268 | 7635202 | 0.2121 | 28364066 | 26173080 | 0.9228 | 6.6448 | 23.9624 | 0.8007 | 0.5218 |
| Tu30 | 29208960 | 3103308 | 0.1062 | 26105652 | 24360185 | 0.9331 | 5.5669 | 23.9001 | 0.7928 | 0.4564 |
| Tu5 | 34443956 | 7545380 | 0.2191 | 26898576 | 25824869 | 0.9601 | 6.8556 | 24.7736 | 0.8444 | 0.5501 |
| WSC100 | 29027160 | 3719916 | 0.1282 | 25307244 | 24694382 | 0.9758 | 5.9155 | 27.1304 | 0.8736 | 0.537 |
| WSC104 | 30181968 | 2655279 | 0.088 | 27526689 | 26974592 | 0.9799 | 6.193 | 27.8472 | 0.8954 | 0.6038 |
| WSC105 | 29375624 | 3755371 | 0.1278 | 25620253 | 24953998 | 0.974 | 5.9715 | 27.7445 | 0.8868 | 0.5434 |
| WSC12 | 35334598 | 4813967 | 0.1362 | 30520631 | 29644850 | 0.9713 | 7.18 | 26.7487 | 0.8793 | 0.6466 |
| WSC13 | 35819102 | 5040994 | 0.1407 | 30778108 | 30008410 | 0.975 | 7.2931 | 26.8574 | 0.8791 | 0.6503 |
| WSC15 | 29150904 | 3177026 | 0.109 | 25973878 | 25544649 | 0.9835 | 5.9611 | 26.6492 | 0.8703 | 0.5444 |
| WSC16 | 26211322 | 3108074 | 0.1186 | 23103248 | 22578560 | 0.9773 | 5.3424 | 26.6871 | 0.8626 | 0.4827 |
| WSC17 | 30709000 | 4400396 | 0.1433 | 26308604 | 25573833 | 0.9721 | 6.2053 | 26.8139 | 0.87 | 0.5417 |
| WSC18 | 29565736 | 4061862 | 0.1374 | 25503874 | 24949709 | 0.9783 | 6.0515 | 27.9285 | 0.8896 | 0.5715 |
| WSC29 | 28774526 | 3393571 | 0.1179 | 25380955 | 24925276 | 0.982 | 5.9153 | 26.7253 | 0.8723 | 0.5537 |
| WSC30 | 23750806 | 3079478 | 0.1297 | 20671328 | 20189291 | 0.9767 | 4.8412 | 26.3774 | 0.8467 | 0.4088 |
| WSC31 | 29204002 | 3045314 | 0.1043 | 26158688 | 25430620 | 0.9722 | 5.931 | 26.5306 | 0.8654 | 0.5353 |
| WSC32 | 27333516 | 2998573 | 0.1097 | 24334943 | 23717397 | 0.9746 | 5.5743 | 27.3894 | 0.8742 | 0.5251 |
| WSC34 | 28848128 | 3335431 | 0.1156 | 25512697 | 24987002 | 0.9794 | 5.9088 | 26.8155 | 0.8686 | 0.5507 |
| WSC38 | 24560054 | 2943676 | 0.1199 | 21616378 | 21102148 | 0.9762 | 4.9813 | 26.8379 | 0.8554 | 0.4205 |
| WSC40 | 31343098 | 3717903 | 0.1186 | 27625195 | 26894651 | 0.9736 | 6.3471 | 26.8819 | 0.8738 | 0.5599 |
| WSC52 | 27392390 | 2897171 | 0.1058 | 24495219 | 23913770 | 0.9763 | 5.5559 | 26.5197 | 0.8613 | 0.4959 |
| WSC54 | 34540674 | 3665593 | 0.1061 | 30875081 | 29879405 | 0.9678 | 7.0114 | 27.895 | 0.8992 | 0.6632 |
| WSC56 | 34038800 | 5407098 | 0.1589 | 28631702 | 27757103 | 0.9695 | 6.8972 | 26.6926 | 0.8822 | 0.603 |
| WSC59 | 33889912 | 5571816 | 0.1644 | 28318096 | 27547777 | 0.9728 | 6.9015 | 27.1183 | 0.8839 | 0.6175 |
| WSC61 | 29196938 | 4258871 | 0.1459 | 24938067 | 24419273 | 0.9792 | 5.9877 | 27.5245 | 0.8829 | 0.5546 |
| WSC68 | 29373516 | 5005303 | 0.1704 | 24368213 | 23835700 | 0.9781 | 6.0163 | 27.8462 | 0.8879 | 0.5513 |
| WSC70 | 29349330 | 3582965 | 0.1221 | 25766365 | 25159632 | 0.9765 | 5.9819 | 28.5181 | 0.8954 | 0.5567 |
| WSC72 | 26472082 | 3799547 | 0.1435 | 22672535 | 22206141 | 0.9794 | 5.425 | 28.3704 | 0.8858 | 0.4943 |
| WSC73 | 31861992 | 3909715 | 0.1227 | 27952277 | 26928962 | 0.9634 | 6.4333 | 27.9038 | 0.8962 | 0.6107 |
| WSC79 | 27703720 | 3600359 | 0.13 | 24103361 | 23598466 | 0.9791 | 5.653 | 27.0725 | 0.8709 | 0.4808 |
| WSC81 | 36360598 | 4488666 | 0.1234 | 31871932 | 31156252 | 0.9775 | 7.4395 | 27.6807 | 0.8903 | 0.6811 |
| WSC85 | 23495708 | 2101651 | 0.0894 | 21394057 | 20933428 | 0.9785 | 4.8138 | 27.1543 | 0.8656 | 0.4427 |
| WSC93 | 23969674 | 3056038 | 0.1275 | 20913636 | 20438476 | 0.9773 | 4.9005 | 27.0525 | 0.8598 | 0.4313 |
| WSC94 | 31735972 | 4176493 | 0.1316 | 27559479 | 27089315 | 0.9829 | 6.5256 | 27.2545 | 0.8827 | 0.6017 |
| WSC98 | 32389548 | 5477252 | 0.1691 | 26912296 | 26291280 | 0.9769 | 6.6169 | 27.0462 | 0.8743 | 0.5921 |
| WSC99 | 25325752 | 3214582 | 0.1269 | 22111170 | 21514092 | 0.973 | 5.1557 | 27.4322 | 0.8682 | 0.4607 |

**Supplemental Table 3.** Putative annotation of the top 1‰ of the domestication genes identified by three methods

| **Metric** | **Between Pops / Among pop** | **Chrom** | **BIN_start** | **BIN_end** | **Gene ID** | **Homologous species** | **Orthologue gene** | **Similarity** | **Character / Function** | **Trait** |
| --- | --- | --- | --- | --- | --- | --- | --- | --- | --- | --- |
| F_ST_ | South-North | Chr01 | 73510001 | 73520000 | Sobic.001G459500 | *Oryza sativa* | *9RKe; NRKe* | 0.435 | temperature tolerance | abiotic stress resistance |
| F_ST_ | South-North | Chr01 | 73650001 | 73660000 | Sobic.001G461900 | *Oryza sativa* | *GOT1B; glup2; gpa4* | 0.993 | powdery endosperm, protein content,1000-grain weight | yield, grain quality |
| F_ST_ | South-North | Chr01 | 73670001 | 73680000 | Sobic.001G462200 | *Oryza sativa* | *OsMPG1; OsVTC1-1* | 0.518 | AsA synthesis/ salt tolerance | abiotic stress resistance |
| F_ST_ | South-North | Chr01 | 73670001 | 73680000 | Sobic.001G462300 | *Oryza sativa* | *CycA3;2* | 0.525 | mitotic cycle |  |
| F_ST_ | South-North | Chr01 | 73850001 | 73860000 | Sobic.001G464500 | *Oryza sativa* | *SPL35* | 0.563 | similar disease spot/disease resistance | biotic stress resistance |
| F_ST_ | South-Chishui | Chr01 | 78100001 | 78110000 | Sobic.001G513700 | *Oryza sativa* | *RAI1* | 0.804 | rice blast resistance | biotic stress resistance |
| F_ST_ | South-Chishui | Chr02 | 16900001 | 16910000 | Sobic.002G125300 | *Oryza sativa* | *OsPIP2;4* | 0.962 | boron toxicity tolerance | adverse tolerance |
| F_ST_ | South-North | Chr02 | 55030001 | 55040000 | Sobic.002G174000 | *Oryza sativa* | *RSS2; OsPDR12* | 0.607 | salt tolerance | abiotic stress resistance |
| F_ST_ | South-North | Chr03 | 430001 | 440000 | Sobic.003G004500 | *Oryza sativa* | *OsMYL2* | 0.9 | cherry blossom synthetic/disease resistance | biotic stress resistance |
| F_ST_ | South-North | Chr03 | 4370001 | 4380000 | Sobic.003G047900 | *Oryza sativa* | *OsBOR4* | 0.848 | pollen development/floral organ development | pollen fertility |
| F_ST_ | South-North | Chr03 | 4440001 | 4450000 | Sobic.003G048200 | *Oryza sativa* | *CYP734A2* | 0.813 | dwarf stem, small grains | yeild |
| F_ST_ | South-Chishui | Chr03 | 4470001 | 4480000 | Sobic.003G048500 | *Zea mays* | *mrpa7* | 0.98 | anthocyanin synthesis |  |
| F_ST_ | South-Chishui | Chr03 | 4470001 | 4480000 | Sobic.003G048600 | *Oryza sativa* | *OsGRX5* | 0.897 | environmental stresses | abiotic stress resistance |
| F_ST_ | South-North | Chr03 | 4520001 | 4530000 | Sobic.003G049500 | *Oryza sativa* | *OsMRP5* | 0.688 | seed weight | yield |
| F_ST_ | South-North | Chr03 | 4540001 | 4550000 | Sobic.003G049600 | *Oryza sativa* | *OsCesA9* | 0.785 | secondary cell wall synthesis, plant height, partial male sterility | plant shape,pollen fertility |
| F_ST_ | South-Chishui | Chr06 | 7190001 | 7200000 | Sobic.006G032600 | *Oryza sativa* | *OsRMR1* | 0.567 | storage protein synthesis | grain quality |
| F_ST_ | South-North | Chr06 | 55500001 | 55510000 | Sobic.006G205100 | *Oryza sativa* | *OsRMT1* | 0.84 | salt tolerance | abiotic stress resistance |
| F_ST_ | South-North | Chr07 | 58380001 | 58390000 | Sobic.007G151500 | *Oryza sativa* | *OsBRXL4* | 0.807 | tillering angle | plant shape |
| F_ST_ | South-North | Chr09 | 3060001 | 3070000 | Sobic.009G033800 | *Oryza sativa* | *OsIFL* | 0.404 | salt tolerance | abiotic stress resistance |
| F_ST_ | South-North | Chr09 | 3080001 | 3090000 | Sobic.009G034200 | *Oryza sativa* | *OsCIPK23* | 0.67 | potassium absorption, growth and development | growth and development |
| F_ST_ | South-Chishui | Chr09 | 46180001 | 46190000 | Sobic.009G117400 | *Oryza sativa* | *OsRLCK185* | 0.922 | bacterial leaf blight resistance | biotic stress resistance |
| F_ST_ | South-Chishui | Chr09 | 47740001 | 47750000 | Sobic.009G124200 | *Oryza sativa* | SMOS1 | 0.663 | auxin-dependent regulator for cell expansion | organ size |
| F_ST_ | South-Chishui | Chr09 | 53480001 | 53490000 | Sobic.009G180600 | *Oryza sativa* | *OsCPK26* | 0.865 | stamens number | pollen fertility |
| F_ST_ | South-Chishui | Chr09 | 58640001 | 58650000 | Sobic.009G251400 | *Zea mays* | *mrpa7* | 0.823 | multi-resistant related proteins | anthocyanin synthesis |
| Pi | South-Chishui | Chr01 | 56290001 | 56300000 | Sobic.001G287400 | *Zea mays* | *vq5* | 0.832 | drought and osmotic tolerance | abiotic stress resistance |
| Pi | South-North | Chr01 | 66790001 | 66800000 | Sobic.001G379600 | *Oryza sativa* | *OsCIPK15* | 0.728 | disease resistance , water stress | biotic/abiotic stress resistance |
| Pi | South-North | Chr01 | 73480001 | 73490000 | Sobic.001G458600 | *Oryza sativa* | *Os4BGlu13* | 0.679 | root growth | growth and development |
| Pi | South-North | Chr01 | 73690001 | 73700000 | Sobic.001G462500 | *Oryza sativa* | *NRAT1* | 0.56 | aluminum tolerance | abiotic stress resistance |
| Pi | South-North | Chr01 | 73890001 | 73900000 | Sobic.001G465100 | *Oryza sativa* | *HTD2; D88; D14* | 0.812 | tiller number | plant shape |
| Pi | South-North | Chr01 | 73950001 | 73960000 | Sobic.001G465800 | *Oryza sativa* | *OsICS1* | 0.64 | salicylic acid biosynthesis/disease resistance | yield |
| Pi | South-Chishui | Chr02 | 4930001 | 4940000 | Sobic.002G051900 | *Oryza sativa* | *OsHsfA2b; OsHSF5* | 0.884 | environment (hot) stress | abiotic stress resistance |
| Pi | South-North | Chr02 | 9370001 | 9380000 | Sobic.002G089400 | *Oryza sativa* | *CAL1* | 0.595 | cadmium tolerance | abiotic stress resistance |
| Pi | South-North | Chr02 | 9370001 | 9380000 | Sobic.002G089500 | *Oryza sativa* | *CAL1* | 0.615 | cadmium tolerance | abiotic stress resistance |
| Pi | South-North | Chr03 | 390001 | 400000 | Sobic.003G003800 | *Oryza sativa* | *OsARF1* | 0.862 | auxin response factor/heading stage, plant height, disease resistance, panicle | photoperiod, plant shape, disease resistance |
| Pi | South-North | Chr03 | 12930001 | 12940000 | Sobic.003G136100 | *Oryza sativa* | *OsCCR1* | 0.774 | disease resistance | adverse tolerance |
| Pi | South-North | Chr03 | 12980001 | 12990000 | Sobic.003G136400 | *Oryza sativa* | *OsCYP20-2* | 0.74 | environmental stresses | abiotic stress resistance |
| Pi | South-Chishui | Chr03 | 56230001 | 56240000 | Sobic.003G225900 | *Oryza sativa* | *OsGAP1* | 0.958 | bacterial leaf blight resistance | biotic stress resistance |
| Pi | South-Chishui | Chr03 | 56280001 | 56290000 | Sobic.003G226500 | *Oryza sativa* | *OsSGT1* | 0.929 | bacterial leaf blight resistance | biotic stress resistance |
| Pi | South-Chishui | Chr03 | 57490001 | 57500000 | Sobic.003G236300 | *Arabidopsis thaliana* | *ACBP1* |  | response to lead ion | abiotic stress resistance |
| Pi | South-Chishui | Chr03 | 59990001 | 60000000 | Sobic.003G262200 | *Arabidopsis thaliana* | *GRV2* |  | hypocotyl and stems | starch synthesis |
| Pi | South-Chishui | Chr04 | 57550001 | 57560000 | Sobic.004G225200 | *Arabidopsis thaliana* | *ATNIT4/NIT4* | 0.803 | cyanide detoxification | cyanide synthesis |
| Pi | South-North | Chr04 | 62800001 | 62810000 | Sobic.004G285400 | *Oryza sativa* | *SID1* | 0.724 | flowering time/photoperiod | photoperiod |
| Pi | South-North | Chr04 | 62830001 | 62840000 | Sobic.004G285900 | *Zea mays* | *hmg2* | 0.955 | high mobility group box protein 2 |  |
| Pi | South-Chishui | Chr05 | 420001 | 430000 | Sobic.005G005400 | *Zea mays* | *mate5* | 0.797 | herbicide tolerance (glyphosate) | herbicide tolerance |
| Pi | South-North | Chr05 | 61040001 | 61050000 | Sobic.005G145201 | *Oryza sativa* | *GF14f* | 0.898 | grain | yield |
| Pi | South-North | Chr05 | 61040001 | 61050000 | Sobic.005G145300 | *Oryza sativa* | *OsFKF1* | 0.929 | flowering time/photoperiod | photoperiod |
| Pi | South-North | Chr06 | 870001 | 880000 | Sobic.006G005600 | *Oryza sativa* | *OsHSP1* | 0.932 | heat tolerance | acclimatization |
| Pi | South-Chishui | Chr07 | 2100001 | 2110000 | Sobic.007G023400 | *Zea mays* | *sudh7* | 0.972 | seed germination | growth and development |
| Pi | South-North | Chr09 | 50450001 | 50460000 | Sobic.009G147500 | *Zea mays* | *kch2* | 0.918 | potassium ions | nutrients utilization |
| Pi | South-Chishui | Chr09 | 55730001 | 55740000 | Sobic.009G211200 | *Oryza sativa* | *AET1* | 0.871 | auxin response and environmental temperature adaptation | abiotic stress resistance |
| Pi | South-North | Chr10 | 4350001 | 4360000 | Sobic.010G056000 | *Oryza sativa* | *OsGBP3* | 0.698 | grain size/plant height | yield、plant shape |
| Pi | South-North | Chr10 | 4370001 | 4380000 | Sobic.010G056200 | *Oryza sativa* | *SRWD3* | 0.902 | salt tolerance | abiotic stress resistance |
| Pi | South-North | Chr10 | 4450001 | 4460000 | Sobic.010G057000 | *Oryza sativa* | *OsCML30* | 0.759 | calmodulin |  |
| Tajima'D | North | Chr01 | 15870001 | 15880000 | Sobic.001G185500 | *Oryza sativa* | *PEZ1* | 0.886 | transport PCA/increase PCA | nutrients utilization |
| Tajima'D | North | Chr01 | 15870001 | 15880000 | Sobic.001G185600 | *Oryza sativa* | *PEZ1* | 0.809 | transport PCA/increase PCA | nutrients utilization |
| Tajima'D | South | Chr01 | 64730001 | 64740000 | Sobic.001G357500 | *Oryza sativa* | *OsPUT1* | 0.599 | development, biological and abiotic stress | biotic/abiotic stress resistance |
| Tajima'D | South | Chr01 | 64730001 | 64740000 | Sobic.001G357600 | *Oryza sativa* | *OsPUT1* | 0.639 | development, biological and abiotic stress | biotic/abiotic stress resistance |
| Tajima'D | North | Chr01 | 68780001 | 68790000 | Sobic.001G402300 | *Oryza sativa* | *SRS3 OsKinesin-13A* | 0.734 | seed length/size of the caryopsis | yield |
| Tajima'D | North | Chr01 | 73040001 | 73050000 | Sobic.001G454000 | *Oryza sativa* | *OsHSP71.1* | 0.432 | heat tolerance | adverse tolerance |
| Tajima'D | North | Chr02 | 13110001 | 13120000 | Sobic.002G108400 | *Oryza sativa* | *CYP76M7* | 0.507 | antifungal hormone | biotic stress resistance |
| Tajima'D | South | Chr02 | 17980001 | 17990000 | Sobic.002G128900 | *Oryza sativa* | *OsACOT* | 0.424 | grain shape/grain weight | yield |
| Tajima'D | South | Chr02 | 55780001 | 55790000 | Sobic.002G177700 | *Zea mays* | *hat1* | 0.931 | salt tolerance ,drought tolerance | adverse tolerance |
| Tajima'D | Chishui | Chr02 | 62000001 | 62010000 | Sobic.002G228800 | *Oryza sativa* | *OsCEP6.1* | 0.511 | plant height, lower amylose content, spike length, grain yield, branch number at a time | plant height, yield,grain quality |
| Tajima'D | Chishui | Chr02 | 62040001 | 62050000 | Sobic.002G229300 | *Oryza sativa  Zea mays* | *GS5  GRMZM2G072240* | 0.511 0.769 | grain size in rice   formation of the number of husk layers in maize | Yield |
| Tajima'D | North | Chr02 | 70660001 | 70670000 | Sobic.002G339500 | *Oryza sativa* | *OsLCBK1* | 0.345 | resistance/programmed cell death | biotic stress resistance |
| Tajima'D | North | Chr03 | 9950001 | 9960000 | Sobic.003G110400 | *Zea mays* | *nrh1* | 1 |  |  |
| Tajima'D | North | Chr03 | 57210001 | 57220000 | Sobic.003G233200 | *Oryza sativa* | *Snl6* | 0.957 | bacterial leaf blight resistance | biotic stress resistance |
| Tajima'D | South | Chr03 | 57850001 | 57860000 | Sobic.003G238900 | *Oryza sativa* | *BS1* | 0.529 | cell wall synthesis | growth and development |
| Tajima'D | North | Chr03 | 71020001 | 71030000 | Sobic.003G402000 | *Oryza sativa* | *C3H12* | 0.89 | bacterial leaf blight resistance | biotic stress resistance |
| Tajima'D | North | Chr04 | 8720001 | 8730000 | Sobic.004G098100 | *Oryza sativa* | *OsO3L3* | 0.544 | cadmium accumulation | adverse tolerance |
| Tajima'D | North | Chr04 | 57750001 | 57760000 | Sobic.004G227900 | *Oryza sativa* | *OsEXPB5* | 0.553 | root hair | growth and development |
| Tajima'D | Chishui | Chr05 | 15930001 | 15940000 | Sobic.005G098200 | *Oryza sativa* | *OsCR4* | 0.434 | between the inner and outer glume occlusion/sterility | pollen fertility |
| Tajima'D | Chishui | Chr05 | 15930001 | 15940000 | Sobic.005G098100 | *Oryza sativa* | *DP1* | 0.216 | floral organ | yield |
| Tajima'D | Chishui | Chr05 | 18820001 | 18830000 | Sobic.005G104700 | *Zea mays* | *yuc6* | 0.67 | roots/salt tolerance | nutrients utilization, resistance abiotic |
| Tajima'D | Chishui | Chr05 | 19790001 | 19800000 | Sobic.005G105700 | *Oryza sativa* | *D10 OsCCD8* | 0.689 | tiller number | plant shape |
| Tajima'D | Chishui | Chr05 | 55270001 | 55280000 | Sobic.005G127200 | *Oryza sativa* | *BS1* | 0.522 | participate in the regulation of secondary wall formation | growth and development |
| Tajima'D | Chishui | Chr05 | 55290001 | 55300000 | Sobic.005G127300 | *Oryza sativa* | *CRD1* | 0.236 | plant height, tillering, spikelet fertility | growth and development，pollen fertility |
| Tajima'D | Chishui | Chr05 | 55590001 | 55600000 | Sobic.005G128000 | *Oryza sativa* | *NLS1* | 0.59 | leaf sheath necrosis | biotic stress resistance |
| Tajima'D | North | Chr05 | 62390001 | 62400000 | Sobic.005G153100 | *Zea mays* | *esr6* | 0.615 | environmental stresses | adverse tolerance |
| Tajima'D | North | Chr06 | 170001 | 180000 | Sobic.006G001200 | *Oryza sativa* | *SOR1* | 0.743 | root system | growth and development |
| Tajima'D | Chishui | Chr06 | 46390001 | 46400000 | Sobic.006G094201 | *Oryza sativa* | *ZEP1* | 0.418 | pollen abortion | pollen fertility |
| Tajima'D | North | Chr06 | 49460001 | 49470000 | Sobic.006G130400 | *Oryza sativa* | *OsNPF4.5* | 0.538 | nutrient elements (N) absorption | nutrients utilization, growth and development |
| Tajima'D | North | Chr06 | 49620001 | 49630000 | Sobic.006G132100 | *Oryza sativa* | *OsCHI11* | 0.539 | sheath blight resistance | biotic stress resistance |
| Tajima'D | North | Chr06 | 49620001 | 49630000 | Sobic.006G132000 | *Oryza sativa* | *OsDG2* | 0.742 | regulation of chloroplast | growth and development |
| Tajima'D | South | Chr06 | 59280001 | 59290000 | Sobic.006G255500 | *Oryza sativa* | *OSINV4* | 0.638 | anther development | pollen fertility |
| Tajima'D | South | Chr06 | 59280001 | 59290000 | Sobic.006G255600 | *Zea mays* | *incw4* | 0.867 | regulating aging/keeping leaf green | growth and development |
| Tajima'D | North | Chr08 | 5400001 | 5410000 | Sobic.008G053600 | *Oryza sativa* | *FucT* | 0.359 | rice blast resistance | biotic stress resistance |
| Tajima'D | Chishui | Chr08 | 51660001 | 51670000 | Sobic.008G111300 | *Oryza sativa* | *OsTMT2* | 0.501 | monosaccharide transporters | biotic stress resistance |
| Tajima'D | Chishui | Chr08 | 52390001 | 52400000 | Sobic.008G115000 | *Oryza sativa* | *OsVIL2* | 0.835 | heading stage | photoperiod |
| Tajima'D | Chishui | Chr08 | 53080001 | 53090000 | Sobic.008G118400 | *Oryza sativa* | *OsACBP6* | 0.705 | lipid metabolism | plant height，panicle shape |
| Tajima'D | North | Chr08 | 62460001 | 62470000 | Sobic.008G190500 | *Oryza sativa* | *OSA7* | 0.914 | nutrient uptake |  |
| Tajima'D | Chishui | Chr09 | 2630001 | 2640000 | Sobic.009G029500 | *Oryza sativa* | *OZT1* | 0.907 | zinc sensitivity | nutrients utilization |
| Tajima'D | South | Chr09 | 4940001 | 4950000 | Sobic.009G049600 | *Oryza sativa* | *ASP1 OsREL2* | 0.336 | panicle /branch | panicle shape |
| Tajima'D | South | Chr09 | 8500001 | 8510000 | Sobic.009G071500 | *Oryza sativa* | *SNAC3* | 0.644 | environment (temperature, drought and penetration tolerance) stress | adverse tolerance |
| Tajima'D | North | Chr09 | 49360001 | 49370000 | Sobic.009G137000 | *Oryza sativa* | *TCD5* | 0.874 | cold tolerance | adverse tolerance |
| Tajima'D | Chishui | Chr09 | 56180001 | 56190000 | Sobic.009G217500 | *Oryza sativa* | *SMG2 OsMKKK10* | 0.325 | plant height, spike length, grain size | plant height, yield |
| Tajima'D | South | Chr10 | 19400001 | 19410000 | Sobic.010G133300 | *Oryza sativa* | *OsPT10* | 0.85 | adjust the element (P) | nutrients utilization |
| Tajima'D | South | Chr10 | 20620001 | 20630000 | Sobic.010G136300 | *Oryza sativa* | *OsMFT1* | 0.873 | flowering/panicle | photoperiod，panicle shape |
| Tajima'D | South | Chr10 | 41740001 | 41750000 | Sobic.010G146500 | *Oryza sativa* | *NLS1* | 0.54 | leaf sheath necrosis/disease resistance | biotic stress resistance |
| Tajima'D | Chishui | Chr10 | 55440001 | 55450000 | Sobic.010G211400 | *Oryza sativa* | *OsRCI2-5* | 0.579 | drought tolerance | adverse tolerance |
| Tajima'D | North | Chr10 | 56190001 | 56200000 | Sobic.010G219700 | *Oryza sativa* | *MOC2 FBP1* | 0.389 | tiller number | plant shape |

**Supplemental Table 4** Statistical analysis of phenotypic data of 7 traits in 244 Chinese sorghums in different environments

Note: TGW - Thousand Grain Weight, GL - Grain Length, GW - Grain width, PT-Pericarp Thickness, TT - Testa Thickness

| **Traits** | **Environment** | **Unit** | **Minimum** | **Maximum** | **Mean** | **Median** | **Std. Deviation** | **Std. Error of Mean** |
| --- | --- | --- | --- | --- | --- | --- | --- | --- |
| TGW | 2018GY | g | 13.75 | 43.25 | 24.75 | 23.93 | 4.68 | 0.302 |
| TGW | 2019GY | g | 12.67 | 42.65 | 25.07 | 24.42 | 4.96 | 0.318 |
| TGW | 2020GY | g | 10.74 | 37.81 | 22.23 | 21.67 | 4.95 | 0.317 |
| TGW | 2019HZ | g | 7.00 | 38.96 | 21.87 | 21.52 | 5.41 | 0.370 |
| TGW | 2020LD | g | 10.08 | 45.64 | 25.44 | 25.36 | 4.94 | 0.316 |
| TGW | 2020LS | g | 16.84 | 40.48 | 26.90 | 26.82 | 5.06 | 0.331 |
| GL | 2018GY | mm | 3.35 | 5.57 | 4.11 | 4.07 | 0.41 | 0.027 |
| GL | 2019GY | mm | 2.55 | 4.14 | 3.30 | 3.26 | 0.34 | 0.022 |
| GL | 2020GY | mm | 3.14 | 5.47 | 4.27 | 4.27 | 0.46 | 0.030 |
| GL | 2019HZ | mm | 3.42 | 5.68 | 4.34 | 4.31 | 0.47 | 0.032 |
| GL | 2020LD | mm | 3.66 | 5.65 | 4.54 | 4.52 | 0.42 | 0.027 |
| GL | 2020LS | mm | 3.58 | 5.71 | 4.51 | 4.52 | 0.45 | 0.029 |
| GW | 2018GY | mm | 2.50 | 3.99 | 3.25 | 3.23 | 0.30 | 0.019 |
| GW | 2019GY | mm | 2.02 | 3.17 | 2.59 | 2.59 | 0.19 | 0.012 |
| GW | 2020GY | mm | 2.58 | 4.41 | 3.36 | 3.34 | 0.29 | 0.019 |
| GW | 2019HZ | mm | 2.21 | 3.87 | 3.17 | 3.18 | 0.28 | 0.019 |
| GW | 2020LD | mm | 2.59 | 4.30 | 3.43 | 3.40 | 0.27 | 0.017 |
| GW | 2020LS | mm | 2.84 | 4.39 | 3.48 | 3.47 | 0.29 | 0.019 |
| TT | 2020LD | um | 0.00 | 45.33 | 13.51 | 12.61 | 7.87 | 0.505 |
| PT | 2020LD | um | 7.13 | 147.87 | 42.34 | 38.35 | 22.38 | 1.436 |

**Supplemental Table 5** GWAS results of grain physical properties related to liquor brewing process in 244 Chinese accessions under one to six environments

Note: TGW - Thousand Grain Weight, GW - Grain width, PT - Pericarp Thickness, TT - Testa Thickness

| **Traits** | **Environments** | **Chrom** | **Position** | **P.value** | **-LogP** | **maf** | **Effect** | **Model** |
| --- | --- | --- | --- | --- | --- | --- | --- | --- |
| GW | 2018HZ | Chr10 | 1893514 | 2.67E-09 | 8.57 | 0.07 | -0.28 | GLM |
| GW | 2018HZ | Chr10 | 1893766 | 5.50E-09 | 8.26 | 0.08 | -0.27 | GLM |
| GW | 2018HZ | Chr10 | 1892495 | 1.34E-08 | 7.87 | 0.09 | -0.26 | GLM |
| GW | 2018HZ | Chr10 | 1892303 | 1.89E-08 | 7.72 | 0.09 | -0.26 | GLM |
| GW | 2020LS | Chr08 | 12894094 | 2.59E-08 | 7.59 | 0.07 | -0.34 | GLM |
| GW | 2020GY | Chr09 | 47453177 | 2.26E-13 | 12.65 | 0.09 | NA | Blink |
| GW | 2020GY | Chr10 | 11206192 | 2.02E-12 | 11.69 | 0.21 | NA | Blink |
| TGW | 2020LD | Chr02 | 7584046 | 3.09E-11 | 10.51 | 0.08 | NA | Blink |
| TGW | 2020LD | Chr02 | 7584046 | 2.21E-08 | 7.66 | 0.08 | -3.42 | GLM |
| TGW | 2018GY | Chr03 | 53801086 | 9.56E-09 | 8.02 | 0.21 | NA | Blink |
| TGW | 2018HZ | Chr05 | 19065356 | 3.84E-09 | 8.42 | 0.26 | NA | Blink |
| TGW | 2020LS | Chr05 | 65881442 | 3.03E-09 | 8.52 | 0.15 | NA | Blink |
| TGW | 2019GY | Chr05 | 70018996 | 3.14E-11 | 10.50 | 0.09 | NA | Blink |
| TGW | 2020LS | Chr08 | 12894094 | 1.27E-11 | 10.90 | 0.07 | NA | Blink |
| TGW | 2020GY | Chr09 | 47428379 | 3.95E-08 | 7.40 | 0.10 | -2.98 | GLM |
| TGW | 2020GY | Chr09 | 47428533 | 4.24E-08 | 7.37 | 0.10 | -3.02 | GLM |
| TGW | 2020GY | Chr09 | 47439717 | 1.85E-08 | 7.73 | 0.09 | -3.54 | GLM |
| TGW | 2020GY | Chr09 | 47439829 | 5.41E-09 | 8.27 | 0.10 | -3.11 | GLM |
| TGW | 2020GY | Chr09 | 47441861 | 1.13E-08 | 7.95 | 0.10 | -3.20 | GLM |
| TGW | 2020GY | Chr09 | 47453177 | 3.25E-08 | 7.49 | 0.09 | -3.20 | GLM |
| TGW | 2020GY | Chr09 | 47453421 | 7.86E-09 | 8.10 | 0.10 | -3.07 | GLM |
| TGW | 2020GY | Chr09 | 47454100 | 1.48E-08 | 7.83 | 0.10 | -3.08 | GLM |
| TGW | 2020GY | Chr09 | 47455366 | 3.89E-09 | 8.41 | 0.10 | -3.18 | GLM |
| TGW | 2020GY | Chr09 | 47456327 | 1.22E-08 | 7.91 | 0.11 | -2.96 | GLM |
| TGW | 2020GY | Chr09 | 47456336 | 1.22E-08 | 7.91 | 0.11 | -2.96 | GLM |
| TGW | 2020GY | Chr09 | 47459270 | 1.31E-08 | 7.88 | 0.11 | -2.94 | GLM |
| TGW | 2020GY | Chr09 | 47459472 | 2.17E-09 | 8.66 | 0.11 | -3.17 | GLM |
| TGW | 2020GY | Chr09 | 47470514 | 2.74E-08 | 7.56 | 0.12 | -2.84 | GLM |
| TGW | 2020GY | Chr09 | 47470625 | 3.20E-08 | 7.50 | 0.12 | -2.88 | GLM |
| TGW | 2020GY | Chr09 | 47472616 | 4.17E-08 | 7.38 | 0.11 | -2.82 | GLM |
| TGW | 2020GY | Chr09 | 47508638 | 2.75E-08 | 7.56 | 0.12 | -2.87 | GLM |
| TGW | 2020GY | Chr09 | 47512878 | 2.06E-08 | 7.69 | 0.12 | -2.84 | GLM |
| TGW | 2020GY | Chr09 | 47512898 | 4.68E-08 | 7.33 | 0.12 | -2.79 | GLM |
| TGW | 2020LD | Chr10 | 11199738 | 8.25E-10 | 9.08 | 0.12 | NA | Blink |
| TGW | 2018HZ | Chr10 | 45169097 | 8.15E-15 | 14.09 | 0.31 | NA | Blink |
| TGW | 2018GY | Chr10 | 45522047 | 7.83E-10 | 9.11 | 0.21 | NA | Blink |
| PT | 2020GY | Chr02 | 61994517 | 3.06E-09 | 8.51 | 0.12 | -14.15 | GLM |
| PT | 2020GY | Chr02 | 61982014 | 6.16E-09 | 8.21 | 0.11 | -14.30 | GLM |
| PT | 2020GY | Chr02 | 61985079 | 6.68E-09 | 8.18 | 0.11 | -13.91 | GLM |
| PT | 2020GY | Chr02 | 61992841 | 6.85E-09 | 8.16 | 0.12 | -13.74 | GLM |
| PT | 2020GY | Chr02 | 61992842 | 6.85E-09 | 8.16 | 0.12 | -13.74 | GLM |
| PT | 2020GY | Chr02 | 61992846 | 6.85E-09 | 8.16 | 0.12 | -13.74 | GLM |
| PT | 2020GY | Chr02 | 61994947 | 7.15E-09 | 8.15 | 0.12 | -13.56 | GLM |
| PT | 2020GY | Chr02 | 61990223 | 7.2E-09 | 8.14 | 0.12 | -13.81 | GLM |
| PT | 2020GY | Chr02 | 61985068 | 7.55E-09 | 8.12 | 0.11 | -13.94 | GLM |
| PT | 2020GY | Chr02 | 61991138 | 7.86E-09 | 8.10 | 0.12 | -14.08 | GLM |
| PT | 2020GY | Chr02 | 61992827 | 8.51E-09 | 8.07 | 0.12 | -13.63 | GLM |
| PT | 2020GY | Chr02 | 61985111 | 8.69E-09 | 8.06 | 0.10 | -14.86 | GLM |
| PT | 2020GY | Chr02 | 61981972 | 9.36E-09 | 8.03 | 0.11 | -13.84 | GLM |
| PT | 2020GY | Chr02 | 61989805 | 1.09E-08 | 7.96 | 0.13 | -13.24 | GLM |
| PT | 2020GY | Chr02 | 61993577 | 1.35E-08 | 7.87 | 0.12 | -13.37 | GLM |
| PT | 2020GY | Chr02 | 61994684 | 1.47E-08 | 7.83 | 0.13 | -12.81 | GLM |
| PT | 2020GY | Chr02 | 61992035 | 1.51E-08 | 7.82 | 0.12 | -13.04 | GLM |
| PT | 2020GY | Chr02 | 61994405 | 1.51E-08 | 7.82 | 0.12 | -13.62 | GLM |
| PT | 2020GY | Chr02 | 61990591 | 1.57E-08 | 7.80 | 0.12 | -12.98 | GLM |
| PT | 2020GY | Chr02 | 61992996 | 1.59E-08 | 7.80 | 0.12 | -13.37 | GLM |
| PT | 2020GY | Chr02 | 61998712 | 1.63E-08 | 7.79 | 0.13 | -13.03 | GLM |
| PT | 2020GY | Chr02 | 61990271 | 1.99E-08 | 7.70 | 0.12 | -13.19 | GLM |
| PT | 2020GY | Chr02 | 61985052 | 1.99E-08 | 7.70 | 0.11 | -13.33 | GLM |
| PT | 2020GY | Chr02 | 61990184 | 2.05E-08 | 7.69 | 0.12 | -13.17 | GLM |
| PT | 2020GY | Chr02 | 61990193 | 2.05E-08 | 7.69 | 0.12 | -13.17 | GLM |
| PT | 2020GY | Chr02 | 61993077 | 2.17E-08 | 7.66 | 0.11 | -13.64 | GLM |
| PT | 2020GY | Chr01 | 68394108 | 4.82E-08 | 7.32 | 0.27 | 4.31 | GLM |
| PT | 2020GY | Chr04 | 42808879 | 3.56E-08 | 7.45 | 0.10 | -6.54 | GLM |
| PT | 2020GY | Chr04 | 42808880 | 4.45E-08 | 7.35 | 0.09 | -6.61 | GLM |
| TT | 2020LD | Chr02 | 8300524 | 2.15E-08 | 7.67 | 0.10 | 0.04 | GLM |

**Supplemental Table 6** Correlation analysis results between mesocarp thickness and grain size and shape in six environments

Note: TGW - Thousand Grain Weight, GL - Grain Length, GW - Grain width, PT-Pericarp Thickness, TT - Testa Thickness

| **Traits** | **Years** | **Sites** | **R value with MT** | **Significant Level** |
| --- | --- | --- | --- | --- |
| TGW | 2018 | Guiyang | 0.267 | P<0.01 |
| TGW | 2018 | Hangzhou | 0.216 | P<0.01 |
| TGW | 2019 | Guiyang | 0.289 | P<0.01 |
| TGW | 2019 | Hangzhou | 0.248 | P<0.01 |
| TGW | 2020 | Guiyang | 0.300 | P<0.01 |
| TGW | 2020 | Hangzhou | 0.195 | P<0.05 |
| TGW | 2020 | Ledong | 0.313 | P<0.01 |
| TGW | 2020 | Lingshui | 0.279 | P<0.01 |
| GL | 2018 | Guiyang | 0.183 | P<0.01 |
| GL | 2018 | Hangzhou | 0.253 | P<0.01 |
| GL | 2019 | Guiyang | 0.301 | P<0.01 |
| GL | 2019 | Hangzhou | 0.322 | P<0.01 |
| GL | 2020 | Guiyang | 0.388 | P<0.01 |
| GL | 2020 | Hangzhou | 0.374 | P<0.01 |
| GL | 2020 | Ledong | 0.404 | P<0.01 |
| GL | 2020 | Lingshui | 0.309 | P<0.01 |
| GW | 2018 | Guiyang | 0.070 | 0.2687 |
| GW | 2018 | Hangzhou | 0.240 | P<0.01 |
| GW | 2019 | Guiyang | 0.267 | P<0.01 |
| GW | 2019 | Hangzhou | 0.248 | P<0.01 |
| GW | 2020 | Guiyang | 0.265 | P<0.01 |
| GW | 2020 | Hangzhou | 0.276 | P<0.01 |
| GW | 2020 | Ledong | 0.278 | P<0.01 |
| GW | 2020 | Lingshui | 0.268 | P<0.01 |
